# Cooperativity-based modeling of heterotypic DNA nanostructure assembly

**DOI:** 10.1093/nar/gkv602

**Published:** 2015-06-13

**Authors:** Anastasia Shapiro, Avital Hozeh, Olga Girshevitz, Almogit Abu-Horowitz, Ido Bachelet

**Affiliations:** Faculty of Life Sciences and Institute of Nanotechnology & Advanced Materials, Bar-Ilan University, Ramat Gan 52900, Israel

## Abstract

DNA origami is a robust method for the fabrication of nanoscale 2D and 3D objects with complex features and geometries. The process of DNA origami folding has been recently studied, however quantitative understanding of it is still elusive. Here, we describe a systematic quantification of the assembly process of DNA nanostructures, focusing on the heterotypic DNA junction—in which arms are unequal—as their basic building block. Using bulk fluorescence studies we tracked this process and identified multiple levels of cooperativity from the arms in a single junction to neighboring junctions in a large DNA origami object, demonstrating that cooperativity is a central underlying mechanism in the process of DNA nanostructure assembly. We show that the assembly of junctions in which the arms are consecutively ordered is more efficient than junctions with randomly-ordered components, with the latter showing assembly through several alternative trajectories as a potential mechanism explaining the lower efficiency. This highlights consecutiveness as a new design consideration that could be implemented in DNA nanotechnology CAD tools to produce more efficient and high-yield designs. Altogether, our experimental findings allowed us to devise a quantitative, cooperativity-based heuristic model for the assembly of DNA nanostructures, which is highly consistent with experimental observations.

## INTRODUCTION

Structural DNA nanotechnology, which makes use of DNA as a tractable building block for the construction of arbitrary shapes by molecular self-assembly, has proven an efficient route for building new functional objects with increasing complexity (1–7). In fact, it consists of a diverse group of approaches that use DNA for nanofabrication, including the various techniques collectively known as DNA origami (8,9); but, with some notable exceptions (10,11), most of these techniques are based in some way on the immobile DNA junction described by Seeman (12,13). The easy implementation of junction design rules in a computer code enabled the development of CAD tools that simplified the task of designing large, complex structures containing hundreds of connected junctions (14–17).

The process of DNA origami folding has been recently studied to gain better understanding of the process but also to enable its optimization for efficient, industrial scale synthesis of DNA nanostructures (18,19). Interestingly, the folding of scaffolded DNA origami structures was found to occur mostly around a critical temperature that depends on the structure itself (18). The critical temperature has to be empirically found, but once it has, folding can be carried out by simply maintaining the DNA mixture at that temperature for a relatively short period of time. This process was also visualized using atomic force microscopy (AFM) (20,21). Another recent study reported the kinetics of assembly of individual DNA strands within an origami structure (22). However, good theoretical quantitative understanding of the folding kinetics of large DNA structures is still missing, with very little known about how it is driven from the early assembly events of its basic units.

We refer to the first junctions created, for example the archetypal junction J1 (12), as being ‘homotypic’ or nearly homotypic, meaning that arm lengths and melting temperatures are identical nearly identical. J1 has been designed to maximize assembly efficiency and rate. However, homotypic junctions represent private cases of heterotypic junctions, as the former comprise a subset of the larger set of possible heterotypic random junctions. Since homotypy is a constraint, heterotypic junctions would likely predominate in DNA origami structures as they become increasingly complex. Moreover, homotypic junctions form from their individual components—the arms—within a very small temperature range, making the study of their formation process challenging. In contrast, heterotypic junctions can be designed to maximize the difference between discrete formation events, enabling to study in higher resolution the process of junction formation. Moreover, it can show how a formed junction impacts the formation of a neighboring junction by cooperativity, or alternatively, inhibiting it by interfering with its freedom to assemble.

In this study, we focused on the heterotypic DNA junction as an experimental system to quantitatively study how early, local events drive global assembly of DNA structures. We designed junction-based devices to track the routes and directions in which formation events drive subsequent ones, using bulk fluorescence measurements and AFM. We report here a systematic study of heterotypic junction formation, and demonstrate that the rapid and efficient folding of scaffolded DNA origami structures is driven by cooperativity at multiple scales. Interestingly, we highlight consecutiveness a new design consideration for DNA nanostructures, stressing the positioning order of components in DNA junction systems. Our measurements allowed us to devise a quantitative heuristic model describing the folding of DNA origami structures, which is highly consistent with experimental observations by us and others. This model could predict the critical temperature of folding, enabling a-priori optimization of folding conditions for industrial scale DNA nanofabrication.

## MATERIALS AND METHODS

### Computer-aided design

DNA junctions were designed using Uniquimer2D (15). DNA origami shapes were designed using caDNAno (16), using the 7249 base-long M13mp18 genome as scaffold strand. DNA staple strands were edited manually as necessary. Finite element analysis of designed objects was performed by CANDO (17).

### DNA folding

DNA scaffolds were ordered from either New England BioLabs or Tilibit Nanosystems and stored at −20°C. Staple strands were ordered from either IDT or IBA life sciences, and reconstituted to 100 μM with DNase/RNase free DDW (Life technologies) and stored at −20°C. All DNA sequences are listed in Supplementary note 1. Scaffold and staple strands were mixed at a 1:10 ratio (scaffold concentration 10 or 20 nM) in TAE buffer (40 mM Tris-acetate, 1 mM EDTA at pH 8.3) or folding buffer (5 mM Tris, 1 mM EDTA, 5 mM sodium chloride at pH 8) with varying concentrations of magnesium chloride (ranging from 10 to 24 mM). Mixtures were folded in a thermal cycler (BioRad C1000 Touch Thermal Cycler) according to the following sequence: 5 min at 65°С, followed by stepwise cooling to 60°С at −1°С/15 min, followed by stepwise cooling to 25°С at −1°С/3 h. For measurements of folding inside the rectangle variant (described below), scaffold and staple strands were mixed at a 1:2 ratio (scaffold concentration: 80 nM) in TAE buffer with 18 mM magnesium chloride, and folded according to the following protocol: 5 min at 85°С, followed by stepwise cooling to 60°С at −1°С/15 min, followed by stepwise cooling to 25°С at −1°С/3 h. Folding reactions of single DNA junctions (K1, K2, K3’, K3 and J1, described below) and their parts (arms alone, two arm complexes) by mixing the components in equimolar concentrations of 1 μM in TAE buffer with 10 mM magnesium chloride. For assembly of 2J and 4J systems (described below), reactions were carried out as folding reactions for single DNA junction, but with 12 mM magnesium chloride. Both were folded according to desired thermal ramping protocols: for K1, K2, K3’, K3 and J1 junctions : 5 min at 95°С, followed by stepwise cooling to 25°С at −1°С/2.5 min. For 2J and 4J models: 5 min at 95° С, followed by stepwise cooling to 4°С at −1°С/2.5 min. Following folding, DNA origami shapes were purified on 100 K Amicon ultra–0.5 ml centrifugal filter units with Ultracel-100 membrane (100 kDa molecular weight cutoff). Single junctions, 2J and 4J systems were not purified after folding.

### Fluorescence measurements

Fluorophore/quencher-modified DNA strands were ordered from either IDT or IBA life sciences at various positions as described in the results section. Fluorescence measurements were taken during the folding process/annealing ramp of the structures (described above in ‘DNA folding’ section), and were performed in two different quantitative real-time PCR instruments (Bio-Rad, Thermo Scientific) to eliminate instrument bias.

### Gel electrophoresis

DNA folded samples were electrophoresed on 0.5× TBE agarose gels stained with ethidium bromide purchased from Sigma and 11 mM magnesium chloride in an ice- water bath (80–100 V, 3–5 h). The running buffer contained 0.5× TBE and 11 mM MgCl_2_. DNA origami structures were run on 1.5% agarose gels with 1 kb DNA ladder. The TBE buffer and the Agarose LE were ordered from Promega, while the gel loading dye and DNA ladders were purchased from New England BioLabs. Gels were imaged on an BioRad Gel Doc Imager and analyzed on ImageLab v4.0.1 software.

### Atomic force microscopy

After completing the folding process, reconstructed rectangle structures were imaged with two different instruments: a Bruker Bio-FastScan (tapping mode using FASTSCAN-Dx cantilevers with force constant of 0.25 N/m) and a JPK NanoWizard ultra AFM III (scan modes AC and HyperDrive using ultra-short cantilevers with force constant of 0.3 N/m). Imaging was done in TAE buffer at room temperature with varying concentrations of magnesium chloride (12–20 mM), most scans were performed in 12 mM. Freshly cleaved mica was treated with 4 mM nickel chloride prior to sample deposition. Images were analyzed using Nanoscope analysis v1.5 and JPK Data Processing v4.3.26.

### Simulations and mathematical modeling

Assembly simulations and *T*_m_ prediction for single junction, 2J and 4J experiments were carried out in NUPACK (23), and results were adjusted to an experimentally-obtained correlation curve. Model was written in Perl (http://www.perl.org/). The model is compatible with DNA shapes designed by caDNAno, taking two inputs from the user: (i) the caDNAno .json file and (ii) the .csv staple list exported by caDNAno as the final stage of the design process. Before running the model, the files are converted into a .txt format. In addition, the annealing temperature range must be inserted by the user. The algorithm parses these files and based on our findings calculates the critical temperature and folding profile of a given shape. The obtained output of the modeling is analyzed on Excel and MATLAB v8.0 (http://www.mathworks.com/products/matlab/) to create a heat map of the analyzed shape or to simulate the folding process. Code is listed in Supplementary note 6.

## RESULTS AND DISCUSSION

### Assembly of single heterotypic junctions proceeds through key events

**Figure 1.**
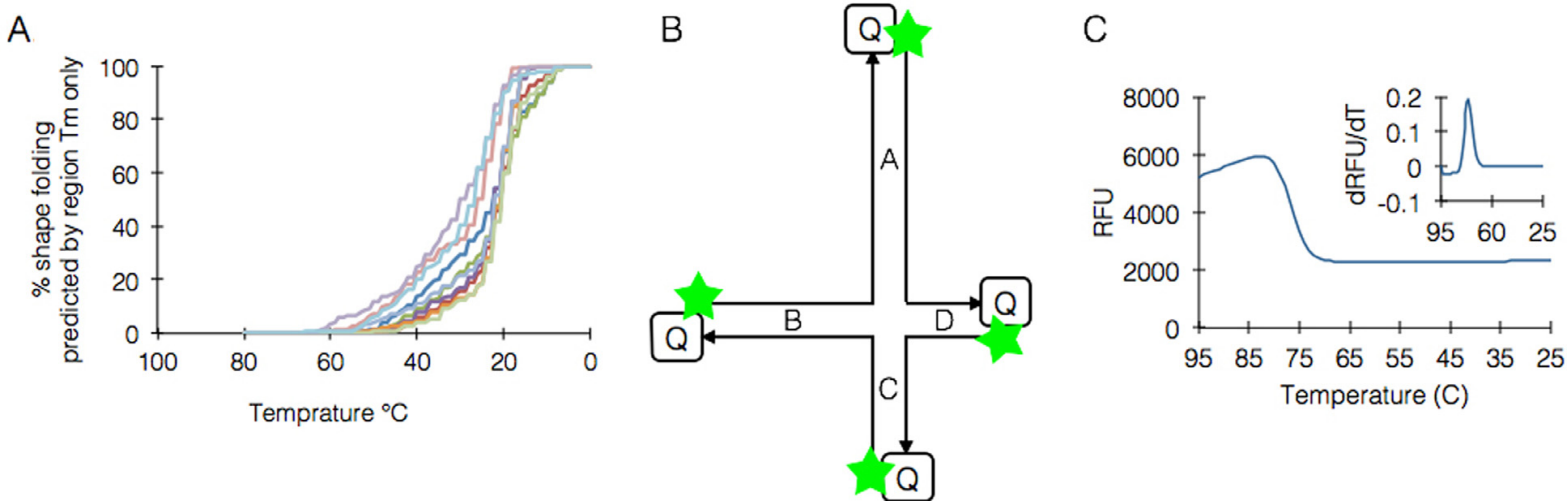
*T*_m_ distribution and experimental approach. (**A**) an overlay analysis of staple region *T*_m_ distribution in various DNA origami shapes designed by caDNAno; shapes include gear-like object, brick, and single-layer rectangle (18) in addition to four 3D shapes and two 2D shapes we have designed. Predicted only by region *T*_m_, the graph shows that most regions in all shapes fall between 24 and 30°C. (**B**) the basic design of K1 junction. A–D denote arms, green stars denote FAM and [Q] denotes a dark quencher (FQ). (**C**) Typical measurement of arm formation event by fluorescence. Large graph shows RFU while inset graph shows derivative analysis.

Our starting point was the view of DNA junctions as basic subunits of more complex DNA nanostructures, in contrast to previous reports (22). Large scaffolded DNA origami structures are comprised of hundreds of discrete staple-scaffold regions, with unclear cross-dependency. Analysis of region *T*_m_ distribution shows that most of them, in almost all shapes, fall within a limited range of 24–30°C, which is approximately 30°C lower than the actual temperature in which the majority of the folding process takes place (18) (Figure 1A).

To enable precise, time-resolved tracking of the assembly process of DNA junctions, we designed a prototype junction with large gaps between the predicted formation temperatures of discrete events during junction assembly, in contrast to previously reported junctions. Except for its convenience as an experimental system, heterotypic junctions are the general case of homotypic junctions. This junction, denoted K1, contains 4 arms A, B, C and D (with 34, 23, 13 and 10 bp, respectively) which were dually labeled with fluorophores and dark quenchers (Figure 1B). We used FAM on each arm and performed 4 separate experiments with a different arm labeled in each, in order to avoid fluorophore-specific effects, which were observed early on (Supplementary note 2). The formation of K1 by a thermal annealing ramp was measured in a quantitative PCR instrument by tracking the signal of each fluorophore at each temperature, with 50% signal corresponding to the arm's *T*_m_ (Figure 1C).

**Figure 2.**
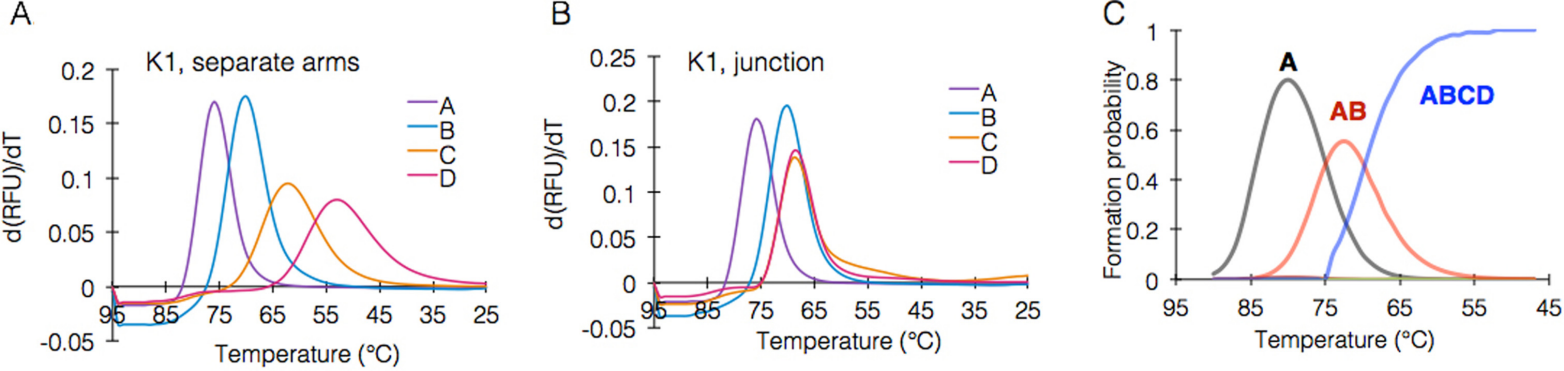
Assembly of K1 proceeds through key events. (**A**) Fluorescence analysis of the formation of arms A to D in K1, formed in four separate experiments. (**B**) fluorescence analysis of the formation of the complete K1 junction. (**C**) NUPACK simulation of the formation sequence of K1, letters denote arm complexes forming in order.

The formation of separate arms (Figure 2A) correlated well with predictions made by NUPACK (23). However, the entire K1 structure formed at a significantly higher temperature (69°C) and within a smaller temperature range (Figure 2B). We defined this temperature as the junction formation temperature, or *T_j_*, of K1. Simulations of the formation of intermediate species in K1 at each temperature showed that the first event is the formation of arm A. Once ∼50% of the strands that build arm A are hybridized, the arm complex AB starts to form directly, bypassing the formation of the separate arm B, which is negligible. Finally, once ∼50% of the strands that build the complex AB are hybridized, the whole junction starts to form (Figure 2C). The analysis correlated well with our observations, which showed that the arm complex AB was formed and not arm B alone, and that this complex accounts for nearly all the fluorescent signal. Complexes BC and CD also formed, indicating that the signals obtained from arms C and D during K1 formation correspond with formation of ABC and ABCD structures, respectively (Figure 2B). Interestingly, there is a consistent difference of 1°C between the annealing and melting of K1, similar to (but smaller than) the hysteresis reported previously in DNA origami structures (18). These findings so far demonstrate that DNA junction formation proceeds through key events, with each event facilitating the formation of subsequent ones.

### Arm position order affects assembly route and efficiency

**Figure 3.**
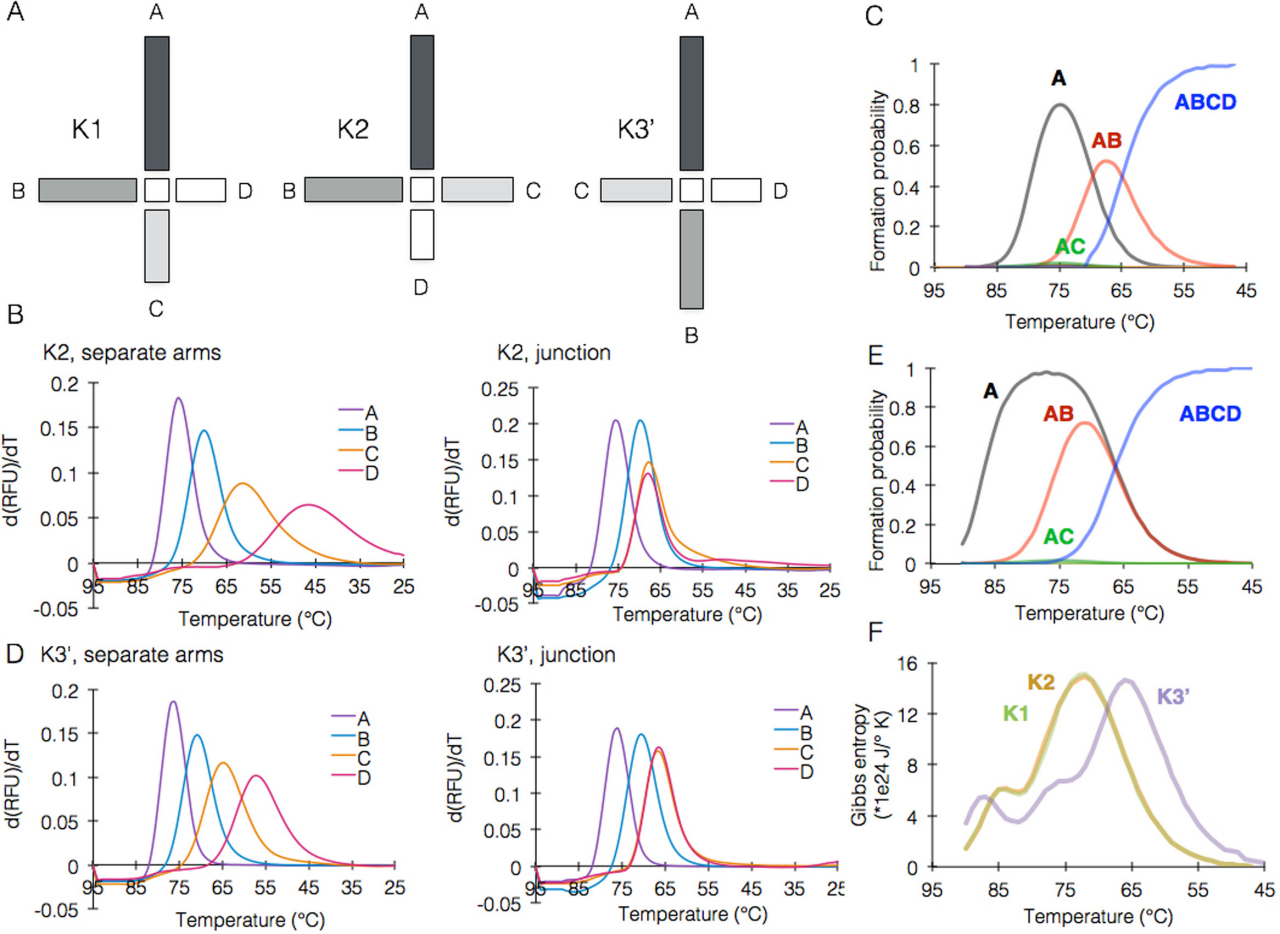
Arm position order affects assembly route and efficiency. (**A**) schematic representations of K1 and its two reshuffled variants K2 and K3’. (**B**) fluorescence analysis of the formation of K2 in separate arms (left) versus complete junction (right). (**C**) NUPACK simulation of the formation sequence of K2. (**D**) Fluorescence analysis of the formation of K3’ in separate arms (left) versus complete junction (right). (**E**) NUPACK simulation of the formation sequence of K3’. (**F**) Simulated entropic landscapes of the formation process of K1, K2, and K3’ showing the similarity between K1 and K2, and variation of K3’.

Folding of K1 proceeds according to the sequence A > AB > ABCD. However, this sequence of events is derived from the design of K1 (ABCD), in which consecutive arms are ordered according to their length and *T*_m_. To examine how this order affects junction formation, we designed two junction variants termed K2 and K3, in which the arms were reshuffled to break their consecutive order without changing any sequence. In K2 we swapped between arms C and D (ABDC) while in K3 between arm B and C (ACBD). Hence, consecutive order in K2 is violated once, while in K3 it is violated twice. One modification was nevertheless necessary because reshuffling of arms B and C in K3 resulted in a single base pair (G–C) symmetry abutting the junction center, which is forbidden by Seeman's rules for immobile junction design (12). To correct this situation, we swapped the G–C base pair to C–G, thereby eliminating the symmetric base pair while not affecting *T*_m_ or length. This junction was termed K3’ (Figure 3A, Supplementary note 3). In K2, folding of the separate arms or the entire junction (ABDC) proceeded essentially similar to K1, however the *T_j_* was slightly lower (68°C, *P* < 0.0001). As seen in K1, the observed *T*_m_ in the context of the full junction was higher and assembly occurred again within a smaller temperature range (Figure 3B). In addition, the results corresponded well with the simulated formation of every species in K2 at each temperature: arm A first, once ∼60% of the strands that build A are hybridized, the arm complex AB starts to form, while the formation of B as a separate arm is negligible. Finally, once ∼50% of the strands that build the complex AB are hybridized, the arm complex ABDC (the full junction) starts to form. Interestingly, in contrast to K1, K2 simulation demonstrated a significant formation of the intermediate complex AC, suggesting that a very small fraction of the junctions population assembles via an alternative trajectory (Figure 3C), which could explain the significant albeit slight difference in *T_j_* between K1 and K2.

In contrast to K1 and K2, the order K3’ is violated twice (ACBD). The same trend of separate arms versus full junction was observed here (Figure 3D). However, the observed *T_j_* of K3’ was 67°C. Intriguingly, the simulation showed a different pattern than K1 and K2, with arms A and B folding separately and the full junction ACBD starting to form when B reaches approx. 50%, indicating that the folding order is A > B > ACBD (Figure 3E) and showing here too that some junctions proceed to assembly via alternative trajectories, likely accounting for the lower efficiency observed. Annealing-melting hysteresis of 1°C was also observed in K2 and K3’, showing that this is likely an inherent property of the structure not affected by arm order.

Even though the observed differences in *T_j_* between K1, K2 and K3’ are only 1°C, these differences are significant and repeated precisely regardless of instrument or fluorophore. Moreover, they are cumulative in a large shape where junctions feed into subsequent junctions, and might affect the efficiency of folding of the entire shape.

### The entropy landscape of the junction assembly process

NUPACK simulations, which are highly consistent with our experimental observations, produce a probability distribution of each intermediate species at each temperature, enabling us to calculate a theoretical Gibbs entropy landscape using the equation:

\begin{equation*} S = - k_{\rm B} \sum {p\;{\rm ln}(p)} \end{equation*}

where *S* is the Gibbs entropy, *k*_B_ is the Boltzmann constant (1.38 × 10^−23^ J/K) and *p* is the formation probability of an intermediate species (either separate arm or an arm complex), given by the concentration of each species relative to the total concentration of precursor strands (1 μM). Based on this simulation, the process of K1 assembly is at maximal number of microstates at 72°C, after which entropy falls rapidly (Figure 3F). We suggest that up to this point, the temperature decrease allows the necessary precursors for junction formation to build up, especially the immediate precursor AB, which reaches a critical mass at exactly this temperature. The entropy landscape of K2 is identical to K1, while that of K3’ (and K3) is significantly shifted to the right with peak theoretical entropy at 66°C consistent with the observed behavior of this system.

### Cooperativity between junctions in a bijunction structure

**Figure 4.**
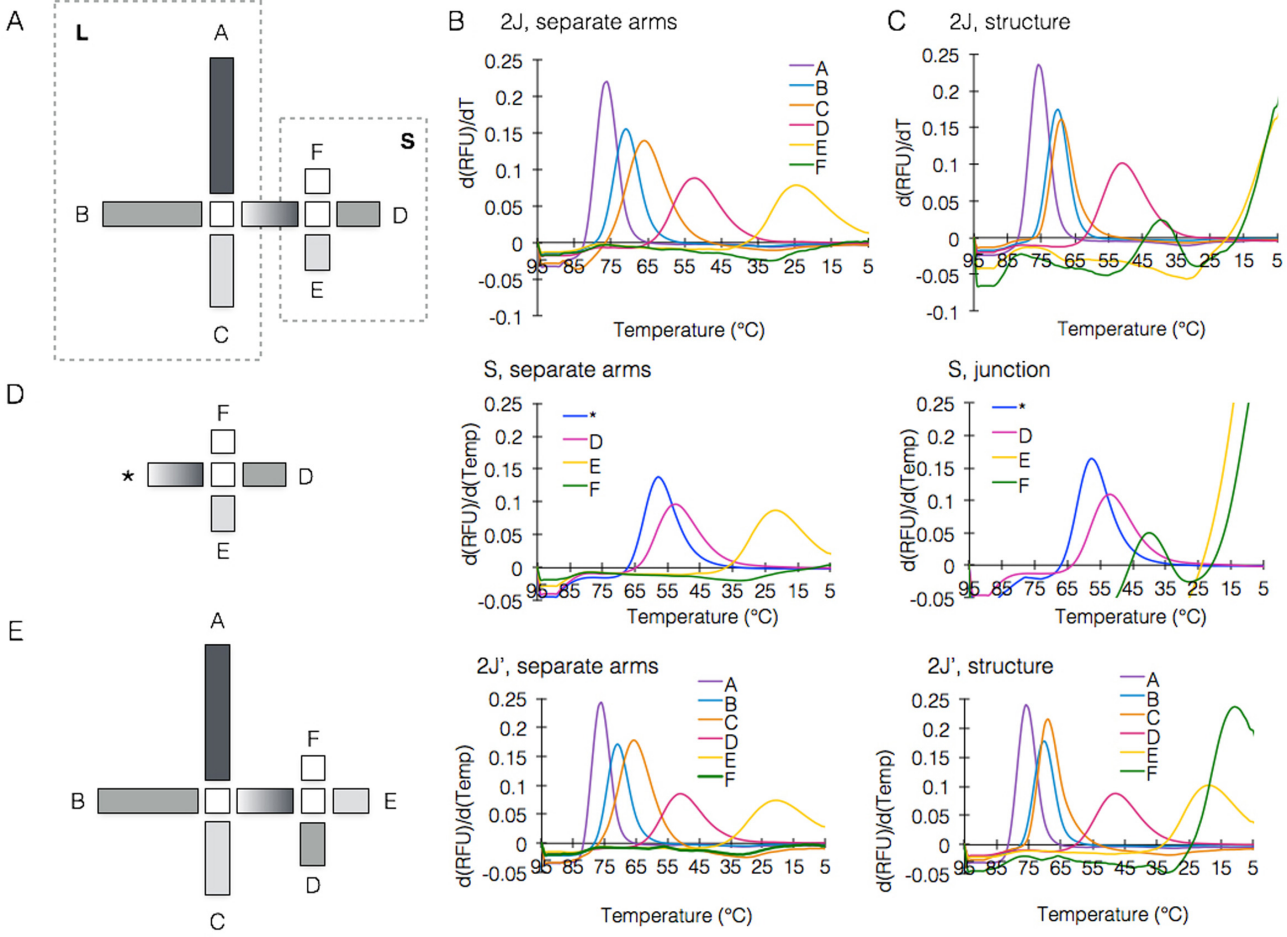
Cooperativity between junctions in a bijunction structure. (**A**) schematic representation of the bijunction structure 2J formed from K1 (termed L) with the smaller junction termed S. (**B**) Fluorescence analysis of the formation of 2J in separate arms. (**C**) fluorescence analysis of the formation of the complete 2J structure. (**D**) fluorescence analyses of the formation of the smaller junction in 2J separately versus complete structure. The * symbol represents the linker arm between the L and S junctions in 2J structure. (**E**) Fluorescence analyses of the formation of 2J’ (with S fixed to a K1-type junction), separately versus complete structure.

Our next step was to study the interactions between two junctions in a twin (bi)-junction structure, denoted 2J. Since the *T*_m_ and entropy profiles of K1 were known, we used it as the basis for 2J design, to which a smaller junction was appended. We named the K1 junction in 2J structure L, and the new added junction S. To ensure that the folding starts from L junction, the lengths of the arms in S were shortened and designed with lower predicted *T*_m_. The predicted folding order of 2J is ABCDEF (Figure 4A). We repeated the same set of experiments as we did for single junctions analyses. First we examined the folding of the separate arms by exposing the strands to a temperature-annealing ramp. The measured *T*_m_ correlated well with predicted *T*_m_. As predicted, arm F was not folded (Figure 4B). Next, the arms were mixed to build the 2J structure. These results showed that L junction arms (arms A, B and C) folded at almost the same *T*_m_ as in K1 as expected, whereas in S junction, arm E was not folded. However, arm F folded at surprisingly high *T*_m_ (44°С) (Figure 4C).

To better understand these results, we examined S junction separately. As can be seen from the design and the predicted *T*_m_ of each arm, S junction is a K3 type junction, meaning the folding order is violated twice (*EDF). Comparing the formation of each arm separately to each arm in S junction revealed, as expected, that S junction was not formed (Figure 4D).

In order to strengthen our consolidating assumption that junctions of junction types K1 and K2 are more stable and better to use in DNA origami shapes, we redesigned the 2J structure to have two K1 type junctions instead of combining K1 and K3, terming it 2J’. We transformed S junction from being K3 to K1 type by reshuffling arms D and E. The sequences remained the same and were labeled as described previously. The results of 2J’ obtained by repeating the same set of experiments supported our assumption and demonstrated that while S junction (K3 type) did not fold in 2J, it folded completely in 2J’ structure (Figure 4E).

These results show that the bijunction structure built from K1 type junction is more stable and likely to form in higher *T*_m_ than structures built of K3 type junctions as well, which may result in partially assembled structure. These results demonstrate the importance of consecutiveness also at the multiple junction scale, indicating that junction type is an important factor that should be considered while designing more complex DNA origami shapes.

### Cooperativity between junctions in a DNA origami shape

Finally, we designed a series of experiments to measure the cooperativity between heterotypic junctions in the folding of a large DNA origami shape. The method here was to redesign an existing DNA origami shape such that in a specific series of neighboring junctions subsequent events are isolated and set to occur in a temporally-resolved, measurable cascade. The object we chose was the single layer rectangle described by Sobczak (18), a standard shape derived from one of the first shapes folded successfully in high yields (9). These rectangles are composed almost entirely of identical junctions, making their redesign simpler for our experimental system.

**Figure 5.**
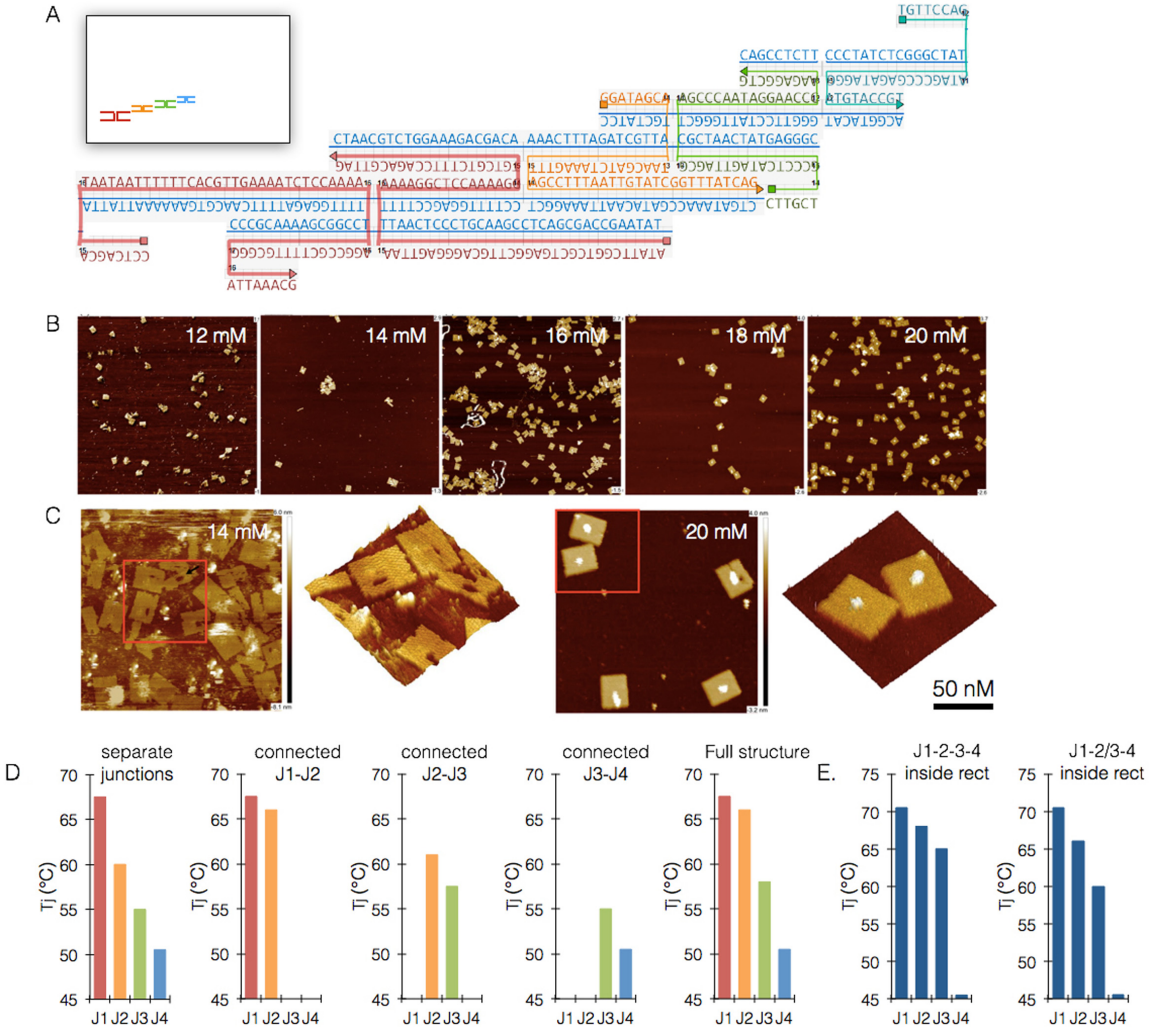
Cooperativity between junctions in a DNA origami shape. (**A**) Schematic design of the 4-junction structure inside the single layer rectangle, with inset showing relative position inside the object, and the magnified structure on the right. (**B**) AFM images showing folding of the modified rectangle at various Mg concentrations. (**C**) Magnified AFM images of the folded modified rectangle, showing the hypothetic missing 4J structure at lower concentrations, and the intact rectangle at 20 mM Mg. (**D**) Formation of the 4J structure outside the rectangle, from left to right: formation of all junctions separately, formation of connected junction pairs J1–J2, J2–J3, J3–J4 and complete structure, all showing the increased formation temperature due to cooperativity between junctions. (**E**) Formation of 4J inside the rectangle, with left graph showing intact formation sequence, and right graph showing sequence violation due to the removal of a single arm from J2.

To enforce folding initiation from a specific position in the rectangle, all staples in the shape were broken to a length of 16 bases (besides the five staples that build the series of neighboring junctions), creating folding regions of 8 bases only with a lower theoretical *T*_m_ (based on NUPACK and the corrected standard equation we used for *T*_m_ calculation, see Supplementary note 6). Therefore, the junctions created by these folding regions (i.e. arms) had a low theoretical *T_j_* (∼15–40°C). For designing the 4 neighboring junctions, a sub-structure named 4J, we chose a single junction proximal to one of the corners and reconstructed it by extending its staple strands (Figure 5A). Thus, we achieved a theoretical *T_j_* of 66°C for this junction, significantly higher than any other junction in the shape. Moving from this junction on, a series of consecutive junctions was built such that each junction shares an arm with the previous junction and the next one in the cascade, having a slightly lower predicted *T_j_*. The four junctions were termed J1, J2, J3 and J4. The fourth junction had the lowest *T_j_* and was in fact similar to the rest of the junctions in the rectangle outside the 4J system, serving as an internal control. In this set of experiments each junction was labeled diagonally, since in the DNA origami shape, specific bases cannot be labeled in the scaffold strand. In order to get a full resolution picture of the folding cascade we labeled each junction twice on the right and left diagonals (Supplementary note 4). The double diagonal labeling was expected to yield signal only when junction forms, and a certain degree of variation was expected between the two measurements because junctions in the DNA origami shape are built differently than the isolated junctions K1–K3 in the sense that the scaffold strand leads to different kinetics than that of oligos alone. Therefore, the *T_j_* of a junction was set as the average of the two diagonal measurements.

Screening for optimal folding conditions for the reconstructed rectangle highlighted 1– Tris-acetate–EDTA buffer with 18–20 mM magnesium. Folding of the shapes was validated by gel electrophoresis (Supplementary note 5) and AFM (Figure 5B). Interestingly, in suboptimal conditions (≤16 mM magnesium), the rectangles exhibited a missing part likely corresponding to the 4J structure, demonstrating that we successfully created a physically distinct region within the shape (Figure 5C).

First we examined the behavior of this system outside the context of the rectangle. We split this system to the previously examined scales: single junction and bijunctions, and added a new scale of four junctions. The double-diagonal labeling produced the same *T_m_* for both sides; however, J2 showed at times 8°С difference between the two diagonal readings of this junction, likely due to the existence of two alternative trajectories for folding the junction instead of one. *T_j_* was calculated as the average between these two measurements. The results showed that junction folding order was according to our design and the predicted *T_j_*: J1 > J2 > J3 > J4. Moreover, there was striking similarity between the patterns of events across the different scales, single junction versus the bijunction and 4J system. When formed separately, each *T_j_* was significantly lower than the *T_j_* of the same junction in the bijunction and 4J system. In all bijunction variants and the 4J system, the left junction is the ‘seed’ junction in the cascade, therefore its *T_j_* is nearly equal to the measured *T_j_* when formed separately. Moreover, information flow is sequential: J1 seeds more information to J2, than J2 to J3. J3 did not facilitate the formation of J4, and the latter's *T_j_* remained the same in all examined systems. When specific parts of the system were removed from between J2 and J3, the *T_j_* of J3 decreased and equaled that of the J3 folded alone (Figure 5D). Altogether, these findings indicate that the cooperativity between two neighboring junctions is a function of the seeding junction's *T_j_* and the difference in size and *T_j_* between the two junctions. The higher the *T_j_* of the seeding junction, and the bigger the differences between the *T_j_*, the higher the impact of this cooperative effect.

Finally, we tracked the folding of 4J integrally inside the reconstructed rectangle. It is important to note that in a typical scaffolded origami folding protocol the staples are at a molar excess over scaffold, meaning most of them do not enter the shape and therefore part of the fluorescent signal would not be quenched. To minimize this noise effect, we used a staple to scaffold ratio of 1:2 and increased scaffold concentration to 80 nM. The observed junction folding order remained J1 > J2 > J3 > J4. However, partially knocking out the link between J2 and J3 led to a significantly lower *T_j_* of J3. The internal control J4 junction was not influenced by this knock-out (Figure 5E).

### A quantitative cooperativity-based model of DNA origami folding

Altogether, these findings enabled us to devise a quantitative model for the folding of DNA origami. Supplementary note 6 describes in detail how the model works and the mathematical implementations of the experimental data into actionable heuristics.

The model takes three inputs: (i) the caDNAno json file; (ii) the list of staples exported by caDNAno as the final stage of the design process and (iii) the annealing temperature range. The model breaks the shape into DNA junctions and single staples which are not part of any junction. It then applies heuristics based on experimental observations, and performs a comprehensive analysis of the shape, when the main and final results are the simulation of the kinetics during the folding process and prediction of the critical temperature. The model provides additional information such as more accurate prediction of folding region *T*_m_, predicted *T_j_* of each junction and the distribution of junction types across the shape blueprint—‘K1’, ‘K2’, ‘K3’ or ‘J1’, with the latter including nearly homotypic junctions, which, according to our observations behave similarly to homotypic ones.

**Figure 6.**
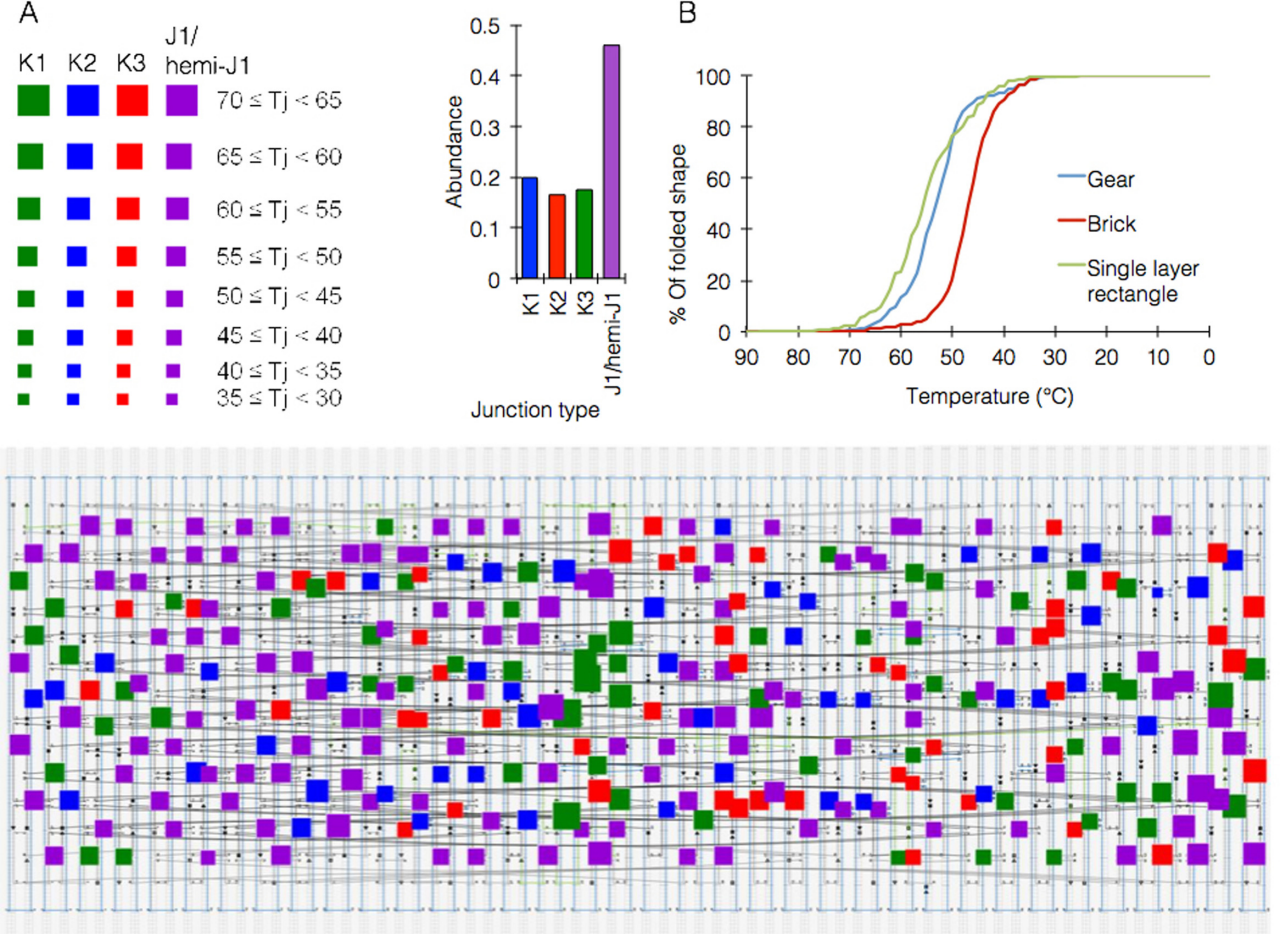
Folding model performance. (**A**) analysis of junction type distribution within a caDNAno blueprint of the gear-like object (16) (top table showing color and size coding of the squares, with each square overlaid on a junction in the structure). (**B**) model simulations of folding kinetics of three DNA origami shapes (model design is detailed in Supplementary note 6).

Importantly, since our model is partly based on predictions and simulations made in NUPACK, it was necessary to add a correction factor to adjust the predicted values to experimental ones. To this end we carried out a series of calibration experiments using various sequence motifs and lengths and using different qPCR instruments, to produce a correction function, which was then applied to all values and kinetics predicted by NUPACK (Figure 6A, Supplementary note 6).

To validate the model, we performed a comprehensive folding simulation on previously studied shapes (18) with known critical temperature of folding. Our model predicted a critical temperature range within 2.5 degrees of the observed temperatures, with accurate prediction for some shapes (Figure 6B). It was interesting to see that the different junction types across different shapes were not uniformly distributed, with some shapes lacking specific junction types altogether and others showing high bias towards a specific junction type.

## CONCLUSION

The immobile DNA junction has been the seed of the growing field of DNA nanotechnology. In this work we aimed to elucidate how these junctions function as actual seeds in the folding process of large DNA structure composed of hundreds of connected junctions. Our observations demonstrate that the assembly of DNA junctions and associated structures occurs as a cascade of discrete events that feed into each other depending on their proximity and continuity. Breaking the order of these events results in reduced stability. Moreover, the cross-talk between these assembly events is analyzed here, enabling quantitative understanding of this process and how it leads to global outcomes. It should be possible to implement the considerations highlighted in this study in existing tools for DNA design to improve future products.

## SUPPLEMENTARY DATA

Supplementary Data are available at NAR Online.

SUPPLEMENTARY DATA
