# Supplementary material for: Cooperativity-based modeling of heterotypic DNA nanostructure assembly
Source: Nucleic Acids Res. 2015 Jun 13;43(13):6587–95. doi: 10.1093/nar/gkv602 (PMC4513873; doi:10.1093/nar/gkv602)
Supplement: SUPPLEMENTARY DATA [file supp_43_13_6587__index.html]

Cooperativity-based modeling of heterotypic DNA nanostructure assembly — SUPPLEMENTARY DATA 

# Cooperativity-based modeling of heterotypic DNA nanostructure assembly

## SUPPLEMENTARY DATA

- SUPPLEMENTARY DATA
