# Supplementary material for: Cooperativity-based modeling of heterotypic DNA nanostructure assembly
Source: Nucleic Acids Res. 2015 Jun 13;43(13):6587–95. doi: 10.1093/nar/gkv602 (PMC4513873; doi:10.1093/nar/gkv602)
Supplement: SUPPLEMENTARY DATA [file supp_gkv602_nar-00144-f-2015-File008.docx]

**Supplementary notes**

**Supplementary note 1:** List of DNA sequences

**Supplementary note 2:** Effect of fluorophore selection on junction formation

**Supplementary note 3:** Design considerations in junctions K3/K3’

**Supplementary note 4:** Junction labeling in DNA origami shape

**Supplementary note 5:** Gel electrophoresis results for reconstructed rectangle

**Supplementary note 6:** Model design and code

**Supplementary note 1: List of DNA sequences**

**J1 Junction**

| J1_1 | CGCAATCCTGAGCACG |
| --- | --- |
| J1_2 | GCCATAGTGGATTGCG |
| J1_3 | GCATTCGGACTATGGC |
| J1_4 | CGTGCTCACCGAATGC |
| J1_1_FAM | /56FAM/CGCAATCCTGAGCACG |
| J1_2_FAM | /56FAM/GCCATAGTGGATTGCG |
| J1_3_FAM | /56FAM/GCATTCGGACTATGGC |
| J1_4_FAM | /56FAM/CGTGCTCACCGAATGC |
| J1_1_FQ | CGCAATCCTGAGCACG/3IABkFQ/ |
| J1_2_FQ | GCCATAGTGGATTGCG/3IABkFQ/ |
| J1_3_FQ | GCATTCGGACTATGGC/3IABkFQ/ |
| J1_4_FQ | CGTGCTCACCGAATGC/3IABkFQ/ |

Arms alone strands

| J1_a_FAM | /56FAM/CGCAATCC |
| --- | --- |
| J1_b_FAM | /56FAM/CGTGCTCA |
| J1_c_FAM | /56FAM/GCATTCGG |
| J1_d_FAM | /56FAM/GCCATAGT |
| J1_a_FQ | GGATTGCG/3IABkFQ/ |
| J1_b_FQ | TGAGCACG/3IABkFQ/ |
| J1_c_FQ | CCGAATGC/3IABkFQ/ |
| J1_d_FQ | ACTATGGC/3IABkFQ/ |

**K1 Junction**

| K1_1 | /5HEX/GGCAGTAGATGTCGGAATAAAGGCGCTTCTTACGCAATGAGTCTGTTGTGCTATGGT/3IABkFQ/ |
| --- | --- |
| K1_2 | /56FAM/ACCATAGCACAACAGACTCATTGCGTAAGAAGCGGGAGAGGGTA/3IAbRQSp/ |
| K1_3 | /5Cy5/TACCCTCTCCTGACGAGCCGTTA/3IAbRQSp/ |
| K1_4 | /5TEX615/TAACGGCTCGTCACCTTTATTCCGACATCTACTGCC/3IABkFQ/ |

Arms alone strand

| K1_a* | /56FAM/ACCATAGCACAACAGACTCATTGCGTAAGAAGCG |
| --- | --- |
| K1_a | CGCTTCTTACGCAATGAGTCTGTTGTGCTATGGT/3IABkFQ/ |
| K1_b* | /5HEX/GGCAGTAGATGTCGGAATAAAGG |
| K1_b | CCTTTATTCCGACATCTACTGCC/3IABkFQ/ |
| K1_c* | /5TEX615/TAACGGCTCGTCA |
| K1_c | TGACGAGCCGTTA/3IAbRQSp/ |
| K1_d* | /5Cy5/TACCCTCTCC |
| K1_d | GGAGAGGGTA/3IAbRQSp/ |

**K1^FAM^ Junction**

| K1^FAM^_1 | GGCAGTAGATGTCGGAATAAAGGCGCTTCTTACGCAATGAGTCTGTTGTGCTATGGT |
| --- | --- |
| K1^FAM^_1_FAM | /56FAM/GGCAGTAGATGTCGGAATAAAGGCGCTTCTTACGCAATGAGTCTGTTGTGCTATGGT |
| K1^FAM^_1_FQ | GGCAGTAGATGTCGGAATAAAGGCGCTTCTTACGCAATGAGTCTGTTGTGCTATGGT/3IABkFQ/ |
| K1^FAM^_2 | ACCATAGCACAACAGACTCATTGCGTAAGAAGCGGGAGAGGGTA |
| K1^FAM^_2_FAM | /56FAM/ACCATAGCACAACAGACTCATTGCGTAAGAAGCGGGAGAGGGTA |
| K1^FAM^_2_FQ | ACCATAGCACAACAGACTCATTGCGTAAGAAGCGGGAGAGGGTA/3IABkFQ/ |
| K1^FAM^_3 | TACCCTCTCCTGACGAGCCGTTA |
| K1^FAM^_3_FAM | /56FAM/TACCCTCTCCTGACGAGCCGTTA |
| K1^FAM^_3_FQ | TACCCTCTCCTGACGAGCCGTTA/3IABkFQ/ |
| K1^FAM^_4 | TAACGGCTCGTCACCTTTATTCCGACATCTACTGCC |
| K1^FAM^_4_FAM | /56FAM/TAACGGCTCGTCACCTTTATTCCGACATCTACTGCC |
| K1^FAM^_4_FQ | TAACGGCTCGTCACCTTTATTCCGACATCTACTGCC/3IABkFQ/ |

**K2 Junction**

| K2_1 | GGCAGTAGATGTCGGAATAAAGGCGCTTCTTACGCAATGAGTCTGTTGTGCTATGGT |
| --- | --- |
| K2_1_FAM | /56FAM/GGCAGTAGATGTCGGAATAAAGGCGCTTCTTACGCAATGAGTCTGTTGTGCTATGGT |
| K2_1_FQ | GGCAGTAGATGTCGGAATAAAGGCGCTTCTTACGCAATGAGTCTGTTGTGCTATGGT/3IABkFQ/ |
| K2_2 | ACCATAGCACAACAGACTCATTGCGTAAGAAGCGTGACGAGCCGTTA |
| K2_2_FAM | /56FAM/ACCATAGCACAACAGACTCATTGCGTAAGAAGCGTGACGAGCCGTTA |
| K2_2_FQ | ACCATAGCACAACAGACTCATTGCGTAAGAAGCGTGACGAGCCGTTA/3IABkFQ/ |
| K2_3 | TAACGGCTCGTCAGGAGAGGGTA |
| K2_3_FAM | /56FAM/TAACGGCTCGTCAGGAGAGGGTA |
| K2_3_FQ | TAACGGCTCGTCAGGAGAGGGTA/3IABkFQ/ |
| K2_4 | TACCCTCTCCCCTTTATTCCGACATCTACTGCC |
| K2_4_FAM | /56FAM/TACCCTCTCCCCTTTATTCCGACATCTACTGCC |
| K2_4_FQ | TACCCTCTCCCCTTTATTCCGACATCTACTGCC/3IABkFQ/ |

**K3 Junction**

| K3_1 | TAACGGCTCGTCACGCTTCTTACGCAATGAGTCTGTTGTGCTATGGT |
| --- | --- |
| K3_1_FAM | /56FAM/TAACGGCTCGTCACGCTTCTTACGCAATGAGTCTGTTGTGCTATGGT |
| K3_1_FQ | TAACGGCTCGTCACGCTTCTTACGCAATGAGTCTGTTGTGCTATGGT/3IABkFQ/ |
| K3_2 | ACCATAGCACAACAGACTCATTGCGTAAGAAGCGGGAGAGGGTA |
| K3_2_FAM | /56FAM/ACCATAGCACAACAGACTCATTGCGTAAGAAGCGGGAGAGGGTA |
| K3_2_FQ | ACCATAGCACAACAGACTCATTGCGTAAGAAGCGGGAGAGGGTA/3IABkFQ/ |
| K3_3 | TACCCTCTCCCCTTTATTCCGACATCTACTGCC |
| K3_3_FAM | /56FAM/TACCCTCTCCCCTTTATTCCGACATCTACTGCC |
| K3_3_FQ | TACCCTCTCCCCTTTATTCCGACATCTACTGCC/3IABkFQ/ |
| K3_4 | GGCAGTAGATGTCGGAATAAAGGTGACGAGCCGTTA |
| K3_4_FAM | /56FAM/GGCAGTAGATGTCGGAATAAAGGTGACGAGCCGTTA |
| K3_4_FQ | GGCAGTAGATGTCGGAATAAAGGTGACGAGCCGTTA/3IABkFQ/ |

**K3’ Junction**

| K3’_1 | TAACGGCTCGTCACGCTTCTTACGCAATGAGTCTGTTGTGCTATGGT |
| --- | --- |
| K3’_1_FAM | /56FAM/TAACGGCTCGTCACGCTTCTTACGCAATGAGTCTGTTGTGCTATGGT |
| K3’_1_FQ | TAACGGCTCGTCACGCTTCTTACGCAATGAGTCTGTTGTGCTATGGT/3IABkFQ/ |
| K3’_2 | ACCATAGCACAACAGACTCATTGCGTAAGAAGCGGGAGAGGGTA |
| K3’_2_FAM | /56FAM/ACCATAGCACAACAGACTCATTGCGTAAGAAGCGGGAGAGGGTA |
| K3’_2_FQ | ACCATAGCACAACAGACTCATTGCGTAAGAAGCGGGAGAGGGTA/3IABkFQ/ |
| K3’_3 | TACCCTCTCCGCTTTATTCCGACATCTACTGCC |
| K3’_3_FAM | /56FAM/TACCCTCTCCGCTTTATTCCGACATCTACTGCC |
| K3’_3_FQ | TACCCTCTCCGCTTTATTCCGACATCTACTGCC/3IABkFQ/ |
| K3’_4 | GGCAGTAGATGTCGGAATAAAGCTGACGAGCCGTTA |
| K3’_4_FAM | /56FAM/GGCAGTAGATGTCGGAATAAAGCTGACGAGCCGTTA |
| K3’_4_FQ | GGCAGTAGATGTCGGAATAAAGCTGACGAGCCGTTA/3IABkFQ/ |

**K1, K2 and K3 arms alone (without the tail part)**

| K_*a | /56FAM/ACCATAGCACAACAGACTCATTGCGTAAGAAGCG |
| --- | --- |
| K_a | CGCTTCTTACGCAATGAGTCTGTTGTGCTATGGT/3IABkFQ/ |
| K_b* | /56FAM/GGCAGTAGATGTCGGAATAAAGG |
| K_b | CCTTTATTCCGACATCTACTGCC/3IABkFQ/ |
| K_c* | /56FAM/TAACGGCTCGTCA |
| K_c | TGACGAGCCGTTA/3IABkFQ/ |
| K_d* | /56FAM/TACCCTCTCC |
| K_d | GGAGAGGGTA/3IABkFQ/ |

**DNA strands for 2J and 2J’ system experiments**

**2J System**

| 2J_1 | GGCAGTAGATGTCGGAATAAAGGCGCTTCTTACGCAATGAGTCTGTTGTGCTATGGT |
| --- | --- |
| 2J_1_FAM | /56FAM/GGCAGTAGATGTCGGAATAAAGGCGCTTCTTACGCAATGAGTCTGTTGTGCTATGGT |
| 2J_1_FQ | GGCAGTAGATGTCGGAATAAAGGCGCTTCTTACGCAATGAGTCTGTTGTGCTATGGT/3IABkFQ/ |
| 2J_2 | ATACCTGATGATACCCTCTCCTGACGAGCCG |
| 2J_2_FAM | /56FAM/ATACCTGATGATACCCTCTCCTGACGAGCCG |
| 2J_2_FQ | ATACCTGATGATACCCTCTCCTGACGAGCCG/3IABkFQ/ |
| 2J_2_C | ATACCTGATGATACCCTC |
| 2J_2_C_FAM | /56FAM/ATACCTGATGATACCCTC |
| 2J_2_C_FQ | ATACCTGATGATACCCTC/3IABkFQ/ |
| 2J_3 | TTCACTCATCGC |
| 2J_3_FAM | /56FAM/TTCACTCATCGC |
| 2J_3_FQ | TTCACTCATCGC/3IABkFQ/ |
| 2J_4 | CGGCTCGTCAGGACCTTTATTCCGACATCTACTGCC |
| 2J_4_FAM | /56FAM/CGGCTCGTCAGGACCTTTATTCCGACATCTACTGCC |
| 2J_4_FQ | CGGCTCGTCAGGACCTTTATTCCGACATCTACTGCC/3IABkFQ/ |
| 2J_5 | ACCATAGCACAACAGACTCATTGCGTAAGAAGCGGAGGGTATCATCGAA |
| 2J_5_FAM | /56FAM/ACCATAGCACAACAGACTCATTGCGTAAGAAGCGGAGGGTATCATCGAA |
| 2J_5_FQ | ACCATAGCACAACAGACTCATTGCGTAAGAAGCGGAGGGTATCATCGAA/3IABkFQ/ |
| 2J_6 | GCGATGAGTAGGTAT |
| 2J_6_FAM | /56FAM/GCGATGAGTAGGTAT |
| 2J_6_FQ | GCGATGAGTAGGTAT/3IABkFQ/ |

**2J System, S junction**

| 2J_2_sj | ATACCTGATGATACCCTC |
| --- | --- |
| 2J_2_sj_FAM | /56FAM/ATACCTGATGATACCCTC |
| 2J_2_sj_FQ | ATACCTGATGATACCCTC/3IABkFQ/ |
| 2J_5_sj | GAGGGTATCATCGAA |
| 2J_5_sj_FAM | /56FAM/GAGGGTATCATCGAA |
| 2J_5_sj_FQ | GAGGGTATCATCGAA/3IABkFQ/ |

Additional sequences: 2J_3, 2J_3_FAM, 2J_3_FQ

2J_6, 2J_6_FAM, 2J_6_FQ

**2J’ System**

| 2J’_2 | GCGATGAGTGATGATACCCTCTCCTGACGAGCCG |
| --- | --- |
| 2J’_2_FAM | /56FAM/GCGATGAGTGATGATACCCTCTCCTGACGAGCCG |
| 2J’_2_FQ | GCGATGAGTGATGATACCCTCTCCTGACGAGCCG/3IABkFQ/ |
| 2J’_3 | TTCAGGTAT |
| 2J’_3_FAM | /56FAM/TTCAGGTAT |
| 2J’_3_FQ | TTCAGGTAT/3IABkFQ/ |
| 2J’_6 | ATACCTACTCATCGC |
| 2J’_6_FAM | /56FAM/ATACCTACTCATCGC |
| 2J’_6_FQ | ATACCTACTCATCGC/3IABkFQ/ |

Additional sequences: 2J_1, 2J_1_FAM, 2J_1_FQ

2J_4, 2J_4_FAM, 2J_4_FQ

2J_5, 2J_5_FAM, 2J_5_FQ

**DNA strands for 4J system experiments**

Scaffold strands (4J separate system only)

| 12_Scaf_Rec | CAGCCTCTTCCCTATCTCGGGCTAT |
| --- | --- |
| 13_Scaf_Rec | ACGGTACATGGGTTCCTATTGGGCTTGCTATCC |
| 14_Scaf_Rec | CTAACGTCTGGAAAGACGACAAAACTTTAGATCGTTACGCTAACTATGAGGGC |
| 15_Scaf_Rec | CTGATAAACCGATACAATTAAAGGCTCCTTTTGGAGCCTTTTTTTTGGAGATTTTCAACGTGAAAAAATTATTA |
| 16_Scaf_Rec | CCCGCAAAAGCGGCCTTTAACTCCCTGCAAGCCTCAGCGACCGAATAT |

Staple strands

| rec_69 | ATATTCGGTCGCTGAGGCTTGCAGGGAGTTAAAAAAGGCTCCAAAAGGTGTCGTCTTTCCAGACGTTAG |
| --- | --- |
| rec_64 | CCTCAGCATAATAATTTTTTCACGTTGAAAATCTCCAAAAAGGCCGCTTTTGCGGGATTAAACG |
| rec_1 | GGATAGCATAACGATCTAAAGTTTAGCCTTTAATTGTATCGGTTTATCAG |
| rec_2 | CTTGCTGCCCTCATAGTTAGCGAGCCCAATAGGAACCCAAGAGGCTG |
| rec_3 | TGTTCCAGATAGCCCGAGATAGGGATGTACCGT |

Tagged staple strands

| 1R-FQ | ATATTCGGTCGCTGAGGCTTGCAGGGAGTT**A(FQ)**AAAAAGGCTCCAAAAGGTGTCGTCTTTCCAGACGTTAG |
| --- | --- |
| 1R-FAM | ATATTCGGTCGCTGAGGCTTGCAGGGAGTTAAA**A(FAM)**AAGGCTCCAAAAGGTGTCGTCTTTCCAGACGTTAG |
| 1L-FQ | CCTCAGCATAATAATTTTTTCACGTTGAAAATCTCCAAAAA**G(FQ)**GCCGCTTTTGCGGGATTAAACG |
| 1L-FAM | CCTCAGCATAATAATTTTTTCACGTTGAAAATCTCCAA**A(FAM)**AAGGCCGCTTTTGCGGGATTAAACG |
| 2L-FQ | ATATTCGGTCGCTGAGGCTTGCAGGGAGTTAAAAAAGGCTCCAAAA**G(FQ)**GTGTCGTCTTTCCAGACGTTAG |
| 2L-FAM | ATATTCGGTCGCTGAGGCTTGCAGGGAGTTAAAAAAGGCTCCAAAAGGT**G(FAM)**TCGTCTTTCCAGACGTTAG |
| 2R-FQ | GGATAGCATAACGATCTAAAGTTTA**G(FQ)**CCTTTAATTGTATCGGTTTATCAG |
| 2R-FAM | GGATAGCATAACGATCTAAAGT**T(FAM)**TAGCCTTTAATTGTATCGGTTTATCAG |
| 3R-FQ | CTTGCTGCCCTCATAGTTAG**C(FQ)**GAGCCCAATAGGAACCCAAGAGGCTG |
| 3R-FAM | CTTGCTGCCCTCATAGTTAGCGA**G(FAM)**CCCAATAGGAACCCAAGAGGCTG |
| 3L-FAM | GGATAG**C(FAM)**ATAACGATCTAAAGTTTAGCCTTTAATTGTATCGGTTTATCAG |
| 3L-FQ | GGATAGCAT**A(FQ)**ACGATCTAAAGTTTAGCCTTTAATTGTATCGGTTTATCAG |
| 4L-FQ | CTTGCTGCCCTCATAGTTAGCGAGCCCAATAGGAAC**C(FQ)**CAAGAGGCTG |
| 4L-FAM | CTTGCTGCCCTCATAGTTAGCGAGCCCAATAGGAACCCA**A(FAM)**GAGGCTG |
| 4R-FQ | TGTTCCAGATAGCCCGAGATAGGGA**T(FQ)**GTACCGT |
| 4R-FAM | TGTTCCAGATAGCCCGAGATAG**G(FAM)**GATGTACCGT |

Staple strands for “knock out” experiments:

| Rec_01_cut | TAACGATCTAAAGTTTAGCCTTTAATTGTATCGGTTTATCAG |
| --- | --- |
| Rec_69_cut | ATATTCGGTCGCTGAGGCTTGCAGGGAGTTAAAAAAGGCTCCAAAAGG |
| 1R-FQ_cut | ATATTCGGTCGCTGAGGCTTGCAGGGAGTT**A(FQ)**AAAAAGGCTCCAAAAGG |
| 1R-FAM_cut | ATATTCGGTCGCTGAGGCTTGCAGGGAGTTAAA**A(FAM)**AAGGCTCCAAAAGG |
| 2L-FQ_cut | ATATTCGGTCGCTGAGGCTTGCAGGGAGTTAAAAAAGGCTCCAAAA**G(FQ)**G |
| 2R-FQ_cut | TAACGATCTAAAGTTTA**G(FQ)**CCTTTAATTGTATCGGTTTATCAG |
| 2R-FAM_cut | TAACGATCTAAAGT**T(FAM)**TAGCCTTTAATTGTATCGGTTTATCAG |
| 3L-FQ_cut | T**A(FQ)**ACGATCTAAAGTTTAGCCTTTAATTGTATCGGTTTATCA |

**DNA strands for reconstructed rectangle experiments**

Scaffold strand

>M13mp18 [length=7249] [version=09-MAY-2008] [topology=circular] Cloning vector M13mp18, complete sequence.

AATGCTACTACTATTAGTAGAATTGATGCCACCTTTTCAGCTCGCGCCCCAAATGAAAATATAGCTAAACAGGTTATTGACCATTTGCGAAATGTATCTAATGGTCAAACTAAATCTACTCGTTCGCAGAATTGGGAATCAACTGTTATATGGAATGAAACTTCCAGACACCGTACTTTAGTTGCATATTTAAAACATGTTGAGCTACAGCATTATATTCAGCAATTAAGCTCTAAGCCATCCGCAAAAATGACCTCTTATCAAAAGGAGCAATTAAAGGTACTCTCTAATCCTGACCTGTTGGAGTTTGCTTCCGGTCTGGTTCGCTTTGAAGCTCGAATTAAAACGCGATATTTGAAGTCTTTCGGGCTTCCTCTTAATCTTTTTGATGCAATCCGCTTTGCTTCTGACTATAATAGTCAGGGTAAAGACCTGATTTTTGATTTATGGTCATTCTCGTTTTCTGAACTGTTTAAAGCATTTGAGGGGGATTCAATGAATATTTATGACGATTCCGCAGTATTGGACGCTATCCAGTCTAAACATTTTACTATTACCCCCTCTGGCAAAACTTCTTTTGCAAAAGCCTCTCGCTATTTTGGTTTTTATCGTCGTCTGGTAAACGAGGGTTATGATAGTGTTGCTCTTACTATGCCTCGTAATTCCTTTTGGCGTTATGTATCTGCATTAGTTGAATGTGGTATTCCTAAATCTCAACTGATGAATCTTTCTACCTGTAATAATGTTGTTCCGTTAGTTCGTTTTATTAACGTAGATTTTTCTTCCCAACGTCCTGACTGGTATAATGAGCCAGTTCTTAAAATCGCATAAGGTAATTCACAATGATTAAAGTTGAAATTAAACCATCTCAAGCCCAATTTACTACTCGTTCTGGTGTTTCTCGTCAGGGCAAGCCTTATTCACTGAATGAGCAGCTTTGTTACGTTGATTTGGGTAATGAATATCCGGTTCTTGTCAAGATTACTCTTGATGAAGGTCAGCCAGCCTATGCGCCTGGTCTGTACACCGTTCATCTGTCCTCTTTCAAAGTTGGTCAGTTCGGTTCCCTTATGATTGACCGTCTGCGCCTCGTTCCGGCTAAGTAACATGGAGCAGGTCGCGGATTTCGACACAATTTATCAGGCGATGATACAAATCTCCGTTGTACTTTGTTTCGCGCTTGGTATAATCGCTGGGGGTCAAAGATGAGTGTTTTAGTGTATTCTTTTGCCTCTTTCGTTTTAGGTTGGTGCCTTCGTAGTGGCATTACGTATTTTACCCGTTTAATGGAAACTTCCTCATGAAAAAGTCTTTAGTCCTCAAAGCCTCTGTAGCCGTTGCTACCCTCGTTCCGATGCTGTCTTTCGCTGCTGAGGGTGACGATCCCGCAAAAGCGGCCTTTAACTCCCTGCAAGCCTCAGCGACCGAATATATCGGTTATGCGTGGGCGATGGTTGTTGTCATTGTCGGCGCAACTATCGGTATCAAGCTGTTTAAGAAATTCACCTCGAAAGCAAGCTGATAAACCGATACAATTAAAGGCTCCTTTTGGAGCCTTTTTTTTGGAGATTTTCAACGTGAAAAAATTATTATTCGCAATTCCTTTAGTTGTTCCTTTCTATTCTCACTCCGCTGAAACTGTTGAAAGTTGTTTAGCAAAATCCCATACAGAAAATTCATTTACTAACGTCTGGAAAGACGACAAAACTTTAGATCGTTACGCTAACTATGAGGGCTGTCTGTGGAATGCTACAGGCGTTGTAGTTTGTACTGGTGACGAAACTCAGTGTTACGGTACATGGGTTCCTATTGGGCTTGCTATCCCTGAAAATGAGGGTGGTGGCTCTGAGGGTGGCGGTTCTGAGGGTGGCGGTTCTGAGGGTGGCGGTACTAAACCTCCTGAGTACGGTGATACACCTATTCCGGGCTATACTTATATCAACCCTCTCGACGGCACTTATCCGCCTGGTACTGAGCAAAACCCCGCTAATCCTAATCCTTCTCTTGAGGAGTCTCAGCCTCTTAATACTTTCATGTTTCAGAATAATAGGTTCCGAAATAGGCAGGGGGCATTAACTGTTTATACGGGCACTGTTACTCAAGGCACTGACCCCGTTAAAACTTATTACCAGTACACTCCTGTATCATCAAAAGCCATGTATGACGCTTACTGGAACGGTAAATTCAGAGACTGCGCTTTCCATTCTGGCTTTAATGAGGATTTATTTGTTTGTGAATATCAAGGCCAATCGTCTGACCTGCCTCAACCTCCTGTCAATGCTGGCGGCGGCTCTGGTGGTGGTTCTGGTGGCGGCTCTGAGGGTGGTGGCTCTGAGGGTGGCGGTTCTGAGGGTGGCGGCTCTGAGGGAGGCGGTTCCGGTGGTGGCTCTGGTTCCGGTGATTTTGATTATGAAAAGATGGCAAACGCTAATAAGGGGGCTATGACCGAAAATGCCGATGAAAACGCGCTACAGTCTGACGCTAAAGGCAAACTTGATTCTGTCGCTACTGATTACGGTGCTGCTATCGATGGTTTCATTGGTGACGTTTCCGGCCTTGCTAATGGTAATGGTGCTACTGGTGATTTTGCTGGCTCTAATTCCCAAATGGCTCAAGTCGGTGACGGTGATAATTCACCTTTAATGAATAATTTCCGTCAATATTTACCTTCCCTCCCTCAATCGGTTGAATGTCGCCCTTTTGTCTTTGGCGCTGGTAAACCATATGAATTTTCTATTGATTGTGACAAAATAAACTTATTCCGTGGTGTCTTTGCGTTTCTTTTATATGTTGCCACCTTTATGTATGTATTTTCTACGTTTGCTAACATACTGCGTAATAAGGAGTCTTAATCATGCCAGTTCTTTTGGGTATTCCGTTATTATTGCGTTTCCTCGGTTTCCTTCTGGTAACTTTGTTCGGCTATCTGCTTACTTTTCTTAAAAAGGGCTTCGGTAAGATAGCTATTGCTATTTCATTGTTTCTTGCTCTTATTATTGGGCTTAACTCAATTCTTGTGGGTTATCTCTCTGATATTAGCGCTCAATTACCCTCTGACTTTGTTCAGGGTGTTCAGTTAATTCTCCCGTCTAATGCGCTTCCCTGTTTTTATGTTATTCTCTCTGTAAAGGCTGCTATTTTCATTTTTGACGTTAAACAAAAAATCGTTTCTTATTTGGATTGGGATAAATAATATGGCTGTTTATTTTGTAACTGGCAAATTAGGCTCTGGAAAGACGCTCGTTAGCGTTGGTAAGATTCAGGATAAAATTGTAGCTGGGTGCAAAATAGCAACTAATCTTGATTTAAGGCTTCAAAACCTCCCGCAAGTCGGGAGGTTCGCTAAAACGCCTCGCGTTCTTAGAATACCGGATAAGCCTTCTATATCTGATTTGCTTGCTATTGGGCGCGGTAATGATTCCTACGATGAAAATAAAAACGGCTTGCTTGTTCTCGATGAGTGCGGTACTTGGTTTAATACCCGTTCTTGGAATGATAAGGAAAGACAGCCGATTATTGATTGGTTTCTACATGCTCGTAAATTAGGATGGGATATTATTTTTCTTGTTCAGGACTTATCTATTGTTGATAAACAGGCGCGTTCTGCATTAGCTGAACATGTTGTTTATTGTCGTCGTCTGGACAGAATTACTTTACCTTTTGTCGGTACTTTATATTCTCTTATTACTGGCTCGAAAATGCCTCTGCCTAAATTACATGTTGGCGTTGTTAAATATGGCGATTCTCAATTAAGCCCTACTGTTGAGCGTTGGCTTTATACTGGTAAGAATTTGTATAACGCATATGATACTAAACAGGCTTTTTCTAGTAATTATGATTCCGGTGTTTATTCTTATTTAACGCCTTATTTATCACACGGTCGGTATTTCAAACCATTAAATTTAGGTCAGAAGATGAAATTAACTAAAATATATTTGAAAAAGTTTTCTCGCGTTCTTTGTCTTGCGATTGGATTTGCATCAGCATTTACATATAGTTATATAACCCAACCTAAGCCGGAGGTTAAAAAGGTAGTCTCTCAGACCTATGATTTTGATAAATTCACTATTGACTCTTCTCAGCGTCTTAATCTAAGCTATCGCTATGTTTTCAAGGATTCTAAGGGAAAATTAATTAATAGCGACGATTTACAGAAGCAAGGTTATTCACTCACATATATTGATTTATGTACTGTTTCCATTAAAAAAGGTAATTCAAATGAAATTGTTAAATGTAATTAATTTTGTTTTCTTGATGTTTGTTTCATCATCTTCTTTTGCTCAGGTAATTGAAATGAATAATTCGCCTCTGCGCGATTTTGTAACTTGGTATTCAAAGCAATCAGGCGAATCCGTTATTGTTTCTCCCGATGTAAAAGGTACTGTTACTGTATATTCATCTGACGTTAAACCTGAAAATCTACGCAATTTCTTTATTTCTGTTTTACGTGCAAATAATTTTGATATGGTAGGTTCTAACCCTTCCATTATTCAGAAGTATAATCCAAACAATCAGGATTATATTGATGAATTGCCATCATCTGATAATCAGGAATATGATGATAATTCCGCTCCTTCTGGTGGTTTCTTTGTTCCGCAAAATGATAATGTTACTCAAACTTTTAAAATTAATAACGTTCGGGCAAAGGATTTAATACGAGTTGTCGAATTGTTTGTAAAGTCTAATACTTCTAAATCCTCAAATGTATTATCTATTGACGGCTCTAATCTATTAGTTGTTAGTGCTCCTAAAGATATTTTAGATAACCTTCCTCAATTCCTTTCAACTGTTGATTTGCCAACTGACCAGATATTGATTGAGGGTTTGATATTTGAGGTTCAGCAAGGTGATGCTTTAGATTTTTCATTTGCTGCTGGCTCTCAGCGTGGCACTGTTGCAGGCGGTGTTAATACTGACCGCCTCACCTCTGTTTTATCTTCTGCTGGTGGTTCGTTCGGTATTTTTAATGGCGATGTTTTAGGGCTATCAGTTCGCGCATTAAAGACTAATAGCCATTCAAAAATATTGTCTGTGCCACGTATTCTTACGCTTTCAGGTCAGAAGGGTTCTATCTCTGTTGGCCAGAATGTCCCTTTTATTACTGGTCGTGTGACTGGTGAATCTGCCAATGTAAATAATCCATTTCAGACGATTGAGCGTCAAAATGTAGGTATTTCCATGAGCGTTTTTCCTGTTGCAATGGCTGGCGGTAATATTGTTCTGGATATTACCAGCAAGGCCGATAGTTTGAGTTCTTCTACTCAGGCAAGTGATGTTATTACTAATCAAAGAAGTATTGCTACAACGGTTAATTTGCGTGATGGACAGACTCTTTTACTCGGTGGCCTCACTGATTATAAAAACACTTCTCAGGATTCTGGCGTACCGTTCCTGTCTAAAATCCCTTTAATCGGCCTCCTGTTTAGCTCCCGCTCTGATTCTAACGAGGAAAGCACGTTATACGTGCTCGTCAAAGCAACCATAGTACGCGCCCTGTAGCGGCGCATTAAGCGCGGCGGGTGTGGTGGTTACGCGCAGCGTGACCGCTACACTTGCCAGCGCCCTAGCGCCCGCTCCTTTCGCTTTCTTCCCTTCCTTTCTCGCCACGTTCGCCGGCTTTCCCCGTCAAGCTCTAAATCGGGGGCTCCCTTTAGGGTTCCGATTTAGTGCTTTACGGCACCTCGACCCCAAAAAACTTGATTTGGGTGATGGTTCACGTAGTGGGCCATCGCCCTGATAGACGGTTTTTCGCCCTTTGACGTTGGAGTCCACGTTCTTTAATAGTGGACTCTTGTTCCAAACTGGAACAACACTCAACCCTATCTCGGGCTATTCTTTTGATTTATAAGGGATTTTGCCGATTTCGGAACCACCATCAAACAGGATTTTCGCCTGCTGGGGCAAACCAGCGTGGACCGCTTGCTGCAACTCTCTCAGGGCCAGGCGGTGAAGGGCAATCAGCTGTTGCCCGTCTCACTGGTGAAAAGAAAAACCACCCTGGCGCCCAATACGCAAACCGCCTCTCCCCGCGCGTTGGCCGATTCATTAATGCAGCTGGCACGACAGGTTTCCCGACTGGAAAGCGGGCAGTGAGCGCAACGCAATTAATGTGAGTTAGCTCACTCATTAGGCACCCCAGGCTTTACACTTTATGCTTCCGGCTCGTATGTTGTGTGGAATTGTGAGCGGATAACAATTTCACACAGGAAACAGCTATGACCATGATTACGAATTCGAGCTCGGTACCCGGGGATCCTCTAGAGTCGACCTGCAGGCATGCAAGCTTGGCACTGGCCGTCGTTTTACAACGTCGTGACTGGGAAAACCCTGGCGTTACCCAACTTAATCGCCTTGCAGCACATCCCCCTTTCGCCAGCTGGCGTAATAGCGAAGAGGCCCGCACCGATCGCCCTTCCCAACAGTTGCGCAGCCTGAATGGCGAATGGCGCTTTGCCTGGTTTCCGGCACCAGAAGCGGTGCCGGAAAGCTGGCTGGAGTGCGATCTTCCTGAGGCCGATACTGTCGTCGTCCCCTCAAACTGGCAGATGCACGGTTACGATGCGCCCATCTACACCAACGTGACCTATCCCATTACGGTCAATCCGCCGTTTGTTCCCACGGAGAATCCGACGGGTTGTTACTCGCTCACATTTAATGTTGATGAAAGCTGGCTACAGGAAGGCCAGACGCGAATTATTTTTGATGGCGTTCCTATTGGTTAAAAAATGAGCTGATTTAACAAAAATTTAATGCGAATTTTAACAAAATATTAACGTTTACAATTTAAATATTTGCTTATACAATCTTCCTGTTTTTGGGGCTTTTCTGATTATCAACCGGGGTACATATGATTGACATGCTAGTTTTACGATTACCGTTCATCGATTCTCTTGTTTGCTCCAGACTCTCAGGCAATGACCTGATAGCCTTTGTAGATCTCTCAAAAATAGCTACCCTCTCCGGCATTAATTTATCAGCTAGAACGGTTGAATATCATATTGATGGTGATTTGACTGTCTCCGGCCTTTCTCACCCTTTTGAATCTTTACCTACACATTACTCAGGCATTGCATTTAAAATATATGAGGGTTCTAAAAATTTTTATCCTTGCGTTGAAATAAAGGCTTCTCCCGCAAAAGTATTACAGGGTCATAATGTTTTTGGTACAACCGATTTAGCTTTATGCTCTGAGGCTTTATTGCTTAATTTTGCTAATTCTTTGCCTTGCCTGTATGATTTATTGGATGTT

Staple strands

| Seq_Name | Start pos | End pos | Sequence |
| --- | --- | --- | --- |
| rec_69 | 16[135] | 14[99] | ATATTCGGTCGCTGAGGCTTGCAGGGAGTTAAAAAAGGCTCCAAAAGGTGTCGTCTTTCCAGACGTTAG |
| rec_64 | 16[79] | 17[95] | CCTCAGCATAATAATTTTTTCACGTTGAAAATCTCCAAAAAGGCCGCTTTTGCGGGATTAAACG |
| rec_1 | 13[128] | 15[145] | GGATAGCATAACGATCTAAAGTTTAGCCTTTAATTGTATCGGTTTATCAG |
| rec_2 | 15[146] | 12[143] | CTTGCTGCCCTCATAGTTAGCGAGCCCAATAGGAACCCAAGAGGCTG |
| rec_3 | 11[160] | 13[160] | TGTTCCAGATAGCCCGAGATAGGGATGTACCGT |
| rec_4 | 17[144] | 16[136] | GGCAAAAGCCCACGCATAACCGAT |
| rec_5 | 14[98] | 14[80] | TAAATGAATTTTCTGTATG |
| rec_6 | 12[176] | 11[176] | ATCAAAAGATTTGGAACA |
| rec_7 | 14[207] | 13[207] | CCGGAAGCGAGCTAAC |
| rec_8 | 11[112] | 10[112] | GCCTATTTATTAAAGC |
| rec_9 | 14[287] | 15[287] | GAGCTCGAAGGGTTTT |
| rec_10 | 17[272] | 16[272] | TTCTCCGTAGTATCGG |
| rec_11 | 10[239] | 9[239] | ATATTACCCACACGAC |
| rec_12 | 13[256] | 14[256] | TGTCGTGCATAGCTGT |
| rec_13 | 22[159] | 23[159] | GAGAGGCTCTTCAAAT |
| rec_14 | 1[208] | 0[208] | CTGATGCATAATTGAG |
| rec_15 | 22[63] | 23[63] | TTGAATCCGAATGACC |
| rec_16 | 14[175] | 13[175] | AACGCCTGAGTTTCGT |
| rec_17 | 23[144] | 22[144] | CCGAAAGATTTGCAAA |
| rec_18 | 20[255] | 21[255] | ATGCAATGAGGCAAAG |
| rec_19 | 20[239] | 19[239] | ATGTGTAGTAGCTATT |
| rec_20 | 18[287] | 19[287] | CAAAAACAGTCTGGAG |
| rec_21 | 4[207] | 3[207] | AAAATCGCGCAAAAGA |
| rec_22 | 10[255] | 11[255] | GGTAATATACGCCAGA |
| rec_23 | 23[176] | 23[191] | AGCTTCAAAGCGAACC |
| rec_24 | 11[192] | 12[192] | AATCAGAGGAAATCGG |
| rec_25 | 4[127] | 5[127] | CGGGAGAAGAACAAAG |
| rec_26 | 22[95] | 23[95] | GTCCAATACCTGACTA |
| rec_27 | 10[303] | 9[303] | AATAACATGTAAGAAT |
| rec_28 | 18[255] | 19[255] | AAATTGTAATCTACAA |
| rec_29 | 0[207] | 0[192] | AATCGCCATATTTAAC |
| rec_30 | 3[192] | 4[192] | TTACCTGAGCAGAGGC |
| rec_31 | 19[272] | 18[272] | GCCTGAGAGGAAGATT |
| rec_32 | 9[176] | 9[191] | GCCGCCAGACGCTCAA |
| rec_33 | 17[176] | 17[191] | ACCCCCAGAGGAACGC |
| rec_34 | 18[191] | 18[176] | TAACCAATCGATTATA |
| rec_35 | 6[143] | 5[143] | AGAAAATAAGGAAACC |
| rec_36 | 21[192] | 22[192] | GTGGCATCTCATTTGG |
| rec_37 | 4[255] | 5[255] | GGGAGAAATAATCCTG |
| rec_38 | 9[208] | 8[208] | ATTTACATCCGCCTGC |
| rec_39 | 16[175] | 15[175] | CGCCGACATAAACAGC |
| rec_40 | 8[143] | 7[143] | GATAGCAGCTTGAGCC |
| rec_41 | 7[144] | 6[144] | ATTTGGGAGCAAACGT |
| rec_42 | 9[272] | 8[272] | ATAGAACCATTAAAAA |
| rec_43 | 19[160] | 20[160] | TTCAGTGAGAGTAGTA |
| rec_44 | 13[288] | 14[288] | CGCGCGGGCGGGTACC |
| rec_45 | 4[191] | 4[176] | GAATTATTATGAAAAT |
| rec_46 | 10[143] | 9[143] | ATATTCACCACCACCC |
| rec_47 | 9[64] | 10[64] | CACCGGAAAGCGTCAT |
| rec_48 | 2[287] | 3[287] | GAAAACATTGGAAACA |
| rec_49 | 11[240] | 10[240] | GGAACGGTCCAGAACA |
| rec_50 | 19[192] | 20[192] | TATTCAACACAGTCAA |
| rec_51 | 10[287] | 11[287] | TGAGTAGAAGTGAGGC |
| rec_52 | 21[128] | 22[128] | CCAAAAGGTGCCAGAG |
| rec_53 | 3[240] | 2[240] | TAATTACATCAATAGT |
| rec_54 | 8[159] | 9[159] | CACCAATGGCCACCAG |
| rec_55 | 9[160] | 10[160] | AACCACCAAGGCAGGT |
| rec_56 | 17[192] | 18[192] | CATCAAAATCATTTTT |
| rec_57 | 21[240] | 20[240] | TACAGGCACCTGAGTA |
| rec_58 | 23[64] | 23[79] | ATAAATCAAAAATCAG |
| rec_59 | 0[143] | 0[128] | AGAATATAAAGTACCG |
| rec_60 | 14[239] | 13[239] | TTATCCGCTGCCCGCT |
| rec_61 | 3[96] | 4[96] | CCAGAGCCAACAAAGT |
| rec_62 | 8[63] | 9[63] | CGGTCATAATCAAAAT |
| rec_63 | 0[271] | 0[256] | TCATATGCGTTATACA |
| rec_64 | 22[127] | 23[127] | GGGGTAATGCATCAAA |
| rec_65 | 0[127] | 1[127] | ACAAAAGGTCATTCCA |
| rec_66 | 3[160] | 4[160] | ACGATTTTTTACAGAG |
| rec_67 | 22[79] | 21[79] | TCGTCATAAGATTCAT |
| rec_68 | 15[160] | 16[160] | GAATTTCTATGACAAC |
| rec_69 | 8[111] | 7[111] | CAAGTTTGATTCATTA |
| rec_70 | 8[271] | 7[271] | TACCGAACCAACAGTT |
| rec_71 | 1[160] | 2[160] | CGAGAACAATTACCGC |
| rec_72 | 22[239] | 21[239] | TACATTTCATAAATCA |
| rec_73 | 7[96] | 8[96] | CGGAAATTCCTTTAGC |
| rec_74 | 2[111] | 1[111] | GAACGCGACGGCTGTC |
| rec_75 | 14[223] | 15[223] | CCACACAACTTCGCTA |
| rec_76 | 2[127] | 3[127] | TTATCCGGAAATAAAC |
| rec_77 | 21[80] | 20[80] | CAGTTGAGCATTATAC |
| rec_78 | 4[303] | 3[303] | TTCAGGTTATCAATAT |
| rec_79 | 3[80] | 2[80] | GCTAACGATCCCGACT |
| rec_80 | 15[272] | 14[272] | GTAACGCCATTCGTAA |
| rec_81 | 1[192] | 2[192] | TAACTATAACCTCCGG |
| rec_82 | 16[271] | 15[271] | CCTCAGGATAAGTTGG |
| rec_83 | 2[79] | 1[79] | TGCGGGAGAATTTACG |
| rec_84 | 10[79] | 9[79] | TTCCAGTACCAGAGCC |
| rec_85 | 8[191] | 8[176] | GAGCCAGCTACCATTA |
| rec_86 | 3[112] | 2[112] | CAGTTACATATTCTAA |
| rec_87 | 0[223] | 1[223] | GTAGGGCTAATCCAAT |
| rec_88 | 2[207] | 1[207] | CCTTTTTATGTAAATG |
| rec_89 | 20[191] | 20[176] | ATCACCATCGAGAAAC |
| rec_90 | 4[159] | 5[159] | AGAATAACGCAATAAT |
| rec_91 | 13[112] | 12[112] | CCACCCTCGATTAGCG |
| rec_92 | 16[303] | 15[303] | CTGCCAGTCGACGTTG |
| rec_93 | 23[288] | 23[303] | TGCTGAATATAATGCT |
| rec_94 | 13[240] | 12[240] | TTCCAGTCCACGCTGG |
| rec_95 | 10[127] | 11[127] | AAATCCTCCGGAACCT |
| rec_96 | 19[288] | 20[288] | CAAACAAGACGCAAGG |
| rec_97 | 7[64] | 8[64] | CAACCGATGGCATTTT |
| rec_98 | 7[224] | 8[224] | AATATCAAGCGGTCAG |
| rec_99 | 10[175] | 9[175] | GGAGGTTGCCAGAGCC |
| rec_100 | 5[304] | 4[304] | ATCAAAATCGTAGATT |
| rec_101 | 20[223] | 21[223] | TCAAAAGGGTAGCATT |
| rec_102 | 17[80] | 16[80] | AAGTTTCCATCGTCAC |
| rec_103 | 10[207] | 9[207] | GCTCATGGAATGGATT |
| rec_104 | 22[175] | 21[175] | TAAAAACCTAACCCTC |
| rec_105 | 12[79] | 11[79] | GTCGAGAGCCTTGAGT |
| rec_106 | 0[63] | 1[63] | GCGCCTGTAATAATAT |
| rec_107 | 20[159] | 21[159] | AATTGGGCAAGAGCAA |
| rec_108 | 8[79] | 7[79] | TTTTCATCTGAGGGAG |
| rec_109 | 18[239] | 17[239] | TATTTTGTTTCATCAA |
| rec_110 | 7[272] | 6[272] | GAAAGGAACTTTACAA |
| rec_111 | 1[64] | 2[64] | CCCATCCTGTTTTGAA |
| rec_112 | 3[176] | 3[191] | CGTCAAAACATTTCAA |
| rec_113 | 17[160] | 18[160] | AAAACACTGAAACAAA |
| rec_114 | 1[288] | 2[288] | AAATTTAAGAATCCTT |
| rec_115 | 15[192] | 16[192] | GGGAAGGGCGCCATTC |
| rec_116 | 11[128] | 12[128] | ATTATTCTTCAAGAGA |
| rec_117 | 2[255] | 3[255] | GAGAAGAGTTTAACAA |
| rec_118 | 1[112] | 0[112] | TTTCCTTATAAAGTAA |
| rec_119 | 3[128] | 4[128] | AGCCATATGCATTAGA |
| rec_120 | 12[95] | 13[95] | CAGGCGGATCAGAACC |
| rec_121 | 4[79] | 3[79] | GCTAATATTTACCAAC |
| rec_122 | 1[240] | 0[240] | CGAGAAAACCAGTATA |
| rec_123 | 16[223] | 17[223] | CCGGAAACCTTCCTGT |
| rec_124 | 13[192] | 14[192] | TAATGAGTATAAAGTG |
| rec_125 | 2[63] | 3[63] | GCCTTAAAAATTTTAT |
| rec_126 | 19[80] | 18[80] | AGGCTGGCGGAACGAG |
| rec_127 | 12[287] | 13[287] | CCTTCACCTCGGCCAA |
| rec_128 | 11[288] | 12[288] | CACCGAGTCTGATTGC |
| rec_129 | 6[111] | 5[111] | AAAGAAACAAGTAAGC |
| rec_130 | 0[159] | 1[159] | TCGAGCCAGCACTCAT |
| rec_131 | 6[223] | 7[223] | TAAAAGTTGAACCTCA |
| rec_132 | 8[239] | 7[239] | GAGGTGAGACCCTCAA |
| rec_133 | 13[96] | 13[111] | GCCACCCTCAGAGCCA |
| rec_134 | 13[208] | 12[208] | TCACATTAGTTTGATG |
| rec_135 | 4[143] | 3[143] | AGGGAAGCTATTTATC |
| rec_136 | 6[63] | 7[63] | TAGAAAATGCGACATT |
| rec_137 | 6[255] | 7[255] | TATTAAATTGGTCAGT |
| rec_138 | 17[288] | 18[288] | ACGGCGGAAAAAGCCC |
| rec_139 | 0[111] | 0[96] | TTCTGTCCAGACGACG |
| rec_140 | 9[144] | 8[144] | TCAGAGCCAAACCATC |
| rec_141 | 9[240] | 8[240] | CAGTAATAATAAAACA |
| rec_142 | 18[159] | 19[159] | GTACAACGGCTGCTCA |
| rec_143 | 23[208] | 22[208] | CCAACAGGCTGTTTAG |
| rec_144 | 19[128] | 20[128] | CATTACCCTCAACTTT |
| rec_145 | 15[240] | 14[240] | AAGGGGGATGAAATTG |
| rec_146 | 17[224] | 18[224] | AGCCAGCTTAAAATTC |
| rec_147 | 5[208] | 4[208] | ATCATATTCAAGTTAC |
| rec_148 | 3[144] | 2[144] | CCAATCCAGCAAGCAA |
| rec_149 | 23[112] | 22[112] | AGCGGATTAGTAAAAT |
| rec_150 | 21[144] | 20[144] | GGCATAGTTTGAGATG |
| rec_151 | 12[223] | 13[223] | AAAATCCTATTGCGTT |
| rec_152 | 11[304] | 10[304] | CTGTCCATTGATTAGT |
| rec_153 | 8[127] | 9[127] | TCAGTAGCCCGCCACC |
| rec_154 | 19[112] | 18[112] | ACAAGAACGAAATCCG |
| rec_155 | 3[256] | 4[256] | TTTCATTTTTTACATC |
| rec_156 | 8[95] | 9[95] | GTCAGACTAACCGCCT |
| rec_157 | 19[304] | 18[304] | TGAACGGTCCCGGTTG |
| rec_158 | 20[271] | 19[271] | CCCTCATAAGGTCATT |
| rec_159 | 5[192] | 6[192] | AAGGAGCGTTTGCGGA |
| rec_160 | 0[303] | 0[288] | AATCATAATTACTAGA |
| rec_161 | 19[176] | 19[191] | TGCCCTGACAATATGA |
| rec_162 | 17[304] | 16[304] | AATGGGATCCGTGCAT |
| rec_163 | 12[207] | 11[207] | GTGGTTCCCGGGAGCT |
| rec_164 | 18[303] | 17[303] | ATAATCAGTTGACCGT |
| rec_165 | 23[96] | 23[111] | TTATAGTCAGAAGCAA |
| rec_166 | 7[112] | 6[112] | AAGGTGAAAACATATA |
| rec_167 | 2[143] | 1[143] | ATCAGATATATTAAAC |
| rec_168 | 20[143] | 19[143] | GTTTAATTAAATCAAC |
| rec_169 | 18[143] | 17[143] | TATCATCGACGAAAGA |
| rec_170 | 11[256] | 12[256] | ATCCTGAGGTTGCAGC |
| rec_171 | 13[224] | 14[224] | GCGCTCACTCACAATT |
| rec_172 | 9[128] | 10[128] | CTCAGAGCAAACAAAT |
| rec_173 | 20[303] | 19[303] | TTATTTCAAGAATCGA |
| rec_174 | 0[255] | 1[255] | AATTCTTACTTTTTCA |
| rec_175 | 2[95] | 3[95] | AGCGAACCGCGTCTTT |
| rec_176 | 15[288] | 16[288] | CCCAGTCATTGAGGGG |
| rec_177 | 11[208] | 10[208] | AAACAGGAGGAAAAAC |
| rec_178 | 9[224] | 10[224] | TCACCAGTGCCAGCCA |
| rec_179 | 18[79] | 17[79] | GCGCAGACTCATGAGG |
| rec_180 | 23[272] | 22[272] | TGGCTTAGCCCAATTC |
| rec_181 | 19[256] | 20[256] | AGGCTATCTATTTTAA |
| rec_182 | 18[207] | 17[207] | AAATCAGCATAATTCG |
| rec_183 | 3[208] | 2[208] | AGATGATGAGAGACTA |
| rec_184 | 2[159] | 3[159] | GCCCAATAAATAAGAA |
| rec_185 | 0[95] | 1[95] | ACAATAAAGAAACCAA |
| rec_186 | 14[303] | 13[303] | AGGATCCCGAGAGGCG |
| rec_187 | 17[256] | 18[256] | GTAACAACAAATATTT |
| rec_188 | 20[79] | 19[79] | CAGTCAGGAGGCGCAT |
| rec_189 | 9[304] | 8[304] | ACGTGGCAAATGCGCG |
| rec_190 | 6[159] | 7[159] | GTATGTTAATTAGAGC |
| rec_191 | 19[208] | 18[208] | CTGATAAATTTTTGTT |
| rec_192 | 6[239] | 5[239] | CGAACGTTTGGCAATT |
| rec_193 | 1[176] | 1[191] | GTTTTTATGGGTTATA |
| rec_194 | 5[64] | 6[64] | GAAATAGCACAATCAA |
| rec_195 | 20[207] | 19[207] | GGCCGGAGCGTTCTAG |
| rec_196 | 7[192] | 8[192] | AAAAATCTCACGCTGA |
| rec_197 | 14[63] | 15[63] | CTTTCAACCTAAAGGA |
| rec_198 | 2[175] | 1[175] | TAGGAATCAGCAAGCC |
| rec_199 | 6[191] | 6[176] | ACAAAGAAATGATTAA |
| rec_200 | 21[304] | 20[304] | TTGTACCAAGAAGCCT |
| rec_201 | 23[240] | 22[240] | TGCTCCTTCCATTAGA |
| rec_202 | 16[287] | 17[287] | ACGACGACGGGAACAA |
| rec_203 | 7[240] | 6[240] | TCAATATCCCTTTGCC |
| rec_204 | 21[224] | 22[224] | AACATCCAGCAAATGG |
| rec_205 | 10[95] | 11[95] | GTCTCTGACCGTATAA |
| rec_206 | 17[64] | 18[64] | AGACTTTTGGTCAATC |
| rec_207 | 18[223] | 19[223] | GCATTAAATTAATGCC |
| rec_208 | 8[207] | 7[207] | AACAGTGCAAAGCATC |
| rec_209 | 7[80] | 6[80] | GGAAGGTAATAAGTTT |
| rec_210 | 19[240] | 18[240] | TTTGAGAGAACGTTAA |
| rec_211 | 12[63] | 13[63] | TAAGTATAGTTTAGTA |
| rec_212 | 2[223] | 3[223] | TAGGTCTGAAACAAAC |
| rec_213 | 10[63] | 11[63] | ACATGGCTTTTAACGG |
| rec_214 | 5[272] | 4[272] | TTCTGAATTACAGTAA |
| rec_215 | 2[239] | 1[239] | GAATTTATAAAGAACG |
| rec_216 | 11[64] | 12[64] | GGTCAGTGGGTTGATA |
| rec_217 | 17[208] | 16[208] | CGTCTGGCCAGGCAAA |
| rec_218 | 4[63] | 5[63] | TAACCCACGAAACAAT |
| rec_219 | 10[159] | 11[159] | CAGACGATTTGAGTGT |
| rec_220 | 13[80] | 12[80] | CGCCACCCTAAGTGCC |
| rec_221 | 12[127] | 13[127] | AGGATTAGATTTTCAG |
| rec_222 | 5[224] | 6[224] | TCAGATGAATTAATTT |
| rec_223 | 16[207] | 15[207] | GCGCCATTCGATCGGT |
| rec_224 | 8[175] | 7[175] | GCAAGGCCTCACCAGT |
| rec_225 | 12[239] | 11[239] | TTTGCCCCTTTAGACA |
| rec_226 | 7[208] | 6[208] | ACCTTGCTTGAGTAAC |
| rec_227 | 12[255] | 13[255] | AAGCGGTCGGGAAACC |
| rec_228 | 17[128] | 18[128] | AACCTAAACCTGATAA |
| rec_229 | 9[192] | 10[192] | TCGTCTGAAAATACCT |
| rec_230 | 0[239] | 0[224] | AAGCCAACGCTCAACA |
| rec_231 | 23[160] | 23[175] | ATCGCGTTTTAATTCG |
| rec_232 | 6[95] | 7[95] | ACCACGGAAATATTGA |
| rec_233 | 8[303] | 7[303] | AACTGATAAAAATATC |
| rec_234 | 23[304] | 22[304] | GTAGCTCATCTGGAAG |
| rec_235 | 1[256] | 2[256] | AATATATTAAGACGCT |
| rec_236 | 7[160] | 8[160] | CAGCAAAAGGAAACGT |
| rec_237 | 4[111] | 3[111] | ACACCCTGTAATTTGC |
| rec_238 | 9[288] | 10[288] | CTGAAAGCCACTTGCC |
| rec_239 | 9[80] | 8[80] | ACCACCGGGTAGCGCG |
| rec_240 | 19[64] | 20[64] | TACAGACCACGTTGGG |
| rec_241 | 16[63] | 17[63] | CAGCATCGAGGACTAA |
| rec_242 | 10[223] | 11[223] | TTGCAACAGGCCGATT |
| rec_243 | 21[160] | 22[160] | CACTATCAAAAATAGC |
| rec_244 | 14[271] | 13[271] | TCATGGTCCAGCTGCA |
| rec_245 | 21[96] | 22[96] | ATACCACATGGATAGC |
| rec_246 | 7[304] | 6[304] | TTTAGGAGGATAATAC |
| rec_247 | 8[255] | 9[255] | AGCAGAAGAAAGGGAC |
| rec_248 | 21[256] | 22[256] | AATTAGCAAGTAGATT |
| rec_249 | 7[288] | 8[288] | GGTTATCTGCCCTAAA |
| rec_250 | 2[303] | 1[303] | TTCCCTTATGGTTTGA |
| rec_251 | 6[287] | 7[287] | GTATTAGATTGAGGAA |
| rec_252 | 21[64] | 22[64] | AGGTAGAAAATATTCA |
| rec_253 | 18[111] | 17[111] | CGACCTGCACGTAATG |
| rec_254 | 21[208] | 20[208] | TAATAGTAGTGAGAAA |
| rec_255 | 1[304] | 0[304] | AATACCGAAACACCGG |
| rec_256 | 17[96] | 18[96] | GGTAAAATTCCATGTT |
| rec_257 | 11[144] | 10[144] | AAAGTATTTGGCCTTG |
| rec_258 | 3[304] | 2[304] | ATGTGAGTAATTAATT |
| rec_259 | 6[79] | 5[79] | ATTTTGTCAATAGCTA |
| rec_260 | 12[111] | 11[111] | GGGTTTTGTGCCCCCT |
| rec_261 | 22[143] | 21[143] | AGAAGTTTAATTACGA |
| rec_262 | 16[239] | 15[239] | GGCACCGCGCTGGCGA |
| rec_263 | 22[207] | 21[207] | CTATATTTAATTCTAC |
| rec_264 | 21[176] | 21[191] | GTTTACCACTGAAAAG |
| rec_265 | 23[80] | 22[80] | GTCTTTACCTGCGGAA |
| rec_266 | 11[96] | 12[96] | ACAGTTAACTCAGTAC |
| rec_267 | 17[240] | 16[240] | CATTAAATAGCTTTCC |
| rec_268 | 18[63] | 19[63] | ATAAGGGAGAACGGTG |
| rec_269 | 9[112] | 8[112] | CCTCAGAAGACAGAAT |
| rec_270 | 3[272] | 2[272] | TTTTTTAAAGCGATAG |
| rec_271 | 21[288] | 22[288] | CATAAAGCCATATAAC |
| rec_272 | 16[191] | 16[176] | AGGCTGCGGATAGTTG |
| rec_273 | 11[80] | 10[80] | AACAGTGCATTTACCG |
| rec_274 | 23[192] | 23[207] | AGACCGGAAGCAAACT |
| rec_275 | 5[160] | 6[160] | AACGGAATATTACGCA |
| rec_276 | 13[272] | 12[272] | TTAATGAAGCCTGGCC |
| rec_277 | 6[271] | 5[271] | ACAATTCGGATTATAC |
| rec_278 | 6[127] | 7[127] | AAGGTGGCTTATCACC |
| rec_279 | 20[63] | 21[63] | AAGAAAAAATTATTAC |
| rec_280 | 20[175] | 19[175] | ACCAGAACATAAGGCT |
| rec_281 | 18[127] | 19[127] | ATTGTGTCCGGATATT |
| rec_282 | 4[239] | 3[239] | GATTCGCCAACAAAAT |
| rec_283 | 22[223] | 23[223] | TCAATAACTCAGGATT |
| rec_284 | 5[112] | 4[112] | AGATAGCCTTAACTGA |
| rec_285 | 20[287] | 21[287] | ATAAAAATCCTCAGAG |
| rec_286 | 4[175] | 3[175] | AGCAGCCTTTGTTTAA |
| rec_287 | 7[128] | 8[128] | GTCACCGACACCGTAA |
| rec_288 | 14[159] | 15[159] | CACAGACATTCGAGGT |
| rec_289 | 9[256] | 10[256] | ATTCTGGCGCCTTGCT |
| rec_290 | 23[256] | 23[271] | AGGTCATTTTTGCGGA |
| rec_291 | 19[144] | 18[144] | GTAACAAAGAGATTTG |
| rec_292 | 20[127] | 21[127] | AATCATTGACATAACG |
| rec_293 | 15[256] | 16[256] | AAGGCGATAGATCGCA |
| rec_294 | 0[79] | 0[64] | TCAGCTAATGCAGAAC |
| rec_295 | 0[175] | 0[160] | TTAGGCAGAGGCATTT |
| rec_296 | 20[111] | 19[111] | CTTATGCGTAATCTTG |
| rec_297 | 17[112] | 17[127] | CCACTACGAAGGCACC |
| rec_298 | 13[304] | 12[304] | GTTTGCGTGTGAGACG |
| rec_299 | 4[223] | 5[223] | TTGAATACCCTGATTA |
| rec_300 | 11[224] | 12[224] | AAAGGGATAGCAGGCG |
| rec_301 | 13[176] | 13[191] | CACCAGTAGGGGTGCC |
| rec_302 | 2[271] | 1[271] | CTTAGATTTTAGTTAA |
| rec_303 | 3[64] | 4[64] | CCTGAATCCAGAGAGA |
| rec_304 | 22[303] | 21[303] | TTTCATTCTAAATCGG |
| rec_305 | 22[111] | 21[111] | GTTTAGACTTCAACTA |
| rec_306 | 23[224] | 23[239] | AGAGAGTACCTTTAAT |
| rec_307 | 15[64] | 16[64] | ATTGCGAAGCGAAAGA |
| rec_308 | 1[96] | 2[96] | TCAATAATGGCGTTTT |
| rec_309 | 1[80] | 0[80] | AGCATGTACAACATGT |
| rec_310 | 0[191] | 0[176] | AACGCCAACATGTAAT |
| rec_311 | 4[271] | 3[271] | CAGTACCTGAATTACC |
| rec_312 | 13[64] | 14[64] | CCGCCACCCTAAACAA |
| rec_313 | 10[111] | 9[111] | CAGAATGGGCCGCCAC |
| rec_314 | 1[224] | 2[224] | CGCAAGACCAAAATCA |
| rec_315 | 5[96] | 6[96] | TTTAAGAAGCAAAGAC |
| rec_316 | 6[207] | 5[207] | ATTATCATGAATTATC |
| rec_317 | 1[144] | 0[144] | CAAGTACCGTAATAAG |
| rec_318 | 0[287] | 1[287] | AAAAGCCTTCTGACCT |
| rec_319 | 16[255] | 17[255] | CTCCAGCCGTGAGCGA |
| rec_320 | 5[128] | 6[128] | TTACCAGACATACATA |
| rec_321 | 15[304] | 14[304] | TAAAACGAGACTCTAG |
| rec_322 | 10[271] | 9[271] | AACTATCGCAACAGAG |
| rec_323 | 3[224] | 4[224] | ATCAAGAATGATTGCT |
| rec_324 | 7[176] | 7[191] | AGCACCATAGCAAATG |
| rec_325 | 5[144] | 4[144] | GAGGAAACATAAAAAC |
| rec_326 | 19[224] | 20[224] | GGAGAGGGGTAAAGAT |
| rec_327 | 16[159] | 17[159] | AACCATCGAATACACT |
| rec_328 | 4[287] | 5[287] | GATGAATAAATGGAAG |
| rec_329 | 23[128] | 23[143] | AAGATTAAGAGGAAGC |
| rec_330 | 5[80] | 4[80] | TCTTACCGAATTGAGC |
| rec_331 | 14[255] | 15[255] | TTCCTGTGTGTGCTGC |
| rec_332 | 5[240] | 4[240] | CATCAATACAATAACG |
| rec_333 | 11[272] | 10[272] | TTATAATCAGAACTCA |
| rec_334 | 6[303] | 5[303] | ATTTGAGGCCTACCAT |
| rec_335 | 14[79] | 13[79] | GGATTTTGCTCAGAAC |
| rec_336 | 8[287] | 9[287] | ACATCGCCCTTCTGAC |
| rec_337 | 22[287] | 23[287] | AGTTGATTAGCTTAAT |
| rec_338 | 22[271] | 21[271] | TGCGAACGAAATTAAG |
| rec_339 | 9[96] | 10[96] | CCCTCAGAAAAGCGCA |
| rec_340 | 1[128] | 2[128] | AGAACGGGTAGAAGGC |
| rec_341 | 4[95] | 5[95] | CAGAGGGTAAGCCCTT |
| rec_342 | 14[191] | 14[176] | TAAAGCCTCAAACTAC |
| rec_343 | 22[255] | 23[255] | TAGTTTGATTGATAAG |
| rec_344 | 18[95] | 19[95] | ACTTAGCCTGACCTTC |
| rec_345 | 5[288] | 6[288] | GGTTAGAAATTTAGAA |
| rec_346 | 3[288] | 4[288] | GTACATAATAACGTCA |
| rec_347 | 6[175] | 5[175] | GACTCCTTACCCAAAA |
| rec_348 | 5[256] | 6[256] | ATTGTTTGACAACTCG |
| rec_349 | 19[96] | 20[96] | ATCAAGAGATTTTAAG |
| rec_350 | 15[224] | 16[224] | TTACGCCATTCTGGTG |
| rec_351 | 2[191] | 2[176] | CTTAGGTTTTTCATCG |
| rec_352 | 10[191] | 10[176] | ACATTTTGCATTGACA |
| rec_353 | 22[191] | 22[176] | GGCGCGAGGACGACGA |
| rec_354 | 21[112] | 20[112] | ATGCAGATTGAATTAC |
| rec_355 | 12[271] | 11[271] | CTGAGAGAAAGTGTTT |
| rec_356 | 15[176] | 15[191] | TTGATACCCAACTGTT |
| rec_357 | 20[95] | 21[95] | AACTGGCTATTTAGGA |
| rec_358 | 12[303] | 11[303] | GGCAACAGAAAAGAGT |
| rec_359 | 18[175] | 17[175] | CCAAGCGCCATCTTTG |
| rec_360 | 21[272] | 20[272] | CAATAAAGTTTTAGAA |
| rec_361 | 7[256] | 8[256] | TGGCAAATGAACCACC |
| rec_362 | 5[176] | 5[191] | GAACTGGCACCACCAG |
| rec_363 | 15[208] | 14[208] | GCGGGCCTCATACGAG |
| rec_364 | 18[271] | 17[271] | GTATAAGCCCGTCGGA |
| rec_365 | 8[223] | 9[223] | TATTAACATGGCAGAT |
| rec_366 | 1[272] | 0[272] | TTTCATCTGTTTAGTA |
| rec_367 | 12[191] | 12[177] | CAAAATCCCTTATAA |
| rec_368 | 12[142] | 11[143] | AGACTCCGAAACATG |
| rec_369 | 11[177] | 11[191] | AGAGTTCCTCGTTAG |
| rec_370 | 13[161] | 14[160] | AACACTGTAGCATTC |

**Supplementary note 2: Effect of fluorophore selection on junction formation**

Effect of fluorophore selection on junction folding. In order to ensure that the observed results of K1 were not influenced by the different fluorophores, since they are different in size and chemical properties, we redesigned K1 to contain 5’-FAM fluorophores and 3’-dark quencher on all four strands (termed K1FAM, second junction from top), and repeated all our experiments with each arm measured in a separate experiment. Comparing the measured Tm results of separate arms **(Fig. 2S.a)** and arms in junction structure **(Fig. 2S.b)** revealed differences in few degrees between Tm measured by the four different fluorophores used in the previous set of experiment and the FAM-labeled ones. According to the results, the Tj of K1FAM is 69 °C, as opposed to 73 °C measured in K1.

These results indicated that the fluorophores themselves had influence on strand hybridization and junction formation. Since the results of K1FAM folding showed higher correlation to the predicted Tm and melting profile than K1, and in order to eliminate any unrelated disturbance we decided to continue our work only with 5’-FAM (
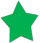
), a widely-used fluorescein derivative, and 3’-Iowa Black FQ quencher (
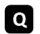
/
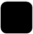
).


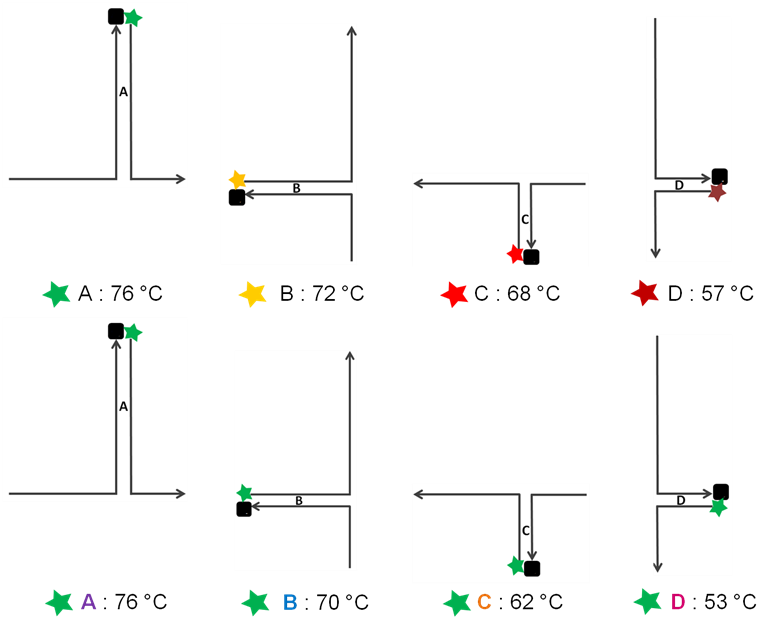

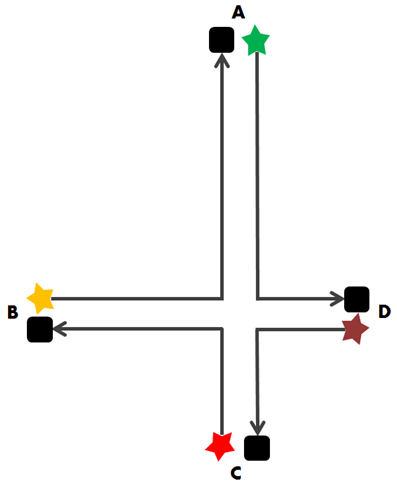

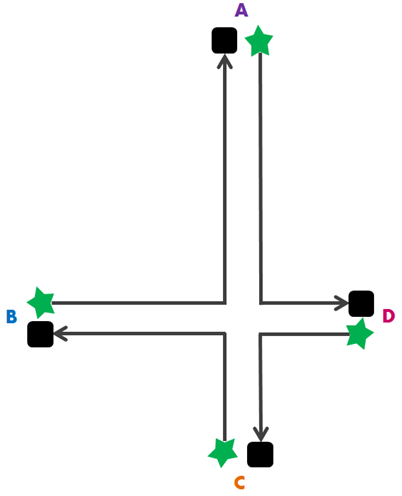

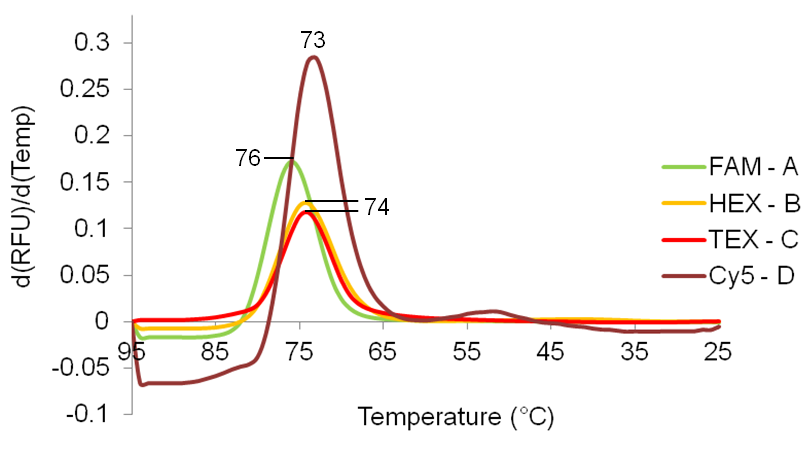

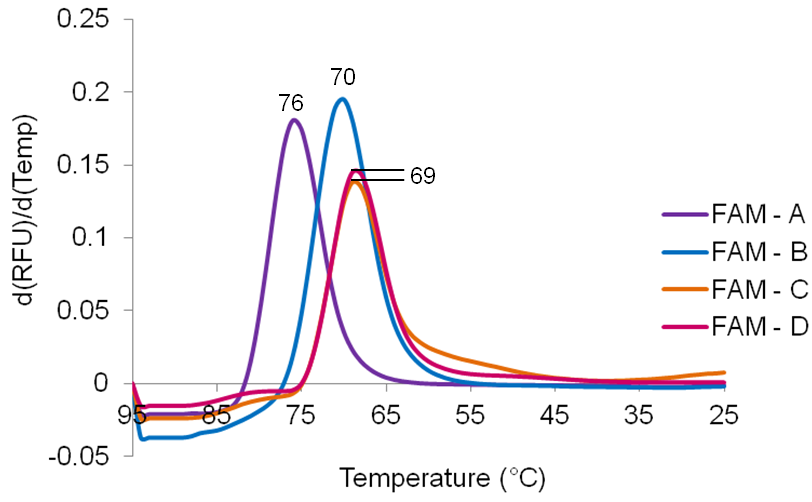


**(a)**

**(b)**

**Figure S2 |** Fluorophore effect on measured T_m_. Comparison between measured T_m_ in K1 and K1FAM . **(a).** T_m_ of separate arms. **(b)** T_m_ of arms in junction structure.

**Supplementary note 3: Design considerations in junction K3**

**(a)**


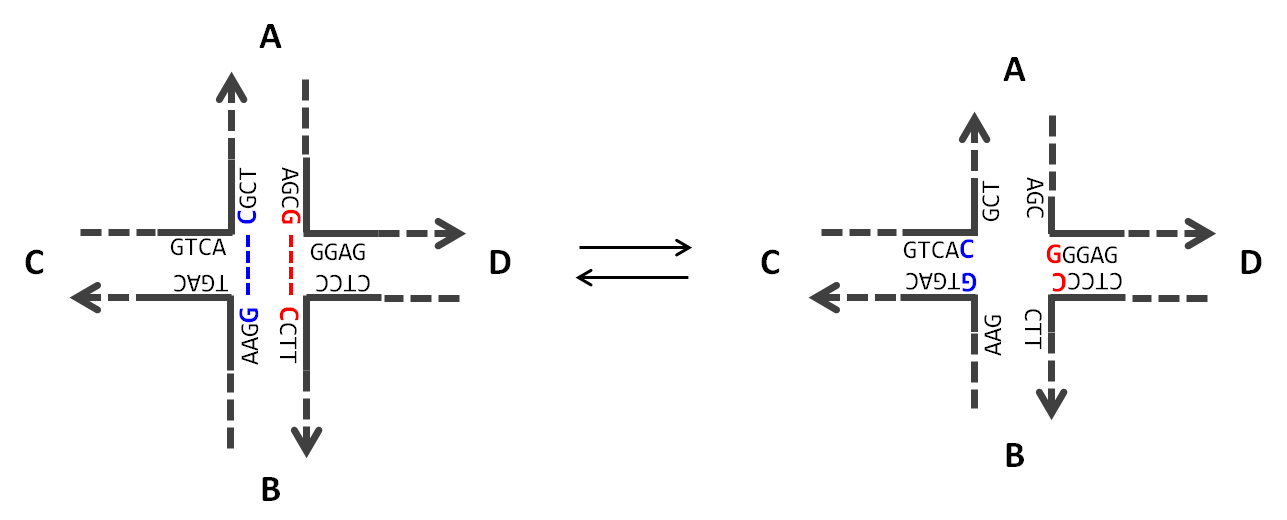


**(b)**


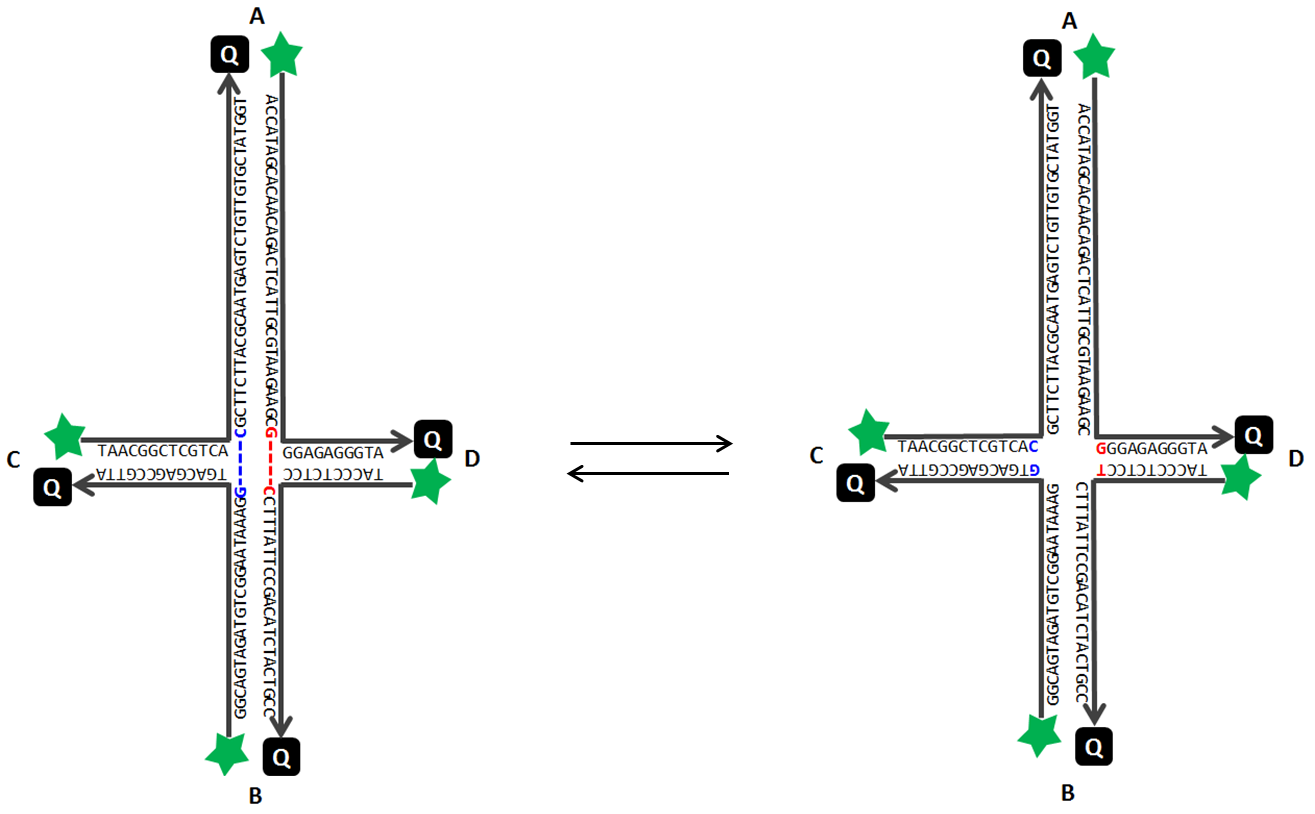


**Figure S3 |** **(a)** Zoom in on the bases that flank the semi mobile K3 junction. This junction is able to accomplish a flip-flop as indicated in the figure, but it is incapable of complete resolution, because the base pairs which flank the mobile bases (blue and red) now abut the junction. (**b**)

A semi-mobile junction K3. This junction may undergo the reactions indicated by the dash red and blue line, but may not go beyond them and resolve into two linear duplexes. As a result arms A and B get shorter by one base.

**Supplementary note 4: Junction labeling in DNA origami shape**

The number indicates the junction inside the 4J system, while the R/L represents the right or left strands that build the junction. For example: sequences used for measuring T_j_ of J1 junction:

Left diagonal:


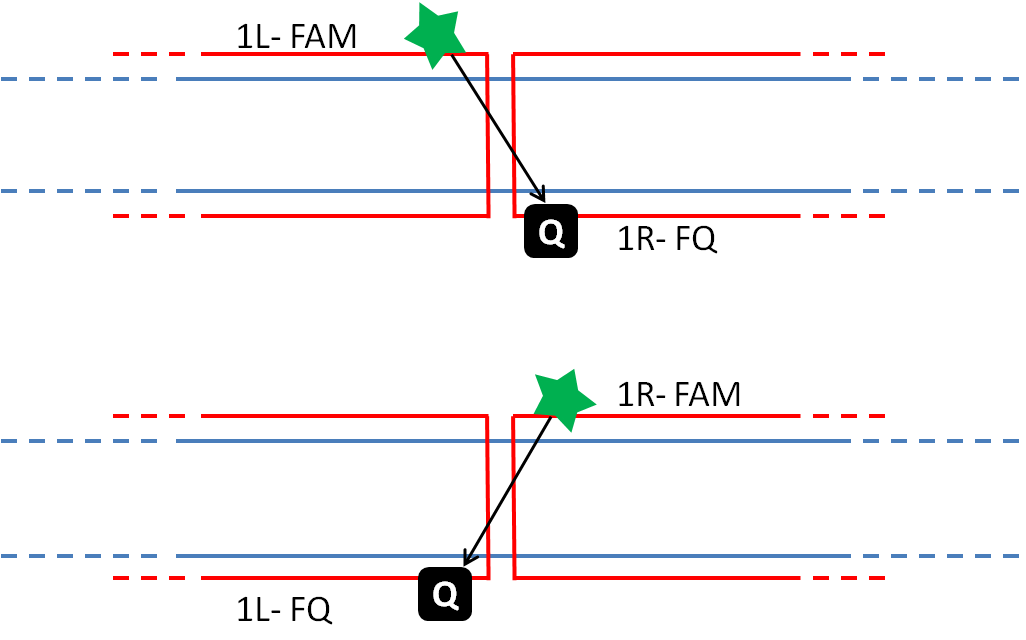


Right diagonal:


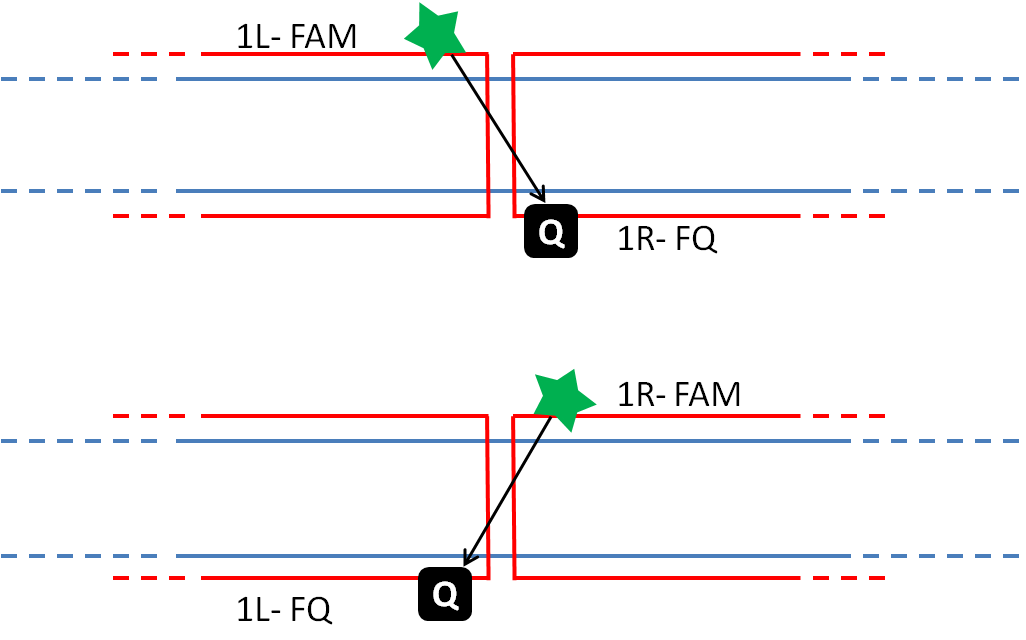


**Supplementary note 5: Gel electrophoresis results for reconstructed rectangle**

**(a) 1X “Folding buffer” as described in methods section**

Scaffold – Staples concentrations: 10 nM – 100 nM


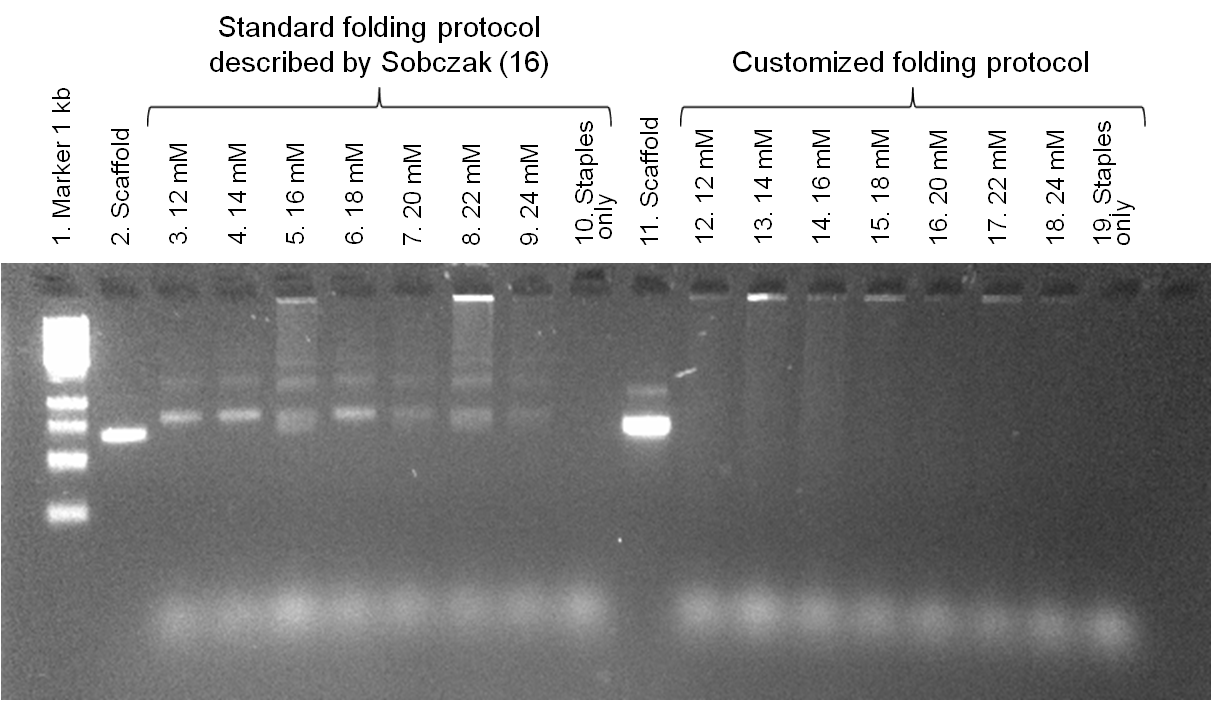


Scaffold – Staples concentrations: 20 nM – 200 nM


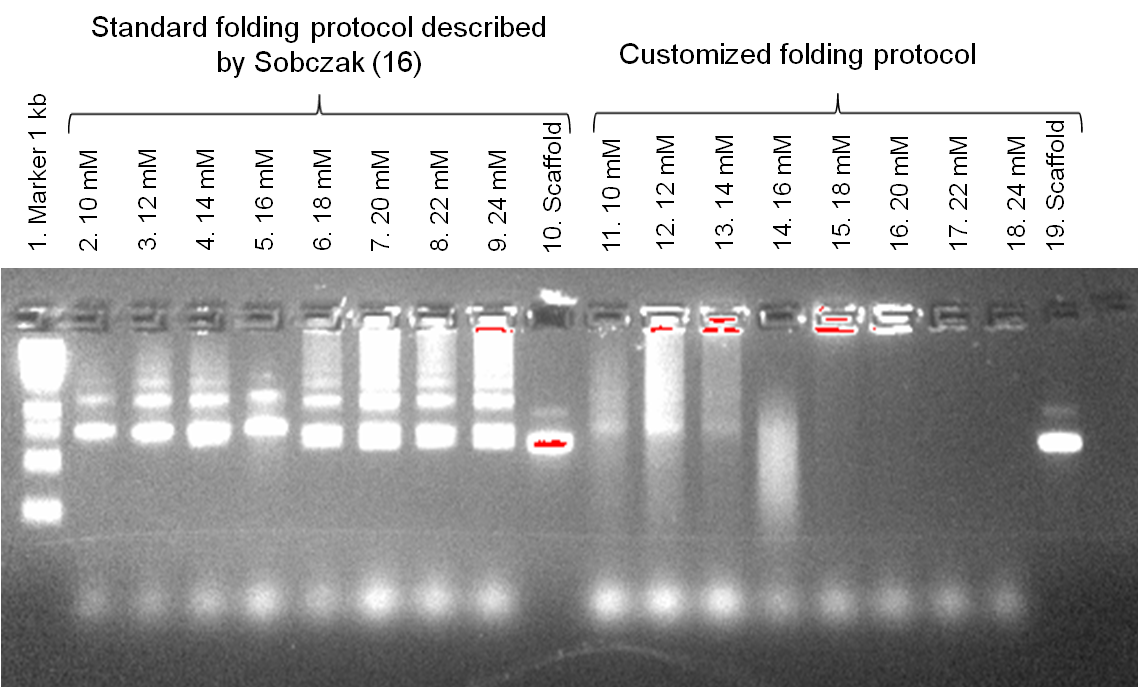


**(b) 1X TAE buffer**

Scaffold – Staples concentrations: 10 nM – 100 nM


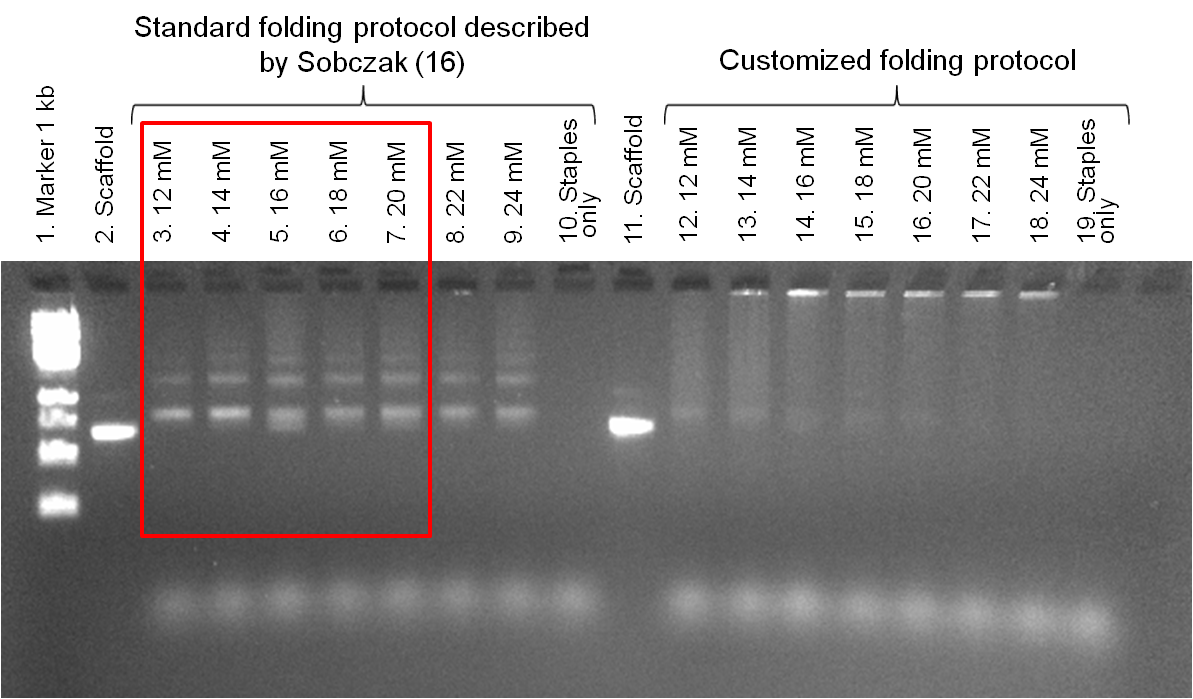


Scaffold – Staples concentrations: 20 nM – 200 nM


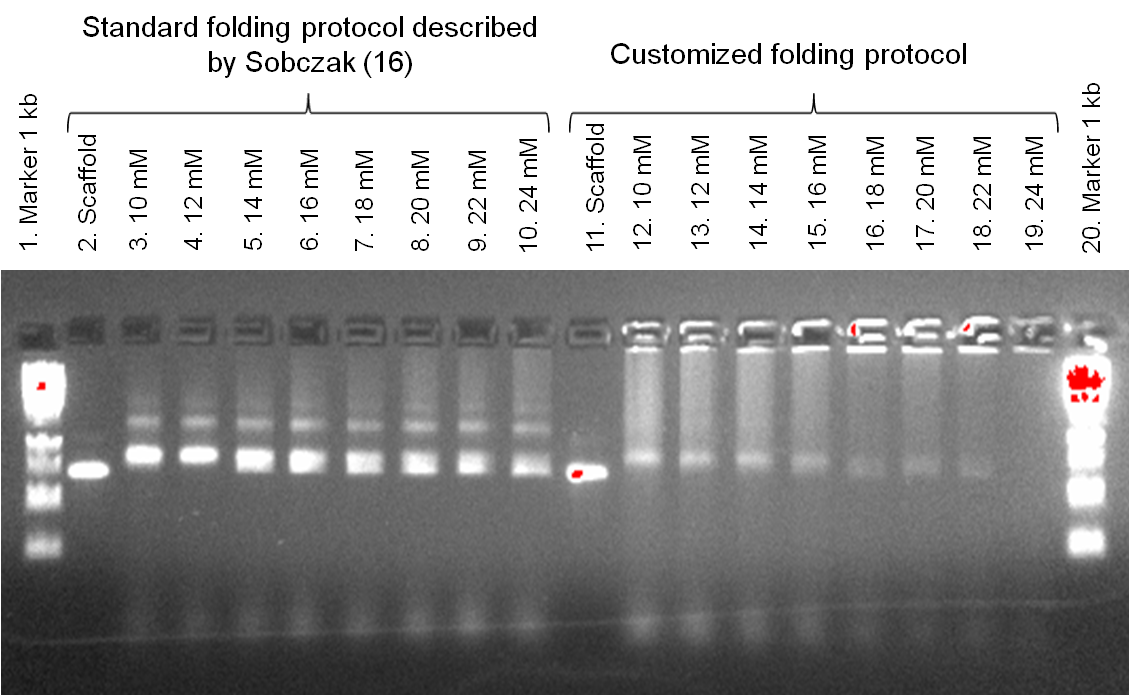


**Figure S5 |** Comparison between folding protocols, buffer and magnesium chloride concentrations. **(a)** Set of folding reaction in 1 x “folding buffer”. Top gel: scaffold and staples concentrations are 10 nM and 100 nM respectively. Bottom gel: scaffold and staples concentrations are 20 nM and 200 nM respectively. **(b)** Set of folding reaction in 1 x TAE. Top gel: scaffold and staples concentrations are 10 nM and 100 nM respectively. Bottom gel: scaffold and staples concentrations are 20 nM and 200 nM. Selected reactions for AFM analysis marked in red.

**Supplementary note 6: Model design and code**

The model was written in Perl (<http://www.perl.org/>), an object-oriented programming language which was used early on in the project to build some of its preliminary aspects.

The model is compatible with DNA shapes designed by caDNAno, taking two inputs from the user: 1) the caDNAno .json file, and 2) the .csv staple list exported by caDNAno as the final stage of the design process. Before running the model, the files must be converted into a .txt format. In addition, the annealing temperature range must be inserted by the user. The model disassembles the shape into DNA junctions and single staples which are not part of any junction. After this, it applies all the knowledge obtained experimentally, and performs a comprehensive analysis of the shape, when the main and final results are the simulation of the kinetics during the folding process and prediction of the critical temperature. These can optimize the folding process, since folding can be carried out by simply maintaining the DNA mixture at that temperature for a short period of time. Moreover, the model provides additional information such as more accurately predicted T_m_ of the folding regions, predicted T_j_ of each junction and their division to types – “K1”, “K2”, “K3” or “J1”, etc.

The model consists of three main parts. The first part parses the .json input file and creates a full mapping of all the folding regions and crossovers within the analyzed shape. Importantly, the model takes into account only crossovers made by two strands and not one. The second part analyzes this information and breaks the shape down into DNA junctions and single staples, while the third part runs the folding simulation.

**Part 1 : Mapping folding regions and crossovers.** The .json file supplies all the needed information, however it needs to be parsed in order to obtain the necessary data and be stored differently. The model iterates over each helix and position and builds a hash that saves the coordinates of all folding regions and crossover positions. For the folding regions it saves additional parameter which is the length.

**Part 2: disassembly of the shape into junctions and single staples.** This step relays on the results of part 1. Iterating over these results enables to map the shape to junctions, its basic building blocks. Staples that are not part of a full junction (junction with four arms) are termed “single staple”. During this process the analyzed data is cross-checked with the information received in the staple list input file, what adds additional information which may be used for further investigations giving a full analysis of the shape. For each folding region or a single staple the information that the model saves is:

- Helix number
- Start and end positions
- Length and sequence
- Number of A, T, C, G bases
- Predicted T_m_

While for each DNA junction it saves:

- Two helixes numbers
- Two position
- Predicted T_j_
- Junction type (“K1”, “K2”, “K3” or “J1”, with J1 including also nearly-symmetric junctions in which two arms or more are identical in terms of T_m_)

References to its four folding regions (arms)

Two assumptions were used in this part, both of them related to the calculation of the predicted T_m_. Since we noticed that there is a significant gap between the equation we normally used and the T_m_ predicted by NUPACK, while NUPACK code is more accurate as seen in previous observations we added a correction to the standard equation that aligns it with the NUPACK -predicted T_m_ **(Figure S5a)**. Second correction was to adjust the predicted NUPACK T_m_ to the observed T_m_ **(Figure S5b).** The sequences that used for these corrections are listed in **Supplementary note 1.**


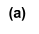

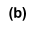


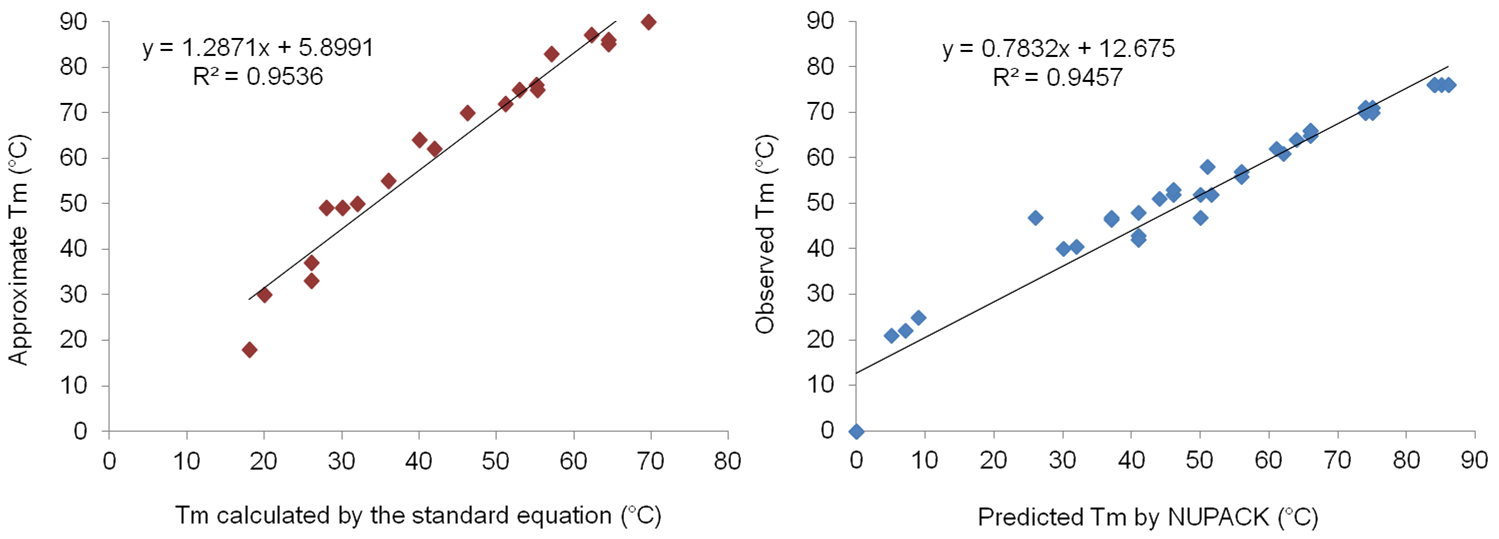


**Figure S5 |** Two correction to the standard T_m_ equation. (**a**) First correction: a correlation between standard equation and NUPACK. (**b**) Second correction: a correlation between predicted T_m_ by NUPACK and observed T_m_.

**Part 3: Folding process simulation.** After parsing all the needed information, the model starts to iterate over the temperature range inserted by the user, which simulates the folding protocol, not taking into account the time spent at each temperature.

Few more rules, which are based on our findings, were applied in this part:

1. In asymmetric junctions, according to K1, K2 and K3 analysis, the junction forms at T_m_ of “arm C” as measured in the junction structure and not separately. Therefore each junction initial T_j_ = T_m (arm c separately) +_ Δ_C_, where Δ_C_ is the difference in T_m_ of arm C when measured separately or in junction. For junction types “K1” and “K2” the Δ_C_ = 7 °С, where as for “K3” type Δ_C_ = 2 °С.
2. Junctions that have at least two arms with the same predicted T_m_ are classified as “J1” type and their folding T_j_ = ( T_m ( arm A separately ) +_ T_m ( arm B separately )_ )/2.
3. According to the cooperativity findings for 4J system outside and inside the rectangle shape, each assembled junction facilitates the assembly of neighboring unfolded junctions. In addition, junctions with higher T_j_ contribute more than smaller junctions with lower T_j_. To introduce this rule we devised the following equation: Δ_cooperativity_ = ( T_j1_ - T_j2_ ) $\times$ 0.9 , where T_j1_ is the approximated folding temperature of the donor Δ_cooperativity_ junction, and T_j2_ is the approximated folding temperature of the neighboring acceptor junction. Each junction might have up to four neighboring junctions.
4. As seen in 4J system experiments, J3 did not contribute to the formation of J4, since both are small junctions and similar in their T_j_ ( T_j3_ - T_j4_ = 4.5 °С ). Therefore the donor junction contributes Δ_cooperativity_ only if ( T_j1_ - T_j2_ ) > 4.5 °С.
5. Adding the Δ_cooperativity_ to a T_j_ of the neigbouring junction, may result in:

( T_j(neighbor)_+ Δ_cooperativity_ ) >= current temperature. If so, we add a “fee” of –1 °С for junction of types “K1”, ”K2” and “J1”, meaning that they will be folded at (current temperature – 1 °С), where as for “K3” type junctions, the fee set to be -3 °С (current temperature – 3 °С). These is according to the single junctions and 2J system observations that indicated that ”K3” junctions are folded at lower T_j_ and has more difficulty to fold. If a junction receives additional Δ_cooperativity_ from another neighboring junction, 1 °С is added to its fee. If the addition of 1 °С sets the fee = 0, then the junction folds at the current temperature.

1. Junction folds in two cases:

- T_j_ >= current temperature and fee = 0.
- At least three arms have been folded, regardless of the fee for junction of type “K1”, “K2” and “J1”. For type “K3” it folds at the current checked temperature unless it has a fee = -3 °С (maximal fee). In such case it will fold in the next temperature.

It is important to note, that a junction can fold without reaching its T_j_ due to its neighboring junctions, which fold at higher T_j_. For example it may happen when a junction with short arms surrounded by neighboring junctions with long arms with significantly higher T_j_.

This part of the algorithm is recursive. Each junction is checked at least once at each temperature until it is folded. When a junction forms, another recursive process starts since new regions are getting folded, this may result in three folded arms in neighboring junctions, which leads to their folding and so on. At each temperature, the algorithm calculates the % of folding process, based on the % of folded regions and single staples. We assume that the critical temperature of a shape is when ~50% of the shape is folded.

**Folding simulations.** In order to check our new model, we performed a comprehensive analysis and a folding simulation on three previously studied shapes (Sobczak et al., 2012) with known critical temperature of folding: Single layer rectangle, Brick and Gear

According to recent studies the Gear folds within the interval 58 °С – 50 °С (critical temperature ~53 °С), Brick within 56 °С – 47 °С (critical temperature ~52 °С) and Single layer rectangle within 64 °С – 50 °С (critical temperature ~60 °С) (Sobczak et al., 2012). Our model correlated well with these observations**.** It can be seen that also according to the simulated results, most of the folding process of each shape occurs in these temperature ranges. Moreover, the model predicted exactly the critical temperature of the Gear, while for the Brick and the single layer rectangle shape there was a slight deviation of -5  С (Brick 47°С, Single layer rectangle 55 °С).

Model code written in Perl programming language:

#!/usr/bin/perl -w

**use** strict**;**

**use** warnings**;**

**use** List**::**MoreUtils qw(uniq)**;**

**use** GD**::**Simple**;**

# USER INPUTS -------------------------------------------------------------

# .json and .csv staple list exported by caDNAno and converted to .txt files

**print** "Insert the Jason file name:"**;**

**my** $jasonFileName **=** <>**;**

**chomp** $jasonFileName**;** #chomps the \n

**print** "Insert the StapleList file name:"**;**

**my** $StaplesListFileName **=** <>**;**

**chomp** $StaplesListFileName**;**

**print** "Insert the initial TMP:"**;**

**my** $initialTMP **=** 0**;**

$initialTMP **=** <>**;**

**chomp** $initialTMP**;**

**print** "Insert the final TMP:"**;**

**my** $finalTMP **=** 0**;**

$finalTMP **=** <>**;**

**chomp** $finalTMP**;**

###########################################################################

# Part1 -This part creates a full mapping of all the folding regions and #crossovers within the analyzed shape

###########################################################################

# for staple list parsing

**my** $part**;** **my** $num**;**

**my** $partLenght **=** 0**;** **my** $pos **=** 0**;**

**my** $numOfHelixes **=** 0**;**

**my** $totalNumOfStapleParts **=** 0**;**

**my** $p_num**;** **my** $p_pos**;** **my** $n_num**;** **my** $n_pos**;**

**my** $RE_pos**;** **my** $RE_p_num**;** **my** $RE_p_pos**;** **my** $RE_n_num**;** **my** $RE_n_pos**;**

**my** $numA **=** 0**;** **my** $numT **=** 0**;** **my** $numC **=** 0**;** **my** $numG **=** 0**;** **my** $tmTmp **=** 0**;**

**my** $lenghtOfStapleList **=** 0**;**

**my** $staples **=** ""**;**

**my** $substrStart **=** 'stap":['**;**

**my** $substrEnd **=** ']],"'**;**

**my** $offsetStart **=** 0**;**

**my** $offsetEnd **=** 0**;**

**my** $resultEnd **=** 0**;**

**my** @tmpStaples**;**

**my** @staplePartsPerNum**;**

**my** %StaplesPartsIndex**;**

**my** %ScaffoldPartsIndex**;**

# for scaffold list parsing

**my** $scaf_p_num**;** **my** $scaf_p_pos**;** **my** $scaf_n_num**;** **my** $scaf_n_pos**;** **my** $partScaf **=** ''**;**

**my** $scaf_RE_pos**;** **my** $scaf_RE_p_num**;** **my** $scaf_RE_p_pos**;** **my** $scaf_RE_n_num**;** **my** $scaf_RE_n_pos**;**

**my** $scaf **=** ""**;**

**my** $lenghtOfScafList **=** 0**;**

**my** $substrStartSacf **=** 'scaf":['**;**

**my** $substrEndScaf **=** ']],"'**;**

**my** $offsetStartScaf **=** 0**;**

**my** $offsetEndScaf **=** 0**;**

**my** $resultEndScaf **=** 0**;**

**my** $ScafPartLenght **=** 0**;**

**my** @scaffoldPerNum**;**

# for scaffold list parsing

**my** $substrNUM **=** 'num":'**;**

**my** $substrNUM_END **=**','**;**

**my** $offsetNUM **=** 0**;**

**my** $lenghtofNUM **=** 0**;**

**my** $resultNUM **=** **-**1**;**

**my** $resultNUMEND **=** **-**1**;**

**my** $helixLenght **=** 0**;**

**my** $runningIndexFrom0 **=** 0**;**

**my** %numOrderInShape**;**

#--------------------------------------------------------------------------

**open(**FILE**,** $jasonFileName**)** **or** **die** "Can't read file $jasonFileName [$!]\n"**;**

**my** $fullJasonFile **=** <FILE>**;**

**close** **(**FILE**);**

**my** $resultStart **=** **index(**$fullJasonFile**,**$substrStart**,** $offsetStart**);**

**my** $resultStartScaf **=** **index(**$fullJasonFile**,**$substrStartSacf**,** $offsetStartScaf**);**

**my** $flag_Scaf_CO**;**

**while** **(**$resultStart **!=** **-**1**)** **{**

$pos **=** 0**;**

$numOfHelixes **=** $numOfHelixes **+** 1**;**

$lenghtOfStapleList **=** 0**;**

$lenghtofNUM **=** 0**;**

$resultEnd **=** **index(**$fullJasonFile**,**$substrEnd**,** $resultStart**);**

$resultEndScaf **=** **index(**$fullJasonFile**,**$substrEndScaf**,** $resultStartScaf**);**

$resultNUM **=** **index(**$fullJasonFile**,**$substrNUM**,** $offsetNUM**);**

$resultNUMEND **=** **index(**$fullJasonFile**,**$substrNUM_END**,** $resultNUM**);**

$lenghtofNUM **=** $resultNUMEND **-** **(**$resultNUM **+** 5**)** **;**

$num **=** **substr** $fullJasonFile**,** $resultNUM **+** 5**,** $lenghtofNUM**;**

**if** **(**$num **=~** /}]/**)** **{**

$num **=** **substr** $num**,** 0**,** $lenghtofNUM **-** 2 **;}**

**elsif(**$num **=~** /}/**)** **{**

$num **=** **substr** $num**,** 0**,** $lenghtofNUM **-** 1 **;}**

$numOrderInShape**{**$num**}** **=** $runningIndexFrom0**;**

$offsetStart **=** $resultStart **+** 1**;**

$offsetStartScaf **=** $resultStartScaf **+** 1**;**

$offsetNUM **=** $resultNUM **+** 1**;**

$lenghtOfStapleList **=** $resultEnd**-(**$resultStart **+** 8**)** **+** 1**;**

$lenghtOfScafList **=** $resultEndScaf**-(**$resultStartScaf **+** 8**)** **+** 1**;**

$staples **=** **substr** $fullJasonFile**,** $resultStart **+** 8**,** $lenghtOfStapleList**;**

@staplePartsPerNum **=** **split** **(**',\['**,**$staples**);**

$scaf **=** **substr** $fullJasonFile**,** $resultStartScaf **+** 8**,** $lenghtOfScafList**;**

@scaffoldPerNum **=** **split** **(**',\['**,**$scaf**);**

**foreach** $part **(**@staplePartsPerNum**)** **{**

$partScaf **=** $scaffoldPerNum**[**$pos**];**

$flag_Scaf_CO **=** 0**;**

# checking the SCAFFOLD part --------------------------------------

$partScaf **=** **substr** $partScaf **,** 0**,** **-**1**;** # cutting the last ']' **(**$scaf_p_num**,**$scaf_p_pos**,** $scaf_n_num**,**$scaf_n_pos**)** **=** **split** **(**','**,** $partScaf**);**

**if** **(**$scaf_p_num **==** **-**1 **&&** $scaf_n_num **==** **-**1 **&&** $scaf_p_pos **==** **-**1 **&&** $scaf_n_pos **==** **-**1**)** **{}**

# in case of a new scaffold part

**elsif** **(**$ScafPartLenght **==** 0**)** **{**

$scaf_RE_pos **=** $pos**;**

$scaf_RE_p_num **=** $scaf_p_num**;**

$scaf_RE_p_pos **=** $scaf_p_pos**;**

$scaf_RE_n_num **=** $scaf_n_num**;**

$scaf_RE_n_pos **=** $scaf_n_pos**;**

$ScafPartLenght **=** $ScafPartLenght **+** 1 **;**

**}**

# continue in the same staple part

**elsif** **(**$scaf_p_num **==** $scaf_n_num **&&** $scaf_p_num **!=** **-**1 **&&** $scaf_n_num **!=** **-**1**)** **{**

$ScafPartLenght **=** $ScafPartLenght **+** 1 **;**

**}**

# Crossover point : the scaffold part ends

**else** **{**

$ScafPartLenght **=** $ScafPartLenght **+** 1 **;**

**my** @ScafREarr **=** **(**$scaf_RE_p_num**,** $scaf_RE_p_pos**,** $scaf_RE_n_num**,** $scaf_RE_n_pos**,** $ScafPartLenght**);**

$ScaffoldPartsIndex**{**$num**}{**$scaf_RE_pos**}** **=** **\**@ScafREarr**;**

**my** @Scafarr **=** **(**$scaf_p_num**,** $scaf_p_pos**,** $scaf_n_num**,** $scaf_n_pos**,** $ScafPartLenght**);**

$ScaffoldPartsIndex**{**$num**}{**$pos**}** **=** **\**@Scafarr**;**

$ScafPartLenght **=** 0**;**

**if** **(** $scaf_p_num **!=** $scaf_n_num **)** **{**

$partScaf **=** $scaffoldPerNum**[**$pos**+**1**];**

$partScaf **=** **substr** $partScaf **,** 0**,** **-**1**;**

**(**$scaf_p_num**,**$scaf_p_pos**,** $scaf_n_num**,**$scaf_n_pos**)** **=** **split** **(**','**,** $partScaf**)** **;**

**if** **(**$scaf_p_num **!=** **-**1 **&&** $scaf_n_num **!=** **-**1 **&&** $scaf_p_pos **!=** **-**1 **&&** $scaf_n_pos **!=** **-**1**)** **{**

$flag_Scaf_CO **=** 1**;**

**}**

$partScaf **=** $scaffoldPerNum**[**$pos**-**1**];**

$partScaf **=** **substr** $partScaf **,** 0**,** **-**1**;**

**(**$scaf_p_num**,**$scaf_p_pos**,** $scaf_n_num**,**$scaf_n_pos**)** **=** **split** **(**','**,** $partScaf**)** **;**

**if** **(**$scaf_p_num **!=** **-**1 **&&** $scaf_n_num **!=** **-**1 **&&** $scaf_p_pos **!=** **-**1 **&&** $scaf_n_pos **!=** **-**1**)** **{**

$flag_Scaf_CO **=** 1**;}**

**}**

**}**

# checking the STAPLE part ----------------------------------------

$part **=** **substr** $part**,** 0**,** **-**1**;**

**(**$p_num**,**$p_pos**,** $n_num**,**$n_pos**)** **=** **split** **(**','**,** $part**);**

**if** **(**$p_num **==** **-**1 **&&** $n_num **==** **-**1 **&&** $p_pos **==** **-**1 **&&** $n_pos **==** **-**1**)** **{**

$pos **=** $pos **+** 1**;**

**next;}**

# new staple part

**elsif** **(**$partLenght **==** 0**)** **{**

$RE_pos **=** $pos**;**

$RE_p_num **=** $p_num**;**

$RE_p_pos **=** $p_pos**;**

$RE_n_num **=** $n_num**;**

$RE_n_pos **=** $n_pos**;**

$partLenght **=** $partLenght **+** 1**;**

**if** **(**$flag_Scaf_CO **==** 1**)**

**{**

**my** @REarr **=** **(**$RE_p_num**,** $RE_p_pos**,** $RE_n_num**,** $RE_n_pos**,** $partLenght**);**

$StaplesPartsIndex**{**$num**}{**$RE_pos**}** **=** **\**@REarr**;**

$partLenght **=** 0**;}**

**}**

**elsif** **(**$p_num **==** $n_num **&&** $p_num **!=** **-**1 **&&** $n_num **!=** **-**1**)** **{**

$partLenght **=** $partLenght **+** 1 **;**

**(**$scaf_p_num**,**$scaf_p_pos**,** $scaf_n_num**,**$scaf_n_pos**)** **=** **split** **(**','**,** $scaffoldPerNum**[**$pos**]);**

**if** **(**$flag_Scaf_CO **==** 1**)**

**{**

**my** @REarr **=** **(**$RE_p_num**,** $RE_p_pos**,** $RE_n_num**,** $RE_n_pos**,** $partLenght**);**

$StaplesPartsIndex**{**$num**}{**$RE_pos**}** **=** **\**@REarr**;**

**my** @arr **=** **(**$p_num**,** $p_pos**,** $n_num**,** $n_pos**,** $partLenght**);**

$StaplesPartsIndex**{**$num**}{**$pos**}** **=** **\**@arr**;**

$partLenght **=** 0**;}**

**}**

# crossover point - The staple part ends:

**else** **{**

$partLenght **=** $partLenght **+** 1 **;**

**my** @REarr **=** **(**$RE_p_num**,** $RE_p_pos**,** $RE_n_num**,** $RE_n_pos**,** $partLenght**);**

$StaplesPartsIndex**{**$num**}{**$RE_pos**}** **=** **\**@REarr**;**

**my** @arr **=** **(**$p_num**,** $p_pos**,** $n_num**,** $n_pos**,** $partLenght**);**

$StaplesPartsIndex**{**$num**}{**$pos**}** **=** **\**@arr**;**

$partLenght **=** 0**;**

**}**

$pos **=** $pos **+** 1**;**

**}**

$resultStart **=** **index(**$fullJasonFile**,**$substrStart**,** $offsetStart**);**

$resultStartScaf **=** **index(**$fullJasonFile**,**$substrStartSacf**,** $offsetStartScaf**);**

$runningIndexFrom0 **+=** 1**;**

**if** **(** $helixLenght **<** $pos **)** **{**

$helixLenght **=** $pos**;}**

**}** #end of while

###########################################################################

# Part2 - This part analyzes this information and breaks the shape down into DNA junctions and single staples #

###########################################################################

# Letters-Matrix - Contains in each cell the letter "A,T,C,G"

**my** %letterMatrix**;**

# TM-Matrix - Contains in each cell the Melting Temperature of the staple's part

**my** %TMMatrix**;**

# TM Hash - saves the TM temperature in a hash structure

**my** %TMHash**;**

# CrossOverHash1 Hash - saves all the cross overs is the structure

**my** %crossOverHash1**;**

# CrossOverHash2. Saves just FULL CO for each crossover

**my** %crossOverHash2**;**

# staplePartsRefAndCO Hash - saves all the staple parts

**my** %staplePartsRefAndCO**;**

# saves the single staples that does not belong to any co

**my** %SingleStaples**;**

# saves a list of full staples

**my** %fullStapleHash**;**

# saves the junction mapping by type : K1, K2, K3 and J1

**my** %MatrixJunctionType**;**

# resting the hashes with initial values

**my** $row**;**

**my** $col**;**

**for** **(**$row **=** 0**;** $row **<** $numOfHelixes**;** $row**++)** **{**

**for** **(**$col **=** 0**;** $col **<=** $helixLenght**;** $col**++)** **{**

$letterMatrix**{**$row**}{**$col**}** **=** '-'**;** # saves the staples sequences

$TMMatrix**{**$row**}{**$col**}** **=** **-**200**;** #saves the TM temperature

**}**

**}**

**open(**SL_FILE**,** $StaplesListFileName**)** **or** **die** "Can't read file $StaplesListFileName [$!]\n"**;**

**my** $firstLine_headers **=** <SL_FILE>**;**

**my** @StapleStartEndPos**;**

**my** @tmp_StaplePart**;**

**my** $endPos**;** **my** $keyNum**;** **my** $keyPos**;**

**my** $flag_CO **=** 0**;**

**my** $numCO1**;** **my** $numCO2**;** **my** $posCO**;**

**my** $NumOfK1 **=** 0**;** **my** $NumOfK2 **=** 0**;** **my** $NumOfK3 **=** 0**;** **my** $NumOfJ1 **=** 0**;**

**while(**<SL_FILE>**)**

**{**

**chomp;**

@StapleStartEndPos **=** **split** **(**'\t'**,**$_**);**

**(**$num**,** **my** $tmpPos**)** **=** **split** **(**'\['**,**$StapleStartEndPos**[**0**]);**

**(**$pos**,** **my** $fld**)** **=** **split** **(**'\]'**,**$tmpPos**);**

$keyNum **=** $num**;**

$keyPos **=** $pos**;**

@tmp_StaplePart **=** @**{**$StaplesPartsIndex**{**$num**}{**$pos**}};**

**my** $fullStapleSeq **=** $StapleStartEndPos**[**2**];**

**my** $stapleLength**;** **my** $ref_CO**;** **my** $ref_prevPart**;**

**my** $leftToCopy **=** 0**;**

**my** $counter **=** 1**;**

**my** $flagScafCo **=** 0**;**

**my** $flag_1_base_staple **=** 0**;**

**my** @arr**;** **my** @tmpArr**;** **my** @arrTypeTMP**;**

**my** $TMP_File **=** "StapleTMP.txt"**;**

**open(**STAPLES_TMP**,** ">>$TMP_File"**)** **or** **die** "Can't read file $TMP_File [$!]\n"**;**

**while** **(**$tmp_StaplePart**[**2**]** **!=** **-**1 **&&** $tmp_StaplePart**[**3**]** **!=** **-**1 **||** **(** $tmp_StaplePart**[**4**]** **==** 1 **&&** $flag_1_base_staple **==** 0**))**

**{**

**my** @part**;**

**if** **(** **(**$num **eq** $tmp_StaplePart**[**2**]** **||** $tmp_StaplePart**[**4**]** **==** 1 **)&&** $flagScafCo **==** 0**)** **{**

$flagScafCo **=** 1**;**

**my** $partSeq **=** **substr** $fullStapleSeq**,** 0**,** $tmp_StaplePart**[**4**];**

$stapleLength **=** **length(**$fullStapleSeq**);**

$leftToCopy **=** $stapleLength**-**$tmp_StaplePart**[**4**];**

**if** **(**$leftToCopy **>** 0**)** **{**

$fullStapleSeq **=** **substr** $fullStapleSeq**,** $tmp_StaplePart**[**4**],** $leftToCopy**;}**

**my** @letters **=** **split** **(**/(\w|\W)/**,**$partSeq**);**

$counter **=** 1**;**

$numA **=** 0**;** $numT **=** 0**;** $numC **=** 0**;** $numG **=** 0**;**

$tmTmp **=** 0**;**

# checking staple direction

**if** **(** $pos **>** $tmp_StaplePart**[**3**]** **)** **{**

$endPos **=** $pos **-** $tmp_StaplePart**[**4**]** **+** 1**;**

**for** **(my** $col **=** $pos**;** $col **>=** $endPos**;** $col**--)** **{**

$letterMatrix**{**$num**}{**$col**}** **=** $letters**[**$counter**];**

**if** **(**$letters**[**$counter**]** **eq** 'A'**)** **{**

$numA **=** $numA **+** 1**;}**

**if** **(**$letters**[**$counter**]** **eq** 'T'**)** **{**

$numT **=** $numT **+** 1**;}**

**if** **(**$letters**[**$counter**]** **eq** 'C'**)** **{**

$numC **=** $numC **+** 1**;}**

**if** **(**$letters**[**$counter**]** **eq** 'G'**)** **{**

$numG **=** $numG **+** 1**;}**

$counter **=** $counter **+** 2**;**

**}**

**}**

**else** **{**

$endPos **=** $pos **+** $tmp_StaplePart**[**4**]** **-** 1**;**

**for** **(my** $col **=** $pos**;** $col **<=** $endPos**;** $col**++)** **{**

$letterMatrix**{**$num**}{**$col**}** **=** $letters**[**$counter**];**

**if** **(**$letters**[**$counter**]** **eq** 'A'**)** **{**

$numA **=** $numA **+** 1**;}**

**if** **(**$letters**[**$counter**]** **eq** 'T'**)** **{**

$numT **=** $numT **+** 1**;}**

**if** **(**$letters**[**$counter**]** **eq** 'C'**)** **{**

$numC **=** $numC **+** 1**;}**

**if** **(**$letters**[**$counter**]** **eq** 'G'**)** **{**

$numG **=** $numG **+** 1**;}**

$counter **=** $counter **+** 2**;**

**}**

**}**

# calculating Melting Temp: depending on length - same calculation as in OligoCalc web

**if** **(**$tmp_StaplePart**[**4**]** **<** 14**)**

**{**

$tmTmp **=** 4***(**$numG **+** $numC**)** **+** 2***(**$numA **+** $numT**);}**

**else**

**{**

$tmTmp**=**64.9**+(**41***(**$numG**+**$numC**-** 16.4**)/**$tmp_StaplePart**[**4**]);}**

# correlation between Oligocalc and Nupack

$tmTmp **=** 1.2871***(**$tmTmp**)** **+** 5.8991**;**

# correlation between Nupack and observed

$tmTmp **=** 0.7832***(**$tmTmp**)** **+** 12.675**;**

**if** **(** $pos **>** $endPos **)** **{**

**for** **(** **my** $col **=** $pos**;** $col **>=** $endPos**;** $col**--** **)** **{**

$TMMatrix**{**$num**}{**$col**}** **=** $tmTmp**;}**

**}**

**else** **{**

**for** **(** **my** $col **=** $pos**;** $col **<=** $endPos**;** $col**++** **)** **{**

$TMMatrix**{**$num**}{**$col**}** **=** $tmTmp**;}**

**}**

@part **=** **(**$num**,** $pos**,** $endPos**,** $tmp_StaplePart**[**4**],** $partSeq**,** $numA**,** $numT**,** $numC**,** $numG**,** $tmTmp**,** 0**,**$tmTmp**);**

**print** STAPLES_TMP "$tmTmp\n"**;**

**push** **(**@arr**,** **\**@part**);**

$totalNumOfStapleParts **=** $totalNumOfStapleParts **+** 1**;**

**if** **(** $flag_CO **==** 0 **)** **{**

$ref_prevPart **=** **\**@part**;**

$flag_CO **=** 1**;}**

**if** **(** $flag_CO **==** 2**)** **{**

**if** **(** $ref_prevPart**->[**1**]** **<** $ref_prevPart**->[**2**]** **)** **{**

**if** **(exists** $crossOverHash1**{**$numCO2**}{**$numCO1**}{**$posCO**+**1**}{**$posCO**})** **{**

**if** **(** $ref_prevPart**->[**9**]** **>** $part**[**9**]** **)** **{**

**push** **(**$crossOverHash1**{**$numCO2**}{**$numCO1**}{**$posCO**+**1**}{**$posCO**},**$ref_prevPart**);**

**push** **(**$crossOverHash1**{**$numCO2**}{**$numCO1**}{**$posCO**+**1**}{**$posCO**},\**@part**);**

**}**

**else** **{**

**push** **(**$crossOverHash1**{**$numCO2**}{**$numCO1**}{**$posCO**+**1**}{**$posCO**},\**@part**);**

**push** **(**$crossOverHash1**{**$numCO2**}{**$numCO1**}{**$posCO**+**1**}{**$posCO**},**$ref_prevPart**);**

**}**

$ref_CO**=** $crossOverHash1**{**$numCO2**}{**$numCO1**}{**$posCO**+**1**}{**$posCO**};**

**&**sortingJunctionAndTj**(**$ref_CO**,**$posCO**+**1**,**$posCO**);**

**}**

**else** **{**

# new CO

**my** @coarr**;**

$coarr**[**0**]** **=** 0**;**

$coarr**[**1**]** **=** 0**;**

$coarr**[**2**]** **=** 0**;**

$coarr**[**3**]** **=** 0**;**

**if** **(**$ref_prevPart**->[**9**]** **>** $part**[**9**])** **{**

**push(**@coarr**,**$ref_prevPart**);**

**push(**@coarr**,\**@part**);** **}**

**else** **{**

**push(**@coarr**,\**@part**);**

**push(**@coarr**,**$ref_prevPart**);}**

$crossOverHash1**{**$numCO1**}{**$numCO2**}{**$posCO**}{**$posCO**+**1**}** **=** **\**@coarr**;**

$ref_CO**=** $crossOverHash1**{**$numCO1**}{**$numCO2**}{**$posCO**}{**$posCO**+**1**};**

**}**

**}**

**elsif** **(** $ref_prevPart**->[**1**]** **>** $ref_prevPart**->[**2**]** **)** **{**

**if(exists** $crossOverHash1**{**$numCO2**}{**$numCO1**}{**$posCO**-**1**}{**$posCO**})** **{**

**if** **(** $ref_prevPart**->[**9**]** **>** $part**[**9**]** **)** **{**

**push** **(**$crossOverHash1**{**$numCO2**}{**$numCO1**}{**$posCO**-**1**}{**$posCO**},**$ref_prevPart**);**

**push** **(**$crossOverHash1**{**$numCO2**}{**$numCO1**}{**$posCO**-**1**}{**$posCO**},\**@part**);}**

**else** **{**

**push** **(**$crossOverHash1**{**$numCO2**}{**$numCO1**}{**$posCO**-**1**}{**$posCO**},\**@part**);**

**push** **(**$crossOverHash1**{**$numCO2**}{**$numCO1**}{**$posCO**-**1**}{**$posCO**},**$ref_prevPart**);}**

$ref_CO**=** $crossOverHash1**{**$numCO2**}{**$numCO1**}{**$posCO**-**1**}{**$posCO**};**

**&**sortingJunctionAndTj**(**$ref_CO**,**$posCO**-**1**,**$posCO**);**

**}**

**else** **{**

**my** @coarr**;**

$coarr**[**0**]** **=** 0**;**

$coarr**[**1**]** **=** 0**;**

$coarr**[**2**]** **=** 0**;**

$coarr**[**3**]** **=** 0**;**

**if** **(**$ref_prevPart**->[**9**]** **>** $part**[**9**])** **{**

**push(**@coarr**,**$ref_prevPart**);**

**push(**@coarr**,\**@part**);**

**}**

**else** **{**

**push(**@coarr**,\**@part**);**

**push(**@coarr**,**$ref_prevPart**);**

**}**

$crossOverHash1**{**$numCO1**}{**$numCO2**}{**$posCO**}{**$posCO**-**1**}** **=** **\**@coarr**;**

$ref_CO**=** $crossOverHash1**{**$numCO1**}{**$numCO2**}{**$posCO**}{**$posCO**-**1**};**

**}**

**}**

**else** **{** **print** "ERROR!!!!\n"**;}**

**my** @staplePartCO_ref_arr1**;**

**my** @staplePartCO_ref_arr2**;**

**if** **(** **exists** $staplePartsRefAndCO**{**$ref_prevPart**}** **)** **{**

**push** **(**$staplePartsRefAndCO**{**$ref_prevPart**},**$ref_CO**);}**

**else** **{**

$staplePartCO_ref_arr1**[**0**]** **=** $ref_CO**;**

$staplePartsRefAndCO**{**$ref_prevPart**}=** **\**@staplePartCO_ref_arr1**;}**

**if** **(** **exists** $staplePartsRefAndCO**{\**@part**}** **)** **{**

**push** **(**$staplePartsRefAndCO**{\**@part**},**$ref_CO**);}**

**else** **{**

$staplePartCO_ref_arr2**[**0**]** **=** $ref_CO**;**

$staplePartsRefAndCO**{\**@part**}=** **\**@staplePartCO_ref_arr2**;}**

$ref_prevPart **=** **\**@part**;**

$flag_CO **=** 3**;**

**}**

$pos **=** $endPos**;**

**}**

**else**

**{** # a cross over point

**if** **(** $flag_CO **==** 1 **||** $flag_CO **==** 3 **)** **{**

$flag_CO **=** 2**;**

$numCO1 **=** $num**;**

**if** **(**$num **eq** $tmp_StaplePart**[**2**])** **{**

$numCO2 **=** $ScaffoldPartsIndex**{**$num**}{**$pos**}->[**2**];**

**if** **(**$numCO2 **eq** $numCO1**)** **{**

$numCO2 **=** $ScaffoldPartsIndex**{**$num**}{**$pos**}->[**0**];** **}**

**}**

# staple CO

**else** **{**

$numCO2 **=** $tmp_StaplePart**[**2**];}**

$posCO **=** $pos**;**

**}**

$num **=** $tmp_StaplePart**[**2**];**

$pos **=** $tmp_StaplePart**[**3**];**

$flagScafCo **=** 0**;**

**}**

**if** **(** $num**!=** **-**1 **&&** $pos **!=** **-**1 **)** **{**

@tmp_StaplePart **=** @**{**$StaplesPartsIndex**{**$num**}{**$pos**}};**

$flag_1_base_staple **=** 0**;}**

**else** **{**$flag_1_base_staple **=** 1**;** **print** "\nHere\n!!"**;}**

**}**

**if** **(** $flag_CO **==** 1**)** **{**

$SingleStaples**{**$arr**[**0**]}** **=** $arr**[**0**];**

**my** @arrSingle **=** @**{**$SingleStaples**{**$arr**[**0**]}};}**

$flag_CO **=** 0**;**

$fullStapleHash**{**$keyNum**}{**$keyPos**}** **=** **\**@arr**;**

**}**

**close** **(**SL_FILE**);**

**my** $CheckForHalfCO **=** 0**;**

**my** $numOfCO **=** 0**;**

**my** $total **=** 0**;**

**my** $del **=** 0**;**

**my** $helix **=**0 **;**

**my** $position **=** 0**;**

**my** $refToARR**;** **my** $indexNum1**;** **my** $indexNum2**;**

**foreach** **my** $num1**(sort** **keys** %crossOverHash1**)** **{**

**my** @secondaryKeysNum2 **=** **sort** **keys** %**{**$crossOverHash1**{**$num1**}};**

**foreach** **my** $num2**(**@secondaryKeysNum2**)** **{**

**my** @thirdKeysPos **=** **sort** **keys** %**{**$crossOverHash1**{**$num1**}{**$num2**}};**

**foreach** **my** $pos1**(**@thirdKeysPos**)** **{**

**my**@fourthKeysPos**=sortkeys** %**{**$crossOverHash1**{**$num1**}{**$num2**}{**$pos1**}};**

**foreach** **my** $pos2 **(**@fourthKeysPos**)** **{**

$CheckForHalfCO**=** @**{**$crossOverHash1**{**$num1**}{**$num2**}{**$pos1**}{**$pos2**}};**

$total **+=** 1**;**

**if** **(** $CheckForHalfCO **!=** 8 **)**

**{**

$del **+=** 1**;}**

**else** **{**

$numOfCO **+=**1**;**

$crossOverHash2**{**$num1**}{**$num2**}{**$pos1**}{**$pos2**}=** $crossOverHash1**{**$num1**}{**$num2**}{**$pos1**}{**$pos2**};**

$refToARR**=** $crossOverHash2**{**$num1**}{**$num2**}{**$pos1**}{**$pos2**};**

$indexNum1 **=** $numOrderInShape**{**$num1**};**

$indexNum2 **=** $numOrderInShape**{**$num2**};**

**if** **(** **abs(**$num1**-**$num2**)==** 1 **&&** **abs(**$indexNum1**-**$indexNum2**)** **==** 1 **)** **{**

**if** **(**$indexNum1**>**$indexNum2**)** **{**

$helix **=** $indexNum1**;** **}**

**else** **{**

$helix **=** $indexNum2**;}**

**}**

**elsif** **(abs(**$num1**-**$num2**)** **!=** 1 **&&** **abs(**$indexNum1**-**$indexNum2**)** **==** 1**)** **{**

**if** **(**$indexNum1**>**$indexNum2**)** **{**

$helix **=** $indexNum1**;** **}**

**else** **{**

$helix **=** $indexNum2**;}**

**}** **else** **{**

$helix **=** **int** **((**$indexNum1 **+** $indexNum2**)/**2 **+** 0.5 **);}**

$position **=** **int** **((**$pos1 **+** $pos2**)/**2 **)** **;**

**push(**$refToARR**,**$helix **);**

**push(**$refToARR**,**$position**);**

**push(**$refToARR**,**91**);**

**}**

**}**

**}**

**}**

**}**

**my** $flag_Full_CO **=** 0**;**

**my** $index **=** 0**;**

**my** $NumOfST2CO **=** 0**;**

**my** $y**;** **my** $refStaplePart**;**

$CheckForHalfCO **=** 0**;**

**foreach** **my** $ref**(sort** **keys** %staplePartsRefAndCO**)** **{**

**my** $x **=** @**{**$staplePartsRefAndCO**{**$ref**}};**

**for** **(my** $i **=** 0**;** $i **<** $x **;** $i **+=** 1**)** **{**

**my** $refToCO **=** @**{**$staplePartsRefAndCO**{**$ref**}}[**$index**];**

$CheckForHalfCO **=** @**{**$refToCO**};**

**if** **(** $CheckForHalfCO **!=** 11 **)**

**{**

**if** **(** $ref **eq** $refToCO**->[**4**])** **{**

$refStaplePart **=** $refToCO**->[**4**];}**

**else** **{**

$refStaplePart **=** $refToCO**->[**5**];}**

**if** **(** $index **==** 0 **)** **{**

$refToCO **=** **shift(**$staplePartsRefAndCO**{**$ref**});}**

**elsif** **(** $index **==** 1 **)** **{**

$refToCO **=** **pop(**$staplePartsRefAndCO**{**$ref**});}**

**}**

**elsif** **(** $CheckForHalfCO **==** 11 **)**

**{**

$flag_Full_CO **=** 1**;** **}**

**else**

**{**

**print** "ERROR!!!\n"**;}**

$y **=** @**{**$staplePartsRefAndCO**{**$ref**}};**

**if** **(** $y **==** 1**)** **{**

$index **=** 0**;}**

**else** **{**

$index **+=** 1**;}**

**}**

**if** **(** $flag_Full_CO **==** 0 **)** **{**

$SingleStaples**{**$ref**}** **=** $refStaplePart**;**

**delete** $staplePartsRefAndCO**{**$ref**};}**

**if** **(exists** $staplePartsRefAndCO**{**$ref**}** **)** **{**

$y **=** @**{**$staplePartsRefAndCO**{**$ref**}};**

**if** **(**$y **==** 2 **)** **{**$NumOfST2CO **+=** 1**;}**

**}**

$index **=** 0**;**

$flag_Full_CO **=** 0**;**

**}**

**print** "NumOfK1 - $NumOfK1\n NumOfK2 - $NumOfK2\n NumOfK3 - $NumOfK3\n NumOfJ1 - $NumOfJ1\n"**;**

###########################################################################

#Part3-folding-simulation.

###########################################################################

**my** $tmp **;** **my** $stRef **;** **my** $refST**;**

**my** $stRefString**;**

**my** $stRefStringSN**;**

**my** $refToCO**;my** $tmpRef**;**

**my** $delta **=** 0**;**

**my** $current_TMP **=** 0**;**

**my** $singleST_folded **=** 0**;**

**my** $COFoldedST **=** 0**;**

**my** $COFolded **=** 0**;**

**my** %TMP_COST**;** **my** %TMP_ST**;** **my** %TMP_CO**;**

**my** $NumOfFoldedStaples **=** 0**;**

**my** $NumOfNONFoldedStaples **=** 0**;**

**my** $percentageOfFoldingStaples **=** 0**;**

**my** $numOfElements **=** **keys** %staplePartsRefAndCO**;**

**my** $numOfSingleStaples **=** **keys** %SingleStaples**;**

**my** $PrecentageOfFoldedST **=** "%FoldingSummary.txt"**;**

**open(**FOLDEDST**,** ">>$PrecentageOfFoldedST"**)** **or** **die** "Can't read file $PrecentageOfFoldedST [$!]\n"**;**

**print** FOLDEDST "Folding Results for $jasonFileName\n"**;**

**print** FOLDEDST "NumHelix: $numOfHelixes Lenght: $helixLenght\n"**;**

**print** FOLDEDST "Total Num of staples parts: $totalNumOfStapleParts\n"**;**

**print** FOLDEDST "Total number of CO: $numOfCO\n"**;**

**print** FOLDEDST "Total number of Single Staples: $numOfSingleStaples\n"**;**

**print** FOLDEDST "Total number of staples that builds 2 CO: $NumOfST2CO\n"**;**

**print** FOLDEDST "TMP,Num Of NON Folded Staples Parts,Num of Folded staples parts,% of folded staples parts,Num of Folded ST,Num of Folded CO Staples,Num of folded CO,\n"**;**

**for** **(**$tmp **=** $initialTMP **;** $tmp **>=** $finalTMP **;** $tmp **-=** 1**)** **{**

# checking CO

**foreach** **my** $num1**(sort** **keys** %crossOverHash2**)** **{**

**my** @secondaryKeysNum2 **=** **sort** **keys** %**{**$crossOverHash2**{**$num1**}};**

**foreach** **my** $num2**(**@secondaryKeysNum2**)** **{**

**my** @thirdKeysPos **=** **sort** **keys** %**{**$crossOverHash2**{**$num1**}{**$num2**}};**

**foreach** **my** $pos1**(**@thirdKeysPos**)** **{**

**my** @fourthKeysPos **=** **sort** **keys** %**{**$crossOverHash2**{**$num1**}{**$num2**}{**$pos1**}};**

**foreach** **my** $pos2 **(**@fourthKeysPos**)** **{**

$refToCO **=** $crossOverHash2**{**$num1**}{**$num2**}{**$pos1**}{**$pos2**};**

**&**checkCO**(**$refToCO**,**$tmp**,\**%MatrixJunctionType**);**

**}**

**}**

**}**

**}**

$current_TMP **=** $tmp**;**

**foreach** $stRefStringSN**(sort** **keys** %SingleStaples**)**

**{**

$refST **=** $SingleStaples**{**$stRefStringSN**};**

**if** **(** $refST**->[**9**]** **>=** $current_TMP **&&** $refST**->[**10**]** **!=** 1 **)** **{**

$refST**->[**10**]** **=** 1 **;**

**my** @arrSingle **=** @**{**$SingleStaples**{**$refST**}};**

**if** **(** **exists** $TMP_ST**{** $current_TMP**}** **){**

$TMP_ST**{**$current_TMP**}** **=** $TMP_ST**{**$current_TMP**}** **+** 1**;}**

**else** **{**

$TMP_ST**{**$current_TMP**}** **=** 1**;}**

**if** **(** **exists** $TMHash**{** $current_TMP**}** **){**

$TMHash**{**$current_TMP**}** **=** $TMHash**{**$current_TMP**}** **+** 1**;}**

**else** **{**

$TMHash**{**$current_TMP**}** **=** 1**;}**

**}**

**}**

**if** **(exists** $TMHash**{**$tmp**})** **{**

$NumOfFoldedStaples **+=** $TMHash**{**$tmp**};}**

**if** **(exists** $TMP_COST**{**$tmp**})** **{**

$COFoldedST **+=** $TMP_COST**{**$tmp**};}**

**if** **(exists** $TMP_ST**{**$tmp**})** **{**

$singleST_folded **+=** $TMP_ST**{**$tmp**};}**

**if** **(exists** $TMP_CO**{**$tmp**})** **{**

$COFolded **+=** $TMP_CO**{**$tmp**};}**

$NumOfNONFoldedStaples **=** $totalNumOfStapleParts **-** $NumOfFoldedStaples**;**

$percentageOfFoldingStaples**=** **((**$NumOfFoldedStaples**/**$totalNumOfStapleParts**)***100**);**

**Print**FOLDEDST "$tmp,$NumOfNONFoldedStaples,$NumOfFoldedStaples,$percentageOfFoldingStaples,$singleST_folded,$COFoldedST,$COFolded\n"**;**

**}**

**close** **(**FOLDEDST**);**

# printing junction destribution map

**&**printingJunctionsTypeMap **(\**%MatrixJunctionType**);**

#--------------------------------FUNCTIONS---------------------------------

**sub** convertFromStringToRef **{**

**my** $stRefString **=** $_**[**0**];**

**my** $i **=** 4**;**

$refToCO **=** $staplePartsRefAndCO**{**$stRefString**}->[**0**];**

**while** **(**$stRefString **ne** $refToCO**->[**$i**])** **{**

$i **+=** 1**;}**

$stRef **=** $refToCO**->[**$i**];**

**return** $stRef**;**

**}**

#--------------------------------------------------------------------------

**sub** printingJunctionsTypeMap **{**

**my** $refToMatrix **=** $_**[**0**];**

**my** %copyMartrix **=** %**{**$refToMatrix**};**

**my** $outPutFile **=** "JunctionsTypeMapping.txt"**;**

**my** $rows **=** $numOfHelixes*****18**;**

**my** $col **=** $helixLenght*****4**;**

**my** $junctionType**;**

**my** $TmTmp**;**

**my** $refToARR**;**

**my** $helixDrawing**;**

**open(**OUTPUT**,** ">>$outPutFile"**)** **or** **die** "Can't read file $outPutFile [$!]\n"**;**

**my** $img **=** GD**::**Simple**->**new**(**$col**,** $rows**);**

$img**->**fgcolor**(**'black'**);**

$img**->**rectangle**(**0**,** 0**,** $col**-**1**,** $rows**-**1**);**

**foreach** $helix **(sort** **{** $numOrderInShape**{**$a**}** **<=>** $numOrderInShape**{**$b**}** **}** **keys** %numOrderInShape**)** **{**

**if** **(** **(**$helix**%**2**)** **==** 0 **)** **{**

$helixDrawing **=** $helix*****18**-**3**;}**

**else**

**{**

$helixDrawing **=** $helix*****18**;}**

**for** **(my** $position **=** 0**;** $position **<** $col**;** $position**++)** **{**

**if** **(exists** $copyMartrix**{**$helix**}{**$position**})** **{**

$refToARR **=** $copyMartrix**{**$helix**}{**$position**};**

$TmTmp **=** **(**$refToARR**->[**0**])****7**/(**1000000000000**)** **+** **int((**$refToARR**->[**0**])/**3**);**

$junctionType **=** $refToARR**->[**1**];**

$img**->**bgcolor**(**'green'**);**

$img**->**fgcolor**(**'green'**);**

**if** **(**$junctionType **eq** "K1"**)** **{**

$img**->**bgcolor**(**'green'**);**

$img**->**fgcolor**(**'green'**);**

$img**->**rectangle**(**$position*****4**,** $helixDrawing**,** $position*****4**+**$TmTmp**,** $helixDrawing**+**$TmTmp**);}**

**elsif** **(**$junctionType **eq** "K2"**)** **{**

$img**->**bgcolor**(**'blue'**);**

$img**->**fgcolor**(**'blue'**);**

$img**->**rectangle**(**$position*****4**,** $helixDrawing**,** $position*****4**+**$TmTmp**,** $helixDrawing**+**$TmTmp**);}**

**elsif** **(**$junctionType **eq** "K3"**)** **{**

$img**->**bgcolor**(**'red'**);**

$img**->**fgcolor**(**'red'**);**

$img**->**rectangle**(**$position*****4**,** $helixDrawing**,** $position*****4**+**$TmTmp**,** $helixDrawing**+**$TmTmp**);}**

**elsif** **(**$junctionType **eq** "J1"**)** **{**

$img**->**bgcolor**(**'darkviolet'**);**

$img**->**fgcolor**(**'darkviolet'**);**

$img**->**rectangle**(**$position*****4**,** $helixDrawing**,** $position*****4**+**$TmTmp**,** $helixDrawing**+**$TmTmp**);}**

**print** OUTPUT $copyMartrix**{**$helix**}{**$position**};**

**}**

**else** **{**

**print** OUTPUT " "**;**

**}**

**}**

**print** OUTPUT "\n"**;**

**}**

**close** **(**OUTPUT**);**

**open** **my** $out**,** '>'**,** 'img.png' **or** **die;**

**binmode** $out**;**

**print** $out $img**->**png**;**

**return;**

**}**

#--------------------------------------------------------------------------

**sub** checkCO **{**

**my** $refCO **=** $_**[**0**];**

**my** $tmpRef**;** **my** $CO2**;** **my** $refSTpart**;**

**my** $current_TMP **=** $_**[**1**];**

**my** $refToMatrix **=** $_**[**2**];**

**my** %copyMartrix **=** %**{**$refToMatrix**};**

**my** @arrTypeTMP**;**

**my** $i **=** 4**;**

**my** $numOfFoldedSt **=** 0**;**

**my** $numOfCO **=** 0**;**

**my** $Tj2 **=** 0**;**

**my** $Tj1 **=** 0**;**

**my** $flagFoldingSt **=** 0**;**

**my** $helix **=** 0**;**

**my** $position **=** 0**;**

**my** $delta **=** 0**;**

**my** $differenceTj **=** 0**;**

**if** **(** $refCO**->[**10**]** **>** $current_TMP **&&** **(**$refCO**->[**3**]** **!=** **-**1 **&&** $refCO**->[**3**]** **!=** 0**)** **)** **{**

$refCO**->[**3**]** **+=** 1**;**

$refCO**->[**10**]** **=** $current_TMP**;}**

**if** **(** $refCO**->[**2**]** **!=** 5 **&&** **(** $refCO**->[**0**]** **>=** $current_TMP **||** $refCO**->[**2**]** **==** 3 **||** $refCO**->[**2**]** **==** 4 **))** **{**

**if** **(** $refCO**->[**3**]** **==** 0 **||** $refCO**->[**3**]** **==** **-**1 **||** $refCO**->[**2**]** **==** 3 **||** $refCO**->[**2**]** **==** 4 **)** **{**

$helix **=** $refCO**->[**8**];**

$position **=** $refCO**->[**9**];**

$arrTypeTMP**[**0**]** **=** $current_TMP**;**

$arrTypeTMP**[**1**]** **=** $refCO**->[**1**];**

$MatrixJunctionType**{**$helix**}{**$position**}** **=** **\**@arrTypeTMP**;**

**for** **(**$i **=** 4**;** $i**<=**7**;** $i**++)** **{**

$flagFoldingSt **=** 0**;**

$refSTpart **=** $refCO**->[**$i**];**

**if** **(** $refSTpart**->[**10**]** **==** 0 **)** **{**

$refSTpart**->[**10**]** **=** 1**;**

$numOfFoldedSt **+=**1**;**

$flagFoldingSt **=** 1**;**

**}**

$refCO**->[**2**]** **=** 5**;**

$numOfCO **=** @**{**$staplePartsRefAndCO**{**$refSTpart**}};**

**if** **(**$numOfCO **!=** 1 **&&** $numOfCO **!=** 2 **)** **{**

**print** "Error: $numOfCO that a certain staple part builds\n"**;}**

**if** **(** $numOfCO **==** 2 **)** **{**

$CO2 **=** $staplePartsRefAndCO**{**$refSTpart**}->[**0**];**

**if** **(** $CO2 **==** $refCO**)** **{**

$CO2 **=** $staplePartsRefAndCO**{**$refSTpart**}->[**1**];}**

**if** **(** $CO2**->[**2**]** **!=** 5 **&&** $CO2**->[**0**]** **<** $current_TMP **)** **{**

**if** **(** $flagFoldingSt **==** 1 **)** **{**

$CO2**->[**2**]** **+=** 1**;**

**}**

$Tj2 **=** $CO2**->[**0**];**

$Tj1 **=** $refCO**->[**0**];**

$differenceTj **=** $Tj1 **-** $Tj2**;**

**if** **(**$differenceTj **>=** 4.5**)** **{**

$delta **=** **(**$differenceTj**)***0.9**;**

$CO2**->[**0**]** **+=** $delta**;**

**if** **(** $CO2**->[**1**]** **eq** "K3"**)** **{**

**if** **(** $CO2**->[**3**]** **eq** 0 **||** $CO2**->[**3**]** **eq** **-**1 **)** **{**

$CO2**->[**3**]** **=** **-**4**;**

$CO2**->[**10**]** **=** $current_TMP**;**

**}**

**elsif** **(** **(** $CO2**->[**3**]** **eq** **-**4 **||** $CO2**->[**3**]** **eq** **-**3 **||** $CO2**->[**3**]** **eq** **-**2 **)** **&&** **(** $CO2**->[**0**]** **>=** $current_TMP**)** **)** **{**

$CO2**->[**3**]** **+=** 1**;**

**if** **(**$CO2**->[**3**]** **eq** **-**1**)** **{**

**&**checkCO**(**$CO2**,** $current_TMP**,**$refToMatrix**);}**

**}**

**}**

**else** **{**

**if** **(** $CO2**->[**3**]** **eq** 0 **||** $CO2**->[**3**]** **eq** **-**1 **)** **{**

$CO2**->[**3**]** **=** **-**2**;**

$CO2**->[**10**]** **=** $current_TMP**;**

**}**

**elsif** **(** $CO2**->[**3**]** **eq** **-**2 **&&** $CO2**->[**0**]** **>=** $current_TMP **)** **{**

$CO2**->[**3**]** **+=** 1**;**

**if** **(**$CO2**->[**3**]** **eq** **-**1 **)** **{**

**&**checkCO**(**$CO2**,** $current_TMP**,** $refToMatrix**);}**

**}**

**}**

**}**

**if** **((**$CO2**->[**2**]** **==** 3 **||** $CO2**->[**2**]** **==** 4 **)** **&&** **(**$CO2**->[**3**]** **ne** **-**4 **))** **{**

**&**checkCO**(**$CO2**,** $current_TMP**,** $refToMatrix**);**

**}**

**}**

**}**

**}**

**if** **(** **exists** $TMP_COST**{**$current_TMP**}** **){**

$TMP_COST**{**$current_TMP**}** **=** $TMP_COST**{**$current_TMP**}** **+** $numOfFoldedSt**;}**

**else** **{**

$TMP_COST**{**$current_TMP**}** **=** $numOfFoldedSt**;}**

**if** **(** **exists** $TMHash**{** $current_TMP**}** **){**

$TMHash**{**$current_TMP**}** **=** $TMHash**{**$current_TMP**}** **+** $numOfFoldedSt**;}**

**else** **{**

$TMHash**{**$current_TMP**}** **=** $numOfFoldedSt**;}**

**if** **(** **exists** $TMP_CO**{** $current_TMP**}** **){**

$TMP_CO**{**$current_TMP**}** **=** $TMP_CO**{**$current_TMP**}** **+** 1**;}**

**else** **{**

$TMP_CO**{**$current_TMP**}** **=** 1**;}**

**}**

**}**

**elsif** **(**$refCO**->[**2**]** **!=** 5**)** **{**

**for** **(**$i **=** 4**;** $i**<=**7**;** $i**++)** **{**

$refSTpart **=** $refCO**->[**$i**];**

**if** **(**$refSTpart**->[**9**]** **>=** $current_TMP **&&** $refSTpart**->[**10**]** **!=** 1**)** **{**

$numOfFoldedSt **+=** 1**;**

$refSTpart**->[**10**]** **=** 1 **;**

$refCO**->[**2**]** **+=** 1**;**

**my** @arrSTpart **=** @**{**$refSTpart**};**

$numOfCO **=** $staplePartsRefAndCO**{**$refSTpart**};**

**if** **(** $numOfCO **==** 2 **)** **{**

$CO2 **=** $staplePartsRefAndCO**{**$refSTpart**}->[**0**];**

**if** **(** $CO2 **==** $refCO**)** **{**

$CO2 **=** $staplePartsRefAndCO**{**$refSTpart**}->[**1**];}**

**if** **(** $CO2**->[**2**]** **!=** 5 **&&** $CO2**->[**2**]** **!=** 4**)** **{**

$CO2**->[**2**]** **+=** 1**;**

**if** **(** $CO2**->[**2**]** **==** 3 **||** $CO2**->[**2**]** **==** 4**)** **{**

**&**checkCO**(**$CO2**,**$current_TMP**,**$refToMatrix**);}**

**}**

**}**

**if** **(** **exists** $TMP_COST**{**$current_TMP**}** **){**

$TMP_COST**{**$current_TMP**}** **=** $TMP_COST**{**$current_TMP**}** **+** 1**;}**

**else** **{**

$TMP_COST**{**$current_TMP**}** **=** 1**;}**

**if** **(** **exists** $TMHash**{** $current_TMP**}** **){**

$TMHash**{**$current_TMP**}** **=** $TMHash**{**$current_TMP**}** **+** 1**;}**

**else** **{**

$TMHash**{**$current_TMP**}** **=** 1**;}**

**}**

**}**

**}**

**return** $numOfFoldedSt**;**

**}**

#--------------------------------------------------------------------------

**sub** sortingJunctionAndTj **{**

**my** $RefToCO **=** $_**[**0**];**

**my** $pos1 **=** $_**[**1**];**

**my** $pos2 **=** $_**[**2**];**

**my** $RefToST**;** **my** $arm1**;** **my** $arm2**;**

**my** $refArmA**;** **my** $posArmA**;**

**my** @tmpArr**;**

**for** **(my** $i **=** 4**;** $i **<** 8**;** $i**++)** **{**

$RefToST **=** $RefToCO**->[**$i**];**

**push** **(**@tmpArr**,**$RefToST**->[**9**]);**

**}**

@tmpArr **=** **sort** **{**$b **<=>** $a**}** @tmpArr**;**

**my** @UniquetmpArr **=** uniq @tmpArr**;**

**my** $numOfElements **=** @UniquetmpArr**;**

**if** **(**$numOfElements **eq** 4**)** **{**

$refArmA **=** $RefToCO**->[**4**];**

$posArmA **=** 4**;**

$RefToST **=** $RefToCO**->[**6**];**

**if** **(** $RefToST**->[**9**]** **>** $refArmA**->[**9**])** **{**

$refArmA **=** $RefToST**;**

$posArmA **=** 6**;}**

$RefToST **=** $RefToCO**->[**$posArmA**+**1**];**

$arm1 **=** $RefToST**->[**9**];**

**my** $tempRef1**;**

**my** $tempRef2**;**

**if** **(** $posArmA **==** 4**)** **{**

$tempRef1 **=** $RefToCO**->[**6**];**

$tempRef2 **=** $RefToCO**->[**7**];**

**}**

**elsif** **(**$posArmA **==** 6**)** **{**

$tempRef1 **=** $RefToCO**->[**4**];**

$tempRef2 **=** $RefToCO**->[**5**];**

**}**

**else** **{** **print** "error : arm A pos\n;"**}**

**if** **(** $RefToST**->[**0**]** **!=** $refArmA**->[**0**]** **)** **{**

**if** **(** $tempRef1**->[**0**]** **==** $refArmA**->[**0**]** **)** **{**

$arm2 **=** $tempRef1**->[**9**];}**

**elsif** **(** $tempRef2**->[**0**]** **==** $refArmA**->[**0**]** **)** **{**

$arm2 **=** $tempRef2**->[**9**];}**

**else** **{print** "ERROR: ARM2 CO OF STAPLE!!!\n"**;}**

**}**

**else** **{**

**my** $coPOS**;**

**if** **(**$refArmA**->[**1**]** **==** $pos1 **||** $refArmA**->[**1**]** **==** $pos2**)**

**{**

$coPOS **=** $refArmA**->[**1**];**

**if** **(**$tempRef1**->[**2**]** **==** $coPOS**)** **{**

$arm2 **=** $tempRef1**->[**9**];**

**}**

**elsif** **(** $tempRef2**->[**2**]** **==** $coPOS **)** **{**

$arm2 **=** $tempRef2**->[**9**];**

**}**

**}** **elsif** **(**$refArmA**->[**2**]** **==** $pos1 **||** $refArmA**->[**2**]** **==** $pos2**)** **{**

$coPOS **=** $refArmA**->[**2**];**

**if** **(**$tempRef1**->[**1**]** **==** $coPOS**)** **{**

$arm2 **=** $tempRef1**->[**9**];**

**}**

**elsif** **(** $tempRef2**->[**1**]** **==** $coPOS **)** **{**

$arm2 **=** $tempRef2**->[**9**];**

**}**

**}**

**else** **{print** "ERROR: ARM2 CO OF SCAFF!!!\n"**;}**

**}**

**if** **(** $arm1 **!=** $tmpArr**[**1**]** **&&** $arm2 **!=** $tmpArr**[**1**]** **)** **{**

$RefToCO**->[**0**]** **=** $tmpArr**[**2**]** **+** 2**;**

$RefToCO**->[**1**]** **=** "K3"**;**

$NumOfK3 **+=** 1**;**

**}** **elsif** **(** $arm1 **==** $tmpArr**[**2**]** **||** $arm2 **==** $tmpArr**[**2**]** **)** **{**

$RefToCO**->[**0**]** **=** $tmpArr**[**2**]** **+** 7**;**

$RefToCO**->[**1**]** **=** "K2"**;**

$NumOfK2 **+=** 1**;**

**}** **elsif** **(** $arm1 **==** $tmpArr**[**3**]** **||** $arm2 **==** $tmpArr**[**3**]** **)** **{**

$RefToCO**->[**0**]** **=** $tmpArr**[**2**]** **+**7**;**

$RefToCO**->[**1**]** **=** "K1"**;**

$NumOfK1 **+=** 1**;**

**}**

**}**

**else** **{**

$RefToCO**->[**1**]** **=** "J1"**;**

$NumOfJ1 **+=** 1**;**

**if** **(**$numOfElements **>** 1**)** **{**

$RefToCO**->[**0**]** **=** **(**$UniquetmpArr**[**0**]+**$UniquetmpArr**[**1**])/**2**;}**

**else** **{**

$RefToCO**->[**0**]** **=** $UniquetmpArr**[**0**];}**

**}**

**}**

#--------------------------------------------------------------------------
